# Supplementary material for: Systematic review and meta-analysis of the associations between body mass index, prostate cancer, advanced prostate cancer, and prostate-specific antigen
Source: Cancer Causes Control. 2020 Mar 11;31(5):431–49. doi: 10.1007/s10552-020-01291-3 (PMC7105428; doi:10.1007/s10552-020-01291-3)
Supplement: Supplementary file 1 — Supplementary file1 (DOCX 442 kb) [file 10552_2020_1291_MOESM1_ESM.docx]

## Appendix 1: Methods to Derive Standard Effect Estimates

### Section 1: Statistical Analysis of BMI-Prostate Cancer Association

For all studies, an effect estimate and its standard error (SE) on the same scale (e.g. per 5 kg/m^2^ increase in BMI) are necessary for inclusion in a ‘continuous’ meta-analysis. For BMI-prostate cancer studies, the effect estimate could be an odds ratio (OR), hazard ratio (HR), risk ratio (RR), or standardised mean difference (SMD). In this Section, we describe how we estimated these effect measures from the available data reported in each study.

#### 1.A. Weighted regression for correlated outcomes

Many studies (n=34) (1–34) that examined the association between BMI and prostate cancer risk reported ORs or HRs for prostate cancer (often adjusted for potential confounders) at different levels of BMI, with reference to a baseline level. To estimate a linear effect from these categorical effect estimates, we used generalised least squares for trend estimation (GLST) as proposed by Greenland and Longnecker (35), using the GLST command in Stata (36). This method estimates the variance-covariance matrix of the log-ORs or log-HRs, accounting (with some assumptions) for the correlation between ORs or HRs. The correlation appears because all ORs or HRs in a study are relative to the same reference group.

For each study, the number of men with prostate cancer and total number of men (or total follow-up time) are required, as well as the mean BMI in each category of BMI (the exposure variable), in addition to the OR or HR and SE for each non-reference category of BMI. We centred BMI around the mean BMI in the reference group, such that the exposure variable was equal to 0 in the reference group, as required by the Stata command.

#### 1.B. Mean BMI not presented for each level of BMI

While almost all studies presented the range of BMI values in each category (e.g. 25-29.9 kg/m^2^), , the GLST calculation requires the mean BMI in each category of BMI. We estimated the mean BMI using the method presented by Chêne and Thompson (37). This method assumes a normal distribution for BMI. Although BMI is generally positively skewed, we considered the degree of skewness insufficient to materially bias the results.

#### 1.C. Mean differences presented

Some studies (n=13) (38–50) presented the mean BMI for all men with and without prostate cancer separately. For these studies, the standardised mean difference (SMD) was calculated using standard methods (51). SMDs are equivalent to log-ORs per SD increase in exposure (37). Therefore, these were divided by the pooled SD of BMI and multiplied by five to give the log-OR for a 5 kg/m^2^ increase in BMI.

#### 1.D. Matched studies using levels of BMI, and ORs or HRs not reported for levels

Some studies matched on key variables (e.g. age) to reduce confounding and presented the number of men with and without prostate cancer by level of BMI, but did not report suitable results for either including in the meta-analyses or for using with GLST (52–57). It would be possible to estimate ORs using the number of men in each level of BMI, but this would break the matching between cases and controls, introducing confounding by, for example, age. As such, we estimated the overall mean and SD of BMI for both men with and without prostate cancer in these studies using the method of Chêne and Thompson (37), then estimated the SMD in BMI for prostate cancer as in **Section 1.C**.

#### 1.E. Other information presented

Some studies (n=15) (58–72) presented the OR or HR per unit increase in BMI; these studies required no transformation or calculation other than multiplying the log-OR or log-HR by five to obtain estimates relating to a 5-unit increase in BMI.

Studies that did not present enough data to estimate an OR or HR with a SE (n=11) (73–83) had a P value extracted for use in an albatross plot.

### Section 2: Statistical Analysis of BMI-PSA Association

For inclusion in the continuous BMI-PSA meta-analysis, a linear regression coefficient with standard error was required from each study.

Most studies (n=12) (84–95) looking at the association between BMI and PSA gave the mean PSA values in each of several categories of BMI. In these studies, we first estimated the change in log-PSA for a 1 kg/m^2^ increase in BMI, β, using variance weighted least squares (VWLS) linear regression. This is a simplified version of GLST, where there is no correlation between effect measures in the different categories that needs to be accounted for. There is no correlation because the mean PSA is presented for each level of BMI independently, rather than each non-reference level being compared to a reference level.

The estimated coefficient from VWLS regression, β, (and its SE) was then converted to a change in log-PSA for a 5 kg/m^2^ increase in BMI by multiplying by 5. This estimate was used in the meta-analysis, and exponentiated in the forest plots to give the proportion change in PSA for a 5 kg/m^2^ increase in PSA. This can be interpreted as the percentage change in PSA by subtracting 1 and multiplying by 100:

|  | $\% change in PSA= 100\left( e^{5\beta}-1 \right)$ | (1) |
| --- | --- | --- |

For example, a β of -0.024 (seen in Japanese Americans in Waters, 2009 (96)) is equivalent to a percentage change in PSA of -11.3% for a 5 kg/m^2^ increase in BMI.

### Section 3: Transformations of PSA

Most BMI-PSA studies looked at the association between BMI and PSA, not log-PSA, requiring the conversion of the mean and SD of PSA to the mean and SD of log-PSA. A similar transformation was also required when the median PSA and SD were presented, and when the geometric mean and SD were presented. In this Section we describe how we estimated the mean and SD of log-PSA from reported results in each study.

#### 3.A. Mean and SD of PSA presented

Where means and SDs of PSA were presented, we used the equations presented in Higgins et al. (2008) (97) to transform the mean and SD of the observed PSA to the mean and SD of log-PSA.

#### 3.B. Median PSA presented

One paper (95) reported the median PSA with an interquartile range. Assuming a log-normal distribution, the median PSA is equal to the exponentiated mean of log-PSA. The inter-quartile range (IQR) was used to estimate the SD of observed PSA, as the first and third quartiles for PSA are the exponentiated quartiles for log-PSA:

|  | $\sigma_{o}=\frac{\ln\left( \mathrm{IQR}_{u} \right)-\ln\left( \mathrm{IQR}_{l} \right)}{1.35}$ | (2) |
| --- | --- | --- |

where IQR_u_ and IQR_l_ are the upper and lower bounds of the interquartile range respectively and 1.35 is the approximate number of SDs in in an IQR (98).

#### 3.C. Geometric mean PSA presented

Two papers (89,90) reported the geometric mean and (arithmetic) SD of PSA or the SE of the mean PSA for each level of BMI, rather than the arithmetic mean and SD. The geometric mean of a distribution is by definition equal to the exponentiated mean of the logged distribution (97), so the required mean log-PSA is simply calculated as the logarithm of the reported geometric mean. If the SE of the mean PSA was presented, we estimated the SD of PSA using the following formula, where N is the total number of participants used to estimate the SE:

|  | $\mathrm{SE}=\frac{\sigma}{\sqrt{N}}$ | (3) |
| --- | --- | --- |

#### 3.D. Ratio of geometric means presented

Two studies (91,92) presented the ratio (or percentage change) of geometric means of PSA with 95% CIs for two levels of BMI with respect to a baseline level of BMI (overweight and obese versus normal-weight). These effects are equivalent to the exponentiated difference in log-PSA between the men who were normal weight and those who were overweight or obese. Although the geometric means of log-PSA for each level of BMI were also presented, the SDs of log-PSA (which are required for use of the method presented in **3.C**) were not. We devised an approach to estimate these SDs using the ratios and their SEs, which made two assumptions:

1. Log-PSA was normally distributed within each level of BMI
2. The variance of log-PSA in the second level of BMI was equal to the mean of the variances of log-PSA for the first and third BMI levels

These assumptions were necessary due to mean ratios providing effect estimates and SEs for all but one level of BMI: the baseline BMI level does not have an effect estimate or SE since it is included in all other estimates. The full derivation of this approach to estimating the log-PSA SDs is available in **appendix 7** of Harrison’s PhD thesis (99). Once the SDs were estimated, VWLS was used to compute the change in log-PSA for a 5 kg/m^2^ increase in BMI.

#### 3.E. Other information presented

Four studies (96,100–102) presented the log-PSA change per unit increase in BMI; these studies required no transformation or calculation other than multiplying the effect estimate by five to obtain estimates relating to a 5-unit increase in BMI.

Fourteen studies (103–116) presented information where a regression coefficient and SE could not be estimate and so could not be included in the meta-analysis. As such, an appropriate P value was taken from each study and used in the albatross plot.

## Appendix 2: Risk of Bias Questions

Each section (1 to 6) requires a risk of bias; low, medium, high, critical or unclear. The questions in each section help in identifying risk of bias, and are designed so a “yes” answer implies some risk of bias. An overall risk of bias was given based on the maximum risk of bias each section could contribute.

1. Bias due to confounding
   1. Is the mean age of the cases/controls NOT within 5 years, or is age NOT adjusted for in the analysis?
   2. Are there any other confounders likely to cause bias that have not been accounted for? SES, ethnicity etc.
2. Bias in selection of participants?
   1. Were the participants recruited in a way likely to cause bias? e.g. controls recruited in an obesity clinic
   2. Are the participants not representative of the general population of men in any way? e.g. All men had pituitary tumours
   3. Are there any other ways in which the selection of participants could cause bias?
3. Bias due to missing data?
   1. Cohort: Was the follow-up time insufficient to allow for a diagnosis of prostate cancer? e.g. less than 5 years
   2. Cohort: Were there differences in baseline measures of age and BMI between those lost to follow-up and not lost?
   3. Cohort: Were the baseline measures of age and BMI NOT presented for those lost to follow-up?
   4. Case-control: Were there non-responders that could have caused bias in the study?
4. Bias in measurement of outcome?
   1. Was prostate cancer or PSA measured in a way that could have caused bias? e.g. poor follow-up of obese men
   2. Could the participants without prostate cancer have had cancer? e.g. if controls were not assessed for cancer
5. Bias in measurement of exposure?
   1. Was BMI measured differently between cases and controls? e.g. controls were self-reported, cases were measured
6. Bias due to selective reporting?
   1. Was there selective reporting in the study which could have caused bias? e.g. were results only for particular subgroups reported, or did the reported results deviate from a pre-specified analysis plan?

## Appendix 3: Individual Participant Data Imputation and Analysis

### 3.1 Description of Studies Providing Individual Participant Data

#### Krimpen

In the Krimpen study (117), men aged 50-75 years were recruited from all general practices in Krimpen aan den Ijssel, the Netherlands, between August 1995 and January 1998 in a longitudinal study to determine the prevalence of benign prostatic hypertrophy. Men were not screened for prostate cancer at baseline but may have had PSA tests as part of their general care. Prostate cancer status was recorded for those men who received biopsies (26% of all men), and those with prostate cancer at the beginning of the study were excluded. The last prostate cancer diagnosis was made in 2004. Data for PSA, age, family history of prostate cancer and BMI were recorded at baseline and three follow-up rounds, in this analysis only the baseline measures were used. Ethnicity was not recorded, although the study investigators informed us that more than 90% of men in the Krimpen study were Caucasian. We thus assumed that all men in Krimpen were of white ethnicity.

#### Prostate Cancer Prevention Trial

In the Prostate Cancer Prevention Trial (PCPT) (118), from January 1994 through May 1997, men with a PSA level under 3.0 ng/ml underwent randomisation to either finasteride (a 5α reductase inhibitor) or placebo in the USA. The men underwent annual DREs and measurement of PSA. At the end of 7 years, all the men in whom prostate cancer had not been diagnosed were offered an end-of-study biopsy; in total, 61% of men received a prostate biopsy over the course of the study. Data collection, including BMI, PSA age, ethnicity and family history of prostate cancer, and prostate-cancer assessments continued until June 2004. In the imputation model, PSA, age, family history of prostate cancer and BMI from baseline were used, and prostate cancer diagnosis from both follow-up and the end-of-study biopsy. Only the control arm of PCPT was used in the imputation, not the arm that received finasteride.

PCPT represents an important source of information for our study: because men were offered a prostate biopsy independent of their PSA, PCPT can be used to predict the prostate cancer status of men with an initial PSA below 3.0 ng/ml. As the imputation was only in men with an initially low PSA, the prediction is unlikely to be biased. However, in measuring the association between BMI and prostate cancer, conditioning on PSA will lead to collider bias, and in measuring the association between BMI and PSA conditioning on the outcome will attenuate the association (119). Therefore, PCPT was only used for the imputation of prostate cancer status (and other missing variables) in the three other studies, and not included in the meta-analysis.

#### Prostate, Lung, Colorectal, and Ovarian Cancer Screening Trial

In the Prostate, Lung, Colorectal, and Ovarian (PLCO) Cancer Screening Trial (120), men aged 55–74 years were enrolled at 10 screening centres in the USA between November 1993 and July 2001. Men were randomly assigned to the intervention – organised screening of annual PSA testing for 6 years and annual DRE for 4 years - or usual care, which sometimes included opportunistic screening. The screening test was positive if the man had a PSA above 4.0 ng/ml or if the DRE was positive, although the decision to offer a biopsy was made by local health-care providers. Screening was completed in October 2006. All incident prostate cancers through 13 years of follow-up or through to the end of 2009 were ascertained. Given the PSA threshold and number of men with prostate cancer, we assumed 24% of men received a prostate biopsy. Only participants in the screening arm of PLCO had a recorded PSA test (taken at screening), so only the screening arm was included here. In this analysis, PSA, age, family history of prostate cancer and BMI from baseline were used, and prostate cancer diagnosis on follow-up.

#### Prostate Testing for Cancer and Treatment

In the Prostate Testing for Cancer and Treatment (ProtecT) study (121), PSA screening was conducted between 1999 and 2009. Men with a PSA above 3.0 ng/ml and below 19.9 ng/ml were invited to biopsy, while men with a PSA above 20 ng/ml were referred to usual care. Given the PSA threshold, we assumed 15% of men received prostate biopsies. Participants with localised prostate cancer were offered randomisation to one of three treatments: prostatectomy, radical radiotherapy or active monitoring. BMI was only available for a subset of the participants included in an accompanying study, thus BMI had to be imputed for the remaining men. In this analysis, PSA, age, family history of prostate cancer and BMI from baseline, and only prostate cancers found after initial biopsy were used.

### 3.2 Data Cleaning

Data from all studies were imported into Stata (version 15.1) for analysis. We calculated age and BMI at baseline from available data if not provided. PSA was logarithmically transformed for analysis due to its log-normal distribution and our assumption that due to haemodilution, any change in BMI would give a percentage change in PSA, not an absolute change. For 32 men where PSA was recorded as 0.00 ng/ml, this was recoded as 0.02 ng/ml, a standard amount used when PSA is undetectable (122). We defined advanced prostate cancer as having locally advanced disease (a T-stage 3 or above, T≥3), nodal involvement (N=1) or metastases (M=1) (123). We excluded non-white participants from the dataset (N = 7,405, 7.3%), as there were relatively few participants that were in these groups, making imputation of missing variables unfeasible. In total, we analysed data from 1,661 men in the Krimpen study, 8,724 men in the PCPT study, 33,025 men in the PLCO study, and 41,412 men in the ProtecT study.

The risk of bias assessment used for the aggregate data studies (**Section** **2.4**) was used to assess the risk of bias for the individual participant data (IPD) studies.

### 3.3 Imputation of Missing Data

To address the potential for bias from screening for all associations, we imputed prostate cancer status for all men assumed not to have received a biopsy (N = 61,676, 81.1%). In addition, some men were missing data for BMI (N = 497, 0.7%), family history of prostate cancer (N = 5,855, 7.7%) and PSA (N = 2,622, 3.5%), all of which were also imputed. No participant was missing data for age. We assumed all variables were missing at random (MAR), conditional on PSA levels and other variables in the imputation models.

PCPT was used specifically in building the imputation model because it included men with low baseline PSA values, as this mirrors precisely where the missing outcome data was in ProtecT, Krimpen and PLCO. Men who were not biopsied (i.e. with missing outcome data) necessarily had PSA values below that of the biopsy threshold (either 3.0 or 4.0 ng/ml), making PCPT a good choice for information for imputation as it had data for men with low PSA values. As PCPT selected on PSA for entry into the study, the study would have bias in the associations between BMI, prostate cancer and PSA, as the outcome of the study is related to the probability of being in the study (119). However, when predicting prostate cancer status from PSA, there is no bias due to including participants based on their PSA status. As such, PCPT was used in the imputation, but not in the analyses.

As participants in PCPT were followed for 7 years, the incidence of prostate cancer will likely be higher in PCPT than if all men had been biopsied at recruitment. However, this should not bias the analyses of the imputed data, as the imputation model accounts for different baseline incidences of prostate cancer between studies, for example from having different lengths of follow-up.

The proportion of missing information for all variables is shown in **Table A1**. As data in PCPT was only used to impute missing values in the other studies, and was not included in any analyses, it is not included in the totals. There were 13,050 men with full information on all variables (17%), 55,587 men missing information for one variable (77%), 7,326 men missing information for two variables (9.6%), and 137 men missing information for three or four variables (0.2%). The percentage of men missing prostate cancer status is very high, but we believed that by using PCPT we could impute prostate cancer status without biasing the analyses. The other variables had relatively low levels of missingness.

Table A1 Summary of missing information

|  | Krimpen | PLCO | ProtecT | Total for analysis | PCPT |
| --- | --- | --- | --- | --- | --- |
| N | 1,661 | 33,025 | 41,412 | 76,098 | 8,724 |
| Missing data | | | | | |
| PCa (N [%]) | 1,235 (74.4) | 25,168 (76.2) | 35,273 (85.2) | 61,676 (81.0) | 0 (0.0) |
| BMI (N [%]) | 60 (3.6) | 385 (1.2) | 52 (0.1) | 497 (0.7) | 98 (1.1) |
| Log-PSA (N [%]) | 2 (0.1) | 2,620 (7.9) | 0 (0.0) | 2,622 (3.5) | 0 (0.0) |
| Family history PCa (N [%]) | 208 (12.5) | 797 (2.4) | 4,850 (11.7) | 5,855 (7.7) | 0 (0.0) |
| Missing data for: | | | | | |
| 0 variables | 359 (21.6) | 7,253 (22.0) | 5,438 (13.1) | 13,050 (17.1) | 8,626 (98.9) |
| 1 variable | 1,113 (67.0) | 22,696 (68.7) | 31,778 (76.7) | 55,587 (73.0) | 98 (1.1) |
| 2 variables | 175 (10.5) | 2,960 (9.0) | 4,191 (10.1) | 7,326 (9.6) | 0 (0.0) |
| 3 variables | 14 (0.8) | 110 (0.3) | 5 (0.0) | 129 (0.2) | 0 (0.0) |
| 4 variables | 0 (0.0) | 6 (0.0) | 0 (0.0) | 6 (0.0) | 0 (0.0) |
| *PCa = prostate cancer, N = number of participants, PCPT = Prostate Cancer Prevention Trial, PLCO = Prostate, Lung, Colorectal and Ovarian Cancer Screening Trial, ProtecT = Prostate Testing for Cancer and Treatment study* | | | | | |

For each study where biopsy status was not recorded, we determined whether participants likely received biopsies. The Krimpen study recorded the biopsy status for all participants, so no assumptions were necessary. For PLCO, we assumed participants received a biopsy if any recorded PSA measurement was above 4.0 ng/ml (the study threshold) or if they received a diagnosis of prostate cancer. For ProtecT, we assumed participants received a biopsy if their PSA was above 3.0 ng/ml (the study threshold) or if they received a diagnosis of prostate cancer. For PCPT, we assumed all participants received a biopsy; all participants without prostate cancer were invited to receive a biopsy at the end of the study regardless of PSA level. Although not all participants will have opted for a biopsy, we assumed that missing biopsies were missing completely at random. Therefore, assuming all participants had a biopsy should not bias any of the associations between BMI, prostate cancer and PSA, and therefore not bias the imputation model. For all men, if a biopsy was assumed to have been received and there was no diagnosis of prostate cancer, we assumed the man did not have prostate cancer. Prostate cancer status was treated as missing and therefore imputed if the man was not assumed to have had a prostate biopsy.

Multiple imputation by chained equations (MICE) was used to impute prostate cancer status, family history of prostate cancer, log-PSA and BMI where missing. The MI impute package in Stata was used for the imputation (124). The homoscedastic stratified method of imputation was used, allowing for study-specific intercepts but shared effect estimates. We decided against within-study imputation, since this would prevent the unbiased estimation of prostate cancer risk in PCPT from informing the missing data from the screening studies; a factor variable representing study was used in all imputation models instead (‘stratified imputation’). **Table A2** provides further details of the imputation models for all imputed variables. In total, each imputed dataset was the product of 1000 iterations, and 100 new datasets were created. Advanced prostate cancer status was imputed in a separate model in the same way as for prostate cancer, but replacing prostate cancer with advanced prostate cancer.

We checked whether there was a large amount of heterogeneity between studies that would invalidate the use of stratified imputation, and also whether there were interactions between BMI and age that should be included in the imputation model. There was little evidence of heterogeneity, and thus stratified imputation was used; equally, there was no evidence of an interaction between age and BMI, and we did not include this interaction in the final model.

Table A2: Imputation models

| Variable being imputed | Regression model | Variables included in regression |
| --- | --- | --- |
| BMI | Linear | Age, log-PSA, family history, prostate cancer, study |
| Log-PSA | Linear | Age, BMI, family history, prostate cancer, study |
| Family history | Logistic | Age, BMI, log-PSA, prostate cancer, study |
| Prostate cancer | Logistic | Age, BMI, log-PSA, family history, study |
| Advanced PCa | Logistic | Age, BMI, log-PSA, family history, study |

### 3.4 Imputation results

The summary statistics for each study, averaged across 100 imputed datasets, are shown in **Table A3**. This table is separated into men who we assumed received a biopsy (first 5 rows), did not receive a biopsy (next 5 rows), and all participants (other rows). PCPT was not included in the “Total” column. We assumed 14,422 men received a biopsy (19%), and of these, 6,240 had prostate cancer (43%) and 716 men had advanced prostate cancer (5.0%). By contrast, we assumed 61,676 men did not receive a biopsy (81%), and when imputed, on average 17,227 of these men had prostate cancer (28%) and 899 men had advanced prostate cancer (1.5%). These results seem plausible when considering men without a prostate cancer diagnosis generally had lower PSA values. These results are also compatible with the observed prevalence of incidental prostate cancer from a systematic review of autopsy studies, where approximately 30-40% of men aged 60 years had prostate cancer (125).

Table A3 Summary of included studies for imputed data, averaged over 100 imputed datasets

|  | Krimpen | PLCO | ProtecT | Total | PCPT |
| --- | --- | --- | --- | --- | --- |
| Men who were biopsied (prostate cancer status not imputed) | | | | | |
| Participants (% of total) | 426 (25.6) | 7,857 (23.8) | 6,139 (14.8) | 14,422 (19.0) | 8,724 (100) |
| PCa (%) | 58 (13.6) | 3,890 (49.5) | 2,292 (37.3) | 6,240 (43.3) | 1,307 (15.0) |
| Advanced PCa (%)* | 7 (1.6) | 488 (6.2) | 221 (3.6) | 716 (5.0) | 25 (0.3) |
| No PCa (%) | 368 (86.4) | 3,967 (50.5) | 3,847 (62.7) | 8,182 (56.7) | 7,417 (85.0) |
| Men who were not biopsied (prostate cancer status imputed) | | | | | |
| Participants (% of total) | 1,235 (74.4) | 25,168 (76.2) | 35,273 (85.2) | 61,676 (81.0) | NA |
| PCa (%) | 107 (8.7) | 9,571 (38.0) | 7,549 (21.4) | 17,227 (27.9) | NA |
| Advanced PCa (%)* | 8 (0.6) | 618 (2.5) | 273 (0.8) | 899 (1.5) | NA |
| No PCa (%) | 1,128 (91.3) | 15,597 (62.0) | 27,724 (78.6) | 44,449 (72.1) | NA |
| All participants (men who were and were not biopsied combined) | | | | | |
| Participants | 1,661 | 33,025 | 41,412 | 76,098 | 8,724 |
| PCa (%) | 165 (10.0) | 13,461 (40.8) | 9,841 (23.8) | 23,467 (30.8) | 1,307 (15.0) |
| Advanced PCa (%)* | 15 (0.9) | 1,106 (3.4) | 494 (1.2) | 1,615 (2.1) | 25 (0.3) |
| No PCa (%) | 1,496 (90.0) | 19,564 (59.2) | 31,571 (76.2) | 52,631 (69.2) | 7,417 (85.0) |
| Age (mean, [SD]) | 61.5 (6.6) | 62.7 (5.3) | 60.0 (5.5) | 61.2 (5.6) | 63.3 (5.7) |
| BMI (mean, [SD]) | 26.0 (3.0) | 27.6 (4.2) | 27.5 (4.0) | 27.5 (4.1) | 27.7 (4.1) |
| Log-PSA (mean, [SD]) | 0.27 (0.89) | 0.16 (0.88) | 0.10 (0.91) | 0.13 (0.90) | 0.02 (0.63) |
| Family history PCa (%) | 152 (9.2) | 2,541 (7.7) | 2,351 (5.7) | 5,044 (6.6) | 1,365 (15.7) |
| **Advanced prostate cancer defined as a T-score 3 or above (T≥3), nodal involvement (N=1) or metastases (M=1)*  *PCa = prostate cancer, N = number of participants, PCPT = Prostate Cancer Prevention Trial, PLCO = Prostate, Lung, Colorectal and Ovarian Cancer Screening Trial, ProtecT = Prostate Testing for Cancer and Treatment study* | | | | | |

To check whether the imputation gave a plausible result in each study, we created graphs showing the prostate cancer risk against log-PSA values for each study using cubic splines with knots at log-PSA values of -0.5, 0 and 0.5 (roughly quartiles of log-PSA), both before (**Figure A1**) and after (**Figure A2**) imputation. The curves were limited to the middle 98% of the values of log-PSA to remove outliers. The red vertical lines indicate PSA values of 3.0 ng/ml and 4.0 ng/ml, representing the biopsy threshold of ProtecT and PLCO respectively, which were censored below these values in the non-imputed plot (as the risk of diagnosed prostate cancer diagnosis dropped to 0 in these studies).

Before imputation, the plot indicates that PLCO and ProtecT followed similar trajectories of prostate cancer risk above 4.0 ng/ml (albeit with a greater absolute risk in PLCO), and that PCPT had a prostate cancer risk similar to ProtecT around the 3.0 ng/ml threshold. Krimpen, with fewer participants, had a variable prostate cancer risk.

After imputation, prostate cancer risk decreased as expected below the biopsy thresholds for ProtecT and PLCO, and the prostate cancer risk of Krimpen was less variable. We considered that these graphs gave no evidence the imputation was not valid, with respect to the likely association between PSA and prostate cancer risk.


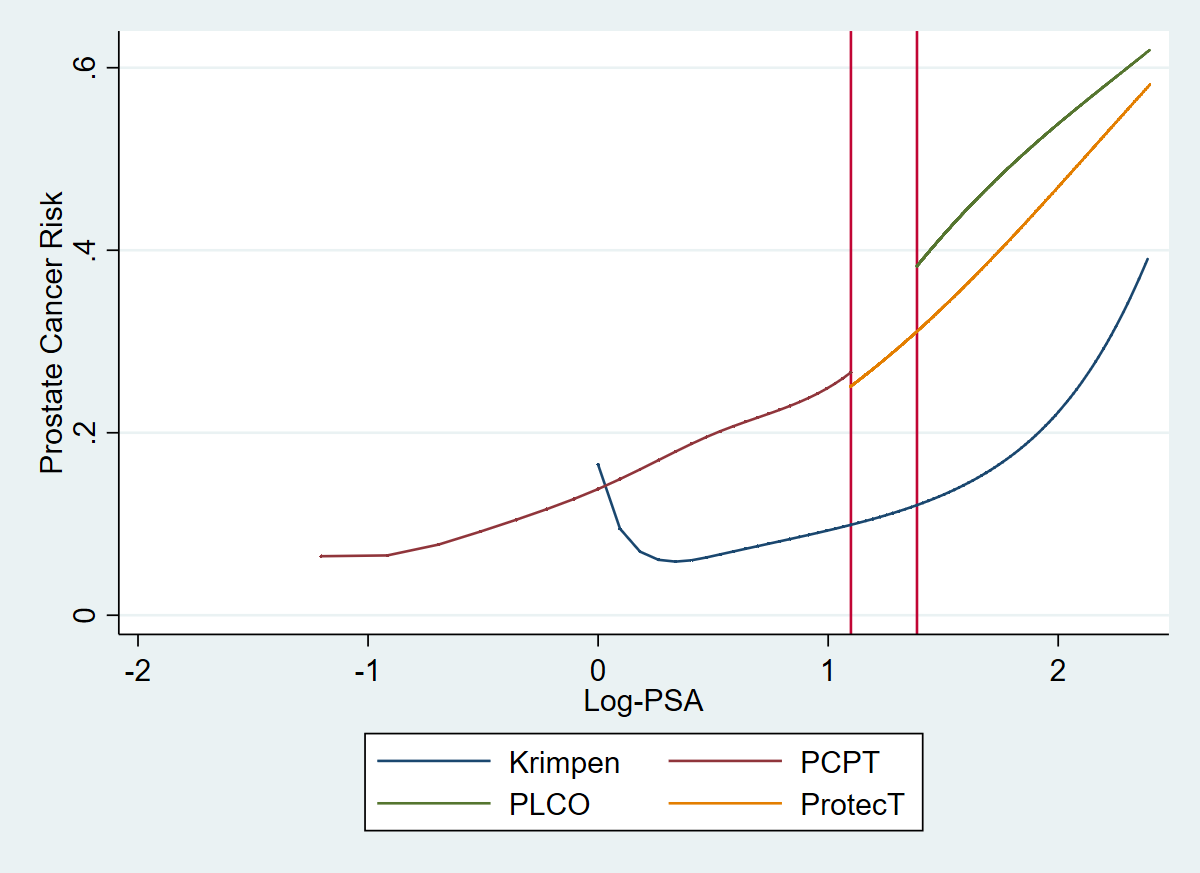


Figure A1: Line graph of log-PSA against prostate cancer risk, limited to the middle 98% of PSA values for each study, using restricted cubic splines of log-PSA. PLCO and ProtecT were limited to PSA values above 4.0 ng/ml and 3.0 ng/ml respectively as prostate cancer status was not observed lower than this. Knots at rough overall log PSA quartiles (-0.5, 0 and 0.5)


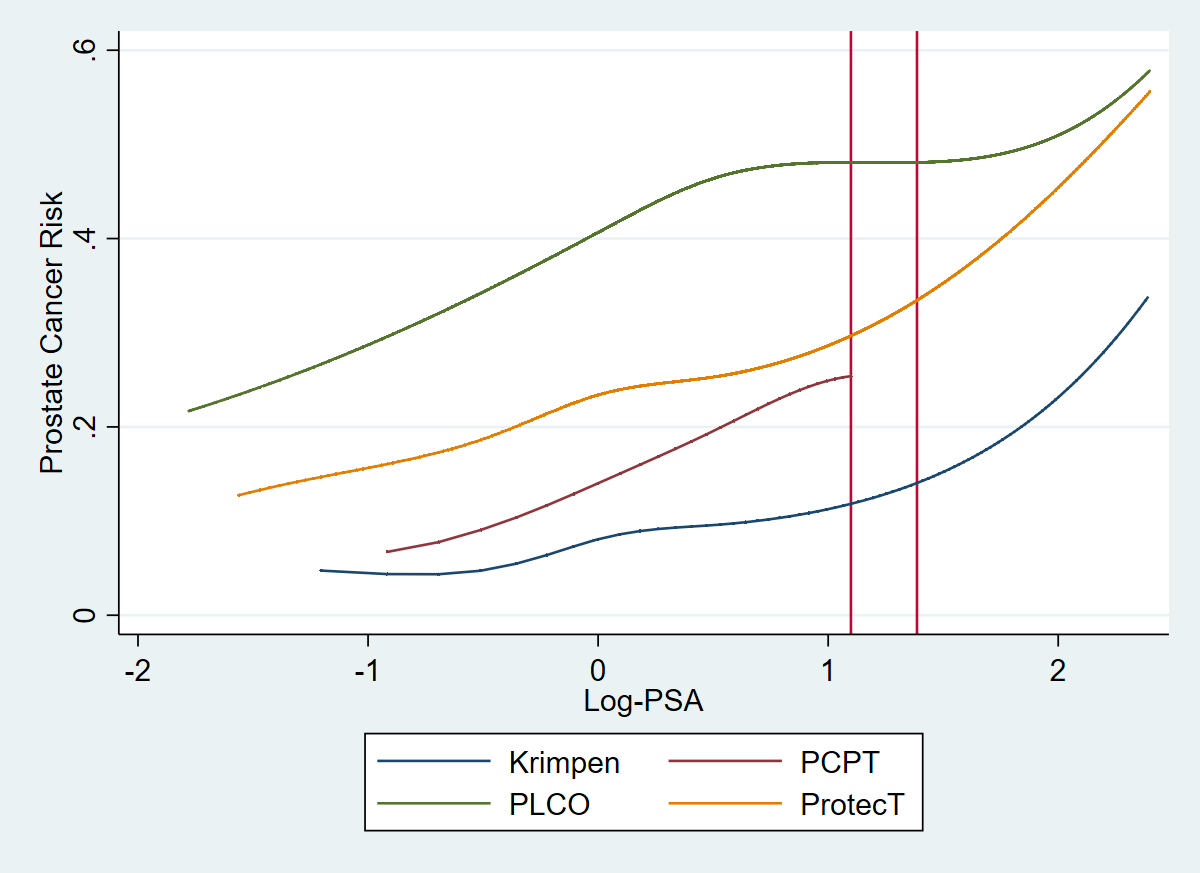


Figure A2: Line graph of prostate cancer risk against log-PSA, limited to the middle 98% of PSA values for each study, using restricted cubic splines of log-PSA. Knots at rough overall log PSA quartiles (-0.5, 0 and 0.5)

### 3.5 Analysis of Imputed Datasets

Once imputed, we estimated the linear associations between BMI and all outcomes using logistic or linear regression as appropriate for all IPD studies separately (excluding PCPT) and combined using Rubin’s rules. The results were converted to the OR for prostate cancer or advanced prostate cancer or regression coefficient for log-PSA for a 5 kg/m^2^ increase in BMI. PSA was not included as a covariable in the prostate cancer or advanced prostate cancer analyses, as we assumed that both BMI and prostate cancer affect PSA, such that including PSA as a covariable would cause collider bias. Prostate cancer (but not advanced prostate cancer) was included as a covariable in the PSA analysis. We conducted all analyses in Stata using the **mi estimate** command for analysing imputed datasets. These results were then added to the aggregate data study results before meta-analysis.

For the categorical analysis, we re-imputed using BMI as a 3-level categorical variable (normal weight, overweight and obese) so the analysis and imputation would be congenial. The ORs for prostate cancer and advanced prostate cancer and the MD for log-PSA were calculated for each study using the re-imputed datasets. All imputation and analysis methods were identical to the linear analysis, except for using a categorical version of BMI.

### 3.6 Complete Case Analysis Versus Imputed Analysis

We examined whether imputation of prostate cancer altered the effect estimates between BMI and prostate cancer, and BMI and advanced prostate cancer, for the IPD studies, anticipating that imputation would reduce bias. We conducted a complete case analysis without imputation for each study, assuming men without a diagnosis of prostate cancer did not have prostate cancer, and compared the results with those from the imputed analysis.

#### BMI and prostate cancer

The average OR for prostate cancer for a 5 kg/m^2^ increase in BMI for the IPD alone was estimated to be 0.94 (95% CI 0.91 to 0.98) for the complete case (non-imputed) analysis, and 1.00 (95% CI 0.97 to 1.03) for the imputed analysis, **Figure A3**. All three IPD study results were pulled closer to the null after imputation, possibly indicating that without imputation, the results of these studies would be biased downwards, likely because of the inverse relationship between BMI and PSA. The precision of the effect sizes was largely unaltered by imputation; only the effect sizes changed.


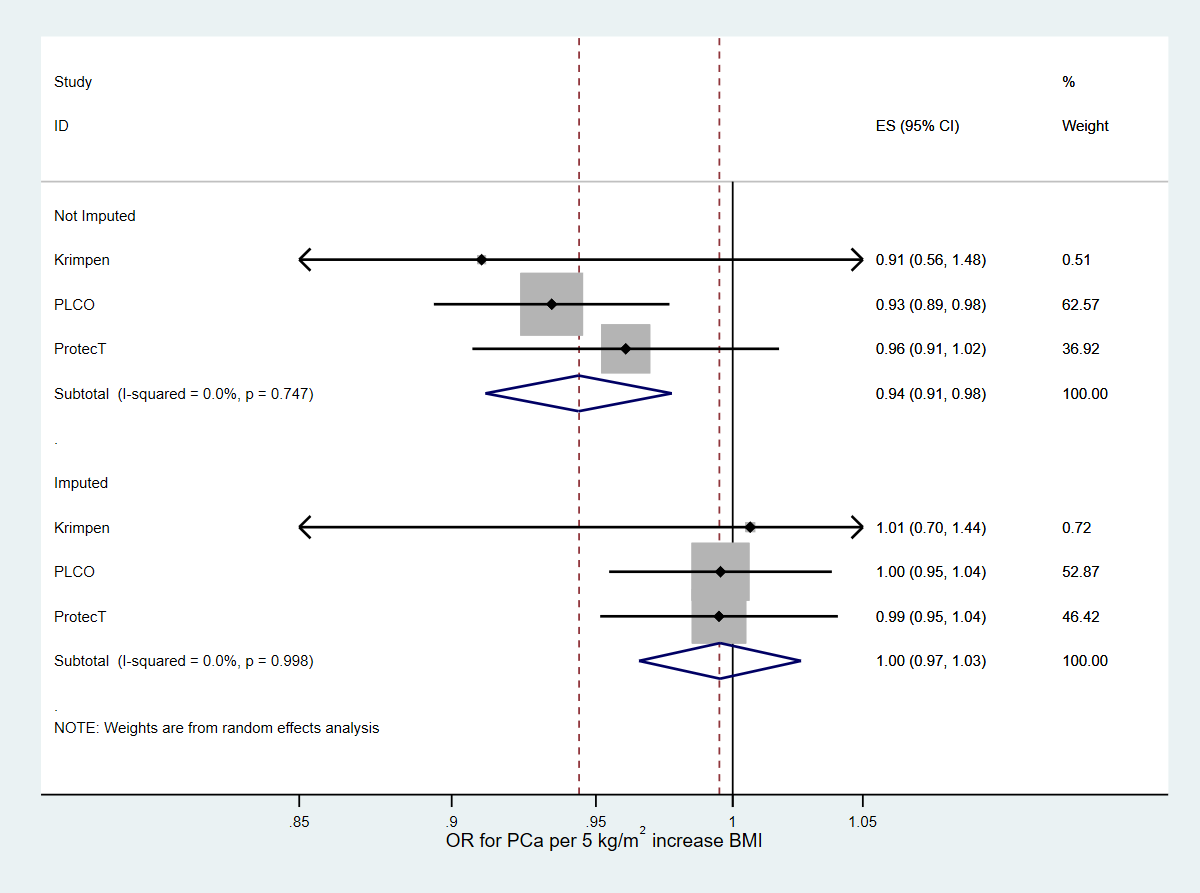


Figure A3: Forest plot for the association between BMI and prostate cancer for IPD studies, complete case analysis without imputation (above) and with imputation (below)

#### BMI and advanced prostate cancer

The average OR for advanced prostate cancer for a 5 kg/m^2^ increase in BMI for the IPD alone was estimated to be 0.98 (95% CI 0.89 to 1.08) for the complete case (non-imputed) analysis, and 1.02 (95% CI 0.93 to 1.11) for the imputed analysis, **Figure A4**. The results from Krimpen and PLCO were drawn closer to the null, as was the case with the prostate cancer analyses above. The estimated OR from Protect increased very slightly to at 1.01 but with a slightly narrower CI. This also suggests the complete case analyses were likely biased downwards. Again, the precision of the effect sizes was largely unaltered by imputation; only the effect sizes changed.


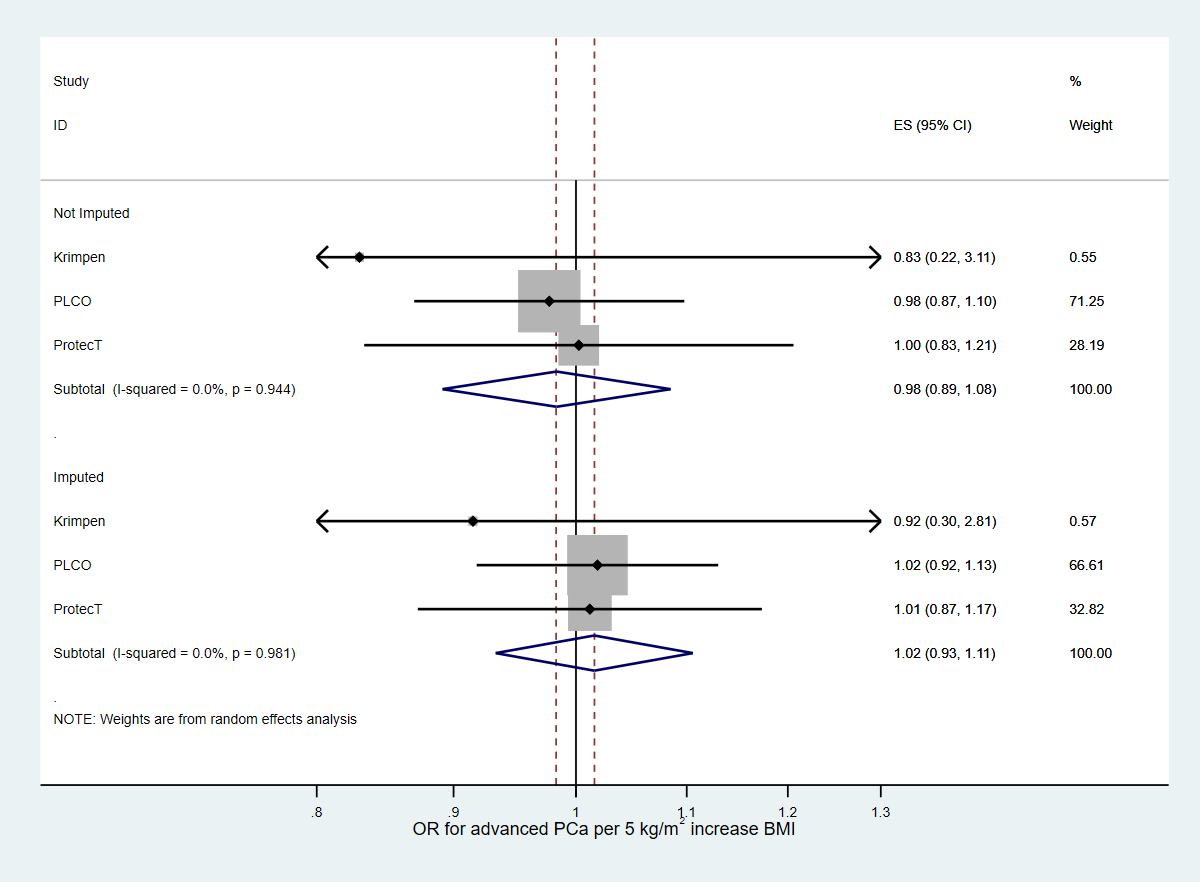


Figure A4: Forest plot for the association between BMI and advanced prostate cancer for IPD studies, complete case analysis without imputation (above) and with imputation (below)

## References

1. Severson RK, Grove JS, Nomura a M, Stemmermann GN. Body mass and prostatic cancer: a prospective study. BMJ. 1988;297(6650):713–5.

2. Mills PK, Beeson WL, Phillips RL, Fraser GE. Cohort study of diet, lifestyle, and prostate cancer in adventist men. Cancer [Internet]. 1989;64(3):598–604. Available from: http://onlinelibrary.wiley.com/doi/10.1002/1097-0142(19890801)64:3%3C598::AID-CNCR2820640306%3E3.0.CO;2-6/abstract%5Cnhttp://onlinelibrary.wiley.com/doi/10.1002/1097-0142(19890801)64:3%3C598::AID-CNCR2820640306%3E3.0.CO;2-6/abstract%5Cnhttp://onlinelibrary.wiley.co

3. Andersson S-O, Wolk A, Bergstrom R, Adami H-O, Engholm G, Englund A, et al. Body Size and Prostate Cancer: A 20-Year Follow-up Study Among 135006 Swedish Construction Workers. JNCI J Natl Cancer Inst [Internet]. 1997;89(5):385–9. Available from: http://jnci.oxfordjournals.org/cgi/doi/10.1093/jnci/89.5.385

4. Cerhan JR, Torner JC, Lynch CF, Rubenstein LM, Lemke JH, Cohen MB, et al. Association of smoking, body mass, and physical activity with risk of prostate cancer in the Iowa 65+ Rural Health Study (United States). Cancer Causes Control [Internet]. 1997;8(2):229–38. Available from: http://www.ncbi.nlm.nih.gov/pubmed/9134247

5. Giovannucci E, Rimm EB, Stampfer MJ, Colditz GA, Willett WC. Height, body weight, and risk of prostate cancer. Cancer Epidemiol Biomarkers Prev [Internet]. 1997;6(8):557–63. Available from: http://www.ncbi.nlm.nih.gov/pubmed/9264267%5Cnhttp://cebp.aacrjournals.org/content/6/8/557.full.pdf

6. Lund Nilsen TI, Vatten LJ. Anthropometry and prostate cancer risk: A prospective study of 22,248 Norwegian men. Cancer Causes Control [Internet]. 1999;10(4):269–75. Available from: http://www.ncbi.nlm.nih.gov/entrez/query.fcgi?cmd=Retrieve&db=PubMed&dopt=Citation&list_uids=10482485

7. Villeneuve PJ, Johnson KC, Kreiger N, Mao Y, Paulse B, Dewar R, et al. Risk factors for prostate cancer: Results from the Canadian National Enhanced Cancer Surveillance System. Cancer Causes Control. 1999;10(5):355–67.

8. Lee IM, Sesso HD, Paffenbarger RS. A prospective cohort study of physical activity and body size in relation to prostate cancer risk (United States). Cancer Causes Control [Internet]. 2001;12(2):187–93. Available from: http://dx.doi.org/10.1023/A:1008952528771

9. Sharpe CR, Siemiatycki J. Joint effects of smoking and body mass index on prostate cancer risk. Epidemiology [Internet]. 2001;12(5):546–51. Available from: http://www.ncbi.nlm.nih.gov/pubmed/11505174

10. Engeland A, Tretli S, Bjørge T. Height, body mass index, and prostate cancer: a follow-up of 950000 Norwegian men. Br J Cancer [Internet]. 2003;89(7):1237–42. Available from: http://www.nature.com/doifinder/10.1038/sj.bjc.6601801

11. Jonssoni F, Wolk A, Pedersen NL, Lichtenstein P, Terry P, Ahlbom A, et al. Obesity and hormone-dependent tumors: Cohort and co-twin control studies based on the Swedish Twin Registry. Int J Cancer [Internet]. 2003;106(4):594–9. Available from: http://www.ncbi.nlm.nih.gov/pubmed/12845658

12. Dal Maso L, Zucchetto a, La Vecchia C, Montella M, Conti E, Canzonieri V, et al. Prostate cancer and body size at different ages: an Italian multicentre case-control study. Br J Cancer [Internet]. 2004;90(11):2176–80. Available from: http://www.pubmedcentral.nih.gov/articlerender.fcgi?artid=2409495&tool=pmcentrez&rendertype=abstract

13. Friedenreich CM, McGregor SE, Courneya KS, Angyalfi SJ, Elliott FG. Case-control study of anthropometric measures and prostate cancer risk. Int J Cancer [Internet]. 2004;110(2):278–83. Available from: http://www.ncbi.nlm.nih.gov/pubmed/15069694

14. Bradbury BD, Wilk JB, Kaye JA. Obesity and the risk of prostate cancer (United States). Cancer Causes Control [Internet]. 2005;16(6):637–41. Available from: http://www.embase.com/search/results?subaction=viewrecord&from=export&id=L41110953%5Cnhttp://dx.doi.org/10.1007/s10552-005-0383-6%5Cnhttp://sfx.library.uu.nl/sfx?sid=EMBASE&issn=09575243&id=doi:10.1007/s10552-005-0383-6&atitle=Obesity+and+the+risk+of+prostate

15. Kuriyama S, Tsubono Y, Hozawa A, Shimazu T, Suzuki Y, Koizumi Y, et al. Obesity and risk of cancer in Japan. Int J Cancer. 2005;113(1):148–57.

16. LIU X, RYBICKI BA, CASEY G, WITTE JS. Relationship Between Body Size and Prostate Cancer in a Sibling Based Case-Control Study. J Urol [Internet]. 2005;174(6):2169–73. Available from: http://linkinghub.elsevier.com/retrieve/pii/S0022534701689390

17. Oh SW, Yoon YS, Shin SA. Effects of excess weight on cancer incidences depending on cancer sites and histologic findings among men: Korea National Health Insurance Corporation study. J Clin Oncol [Internet]. 2005;23(21):4742–54. Available from: http://www.jco.org/cgi/doi/10.1200/JCO.2005.11.726

18. Porter MP, Stanford JL. Obesity and the risk of prostate cancer. Prostate. 2005;62(April 2004):316–21.

19. Baillargeon J, Platz EA, Rose DP, Pollock BH, Ankerst DP, Haffner S, et al. Obesity, adipokines, and prostate cancer in a prospective population-based study. Cancer Epidemiol Biomarkers Prev. 2006;15(7):1331–5.

20. Kurahashi N, Iwasaki M, Sasazuki S, Otani T, Inoue M, Tsugane S. Association of body mass index and height with risk of prostate cancer among middle-aged Japanese men. Br J Cancer [Internet]. 2006;94(5):740–2. Available from: http://www.pubmedcentral.nih.gov/articlerender.fcgi?artid=2361195&tool=pmcentrez&rendertype=abstract

21. Lukanova A, Björ O, Kaaks R, Lenner P, Lindahl B, Hallmans G, et al. Body mass index and cancer: Results from the Northern Sweden Health and Disease Cohort. Int J Cancer [Internet]. 2006;118(2):458–66. Available from: http://www.ncbi.nlm.nih.gov/pubmed/16049963

22. Gallus S, Foschi R, Talamini R, Altieri A, Negri E, Franceschi S, et al. Risk Factors for Prostate Cancer in Men Aged Less Than 60 Years: A Case-Control Study from Italy. Urology [Internet]. 2007;70(6):1121–6. Available from: http://www.ncbi.nlm.nih.gov/pubmed/18158031

23. Máchová L, Čížek L, Horáková D, Koutná J, Lorenc J, Janoutová G, et al. Association between obesity and cancer incidence in the population of the District Sumperk, Czech Republic. Onkologie. 2007;30(11):538–42.

24. Rodriguez C, Freedland SJ, Deka A, Jacobs EJ, McCullough ML, Patel A V, et al. Body mass index, weight change, and risk of prostate cancer in the Cancer Prevention Study II Nutrition Cohort. Cancer Epidemiol Biomarkers Prev. 2007;16(1):63–9.

25. Wright ME, Chang S-C, Schatzkin A, Albanes D, Kipnis V, Mouw T, et al. Prospective study of adiposity and weight change in relation to prostate cancer incidence and mortality. Cancer [Internet]. 2007;109(4):675–84. Available from: http://www.ncbi.nlm.nih.gov/pubmed/17211863

26. Hernandez BY, Park S-Y, Wilkens LR, Henderson BE, Kolonel LN. Relationship of body mass, height, and weight gain to prostate cancer risk in the multiethnic cohort. Cancer Epidemiol Biomarkers Prev [Internet]. 2009;18(9):2413–21. Available from: http://www.pubmedcentral.nih.gov/articlerender.fcgi?artid=2742565&tool=pmcentrez&rendertype=abstract

27. Wallström P, Bjartell a, Gullberg B, Olsson H, Wirfält E. A prospective Swedish study on body size, body composition, diabetes, and prostate cancer risk. Br J Cancer [Internet]. 2009;100(11):1799–805. Available from: http://www.pubmedcentral.nih.gov/articlerender.fcgi?artid=2695694&tool=pmcentrez&rendertype=abstract

28. Stocks T, Hergens M-P, Englund A, Ye W, Stattin P. Blood pressure, body size and prostate cancer risk in the Swedish Construction Workers cohort. Int J Cancer [Internet]. 2010;127(7):1660–8. Available from: http://www.ncbi.nlm.nih.gov/pubmed/20087861

29. Mori M, Masumori N, Fukuta F, Nagata Y, Sonoda T, Miyanaga N, et al. Weight gain and family history of prostate or breast cancers as risk factors for prostate cancer: results of a case-control study in Japan. Asian Pac J Cancer Prev [Internet]. 2011;12(3):743–7. Available from: http://www.ncbi.nlm.nih.gov/pubmed/21627376

30. Häggström C, Stocks T, Ulmert D, Bjørge T, Ulmer H, Hallmans G, et al. Prospective study on metabolic factors and risk of prostate cancer. Cancer. 2012;118(24):6199–206.

31. Shafique K, McLoone P, Qureshi K, Leung H, Hart C, Morrison DS. Cholesterol and the risk of grade-specific prostate cancer incidence: evidence from two large prospective cohort studies with up to 37 years&apos; follow up. BMC Cancer [Internet]. 2012;12:25. Available from: http://eutils.ncbi.nlm.nih.gov/entrez/eutils/elink.fcgi?dbfrom=pubmed&amp;id=22260413&amp;retmode=ref&amp;cmd=prlinks

32. Møller H, Roswall N, Van Hemelrijck M, Larsen SB, Cuzick J, Holmberg L, et al. Prostate cancer incidence, clinical stage and survival in relation to obesity: A prospective cohort study in Denmark. Int J Cancer [Internet]. 2014;1947:1–8. Available from: http://www.ncbi.nlm.nih.gov/pubmed/25264293

33. Boehm K, Sun M, Larcher A, Blanc-Lapierre A, Schiffmann J, Graefen M, et al. Waist circumference, waist-hip ratio, body mass index, and prostate cancer risk: Results from the North-American case-control study Prostate Cancer & Environment Study. Urol Oncol Semin Orig Investig [Internet]. 2015;33(11):494.e1-494.e7. Available from: http://www.embase.com/search/results?subaction=viewrecord&from=export&id=L605758449%5Cnhttp://dx.doi.org/10.1016/j.urolonc.2015.07.006%5Cnhttp://hz9pj6fe4t.search.serialssolutions.com.proxy.cc.uic.edu/?sid=EMBASE&sid=EMBASE&issn=18732496&id=doi:10.1016%2Fj.ur

34. Choi JB, Moon HW, Park YH, Bae WJ, Cho HJ, Hong SH, et al. The impact of diabetes on the risk of prostate cancer development according to body mass index: A 10-year nationwide cohort study. J Cancer. 2016;7(14):2061–6.

35. Greenland S, Longnecker MP. Methods for trend estimation from summarized dose-response data, with applications to meta-analysis. AmJ Epidemiol. 1992;135(11):1301–9.

36. Orsini N, Bellocco R, Greenland S. Generalized least squares for trend estimation of summarized dose-response data. Vol. 6, Stata Journal. 2006. p. 40–57.

37. Chene G, Thompson SG. Methods for summarizing the risk associations of quantitative variables in epidemiologic studies in a consistent form. Am J Epidemiol [Internet]. 1996;144(6):610–21. Available from: http://aje.oxfordjournals.org/content/144/6/610.abstract

38. Whittemore AS, Kolonel LN, Wu AH, John EM, Gallagher RP, Howe GR, et al. Prostate cancer in relation to diet, physical activity, and body size in blacks, whites, and Asians in the United States and Canada. J Natl Cancer Inst [Internet]. 1995;87(9):652–61. Available from: internal-pdf://72.80.119.144/Whittemore-1995-Prostate cancer in relation to.pdf%5Cnhttp://jnci.oxfordjournals.org/content/87/9/652.full.pdf

39. Lagiou P, Signorello LB, Trichopoulos D, Tzonou a, Trichopoulou a, Mantzoros CS. Leptin in relation to prostate cancer and benign prostatic hyperplasia. Int J Cancer [Internet]. 1998;76(1):25–8. Available from: http://www.ncbi.nlm.nih.gov/pubmed/9533757

40. Heikkila R, Aho K, Heliovaara M, Hakama M, Marniemi J, Reunanen A, et al. Serum testosterone and sex hormone-binding globulin concentrations and the risk of prostate carcinoma: a longitudinal study. Cancer [Internet]. 1999;86(2):312–5. Available from: http://www.ncbi.nlm.nih.gov/entrez/query.fcgi?cmd=Retrieve&db=PubMed&dopt=Citation&list_uids=10421267

41. Hsing AW, Chua S, Gao Y, Gentzschein E, Chang L, Deng J, et al. Prostate Cancer Risk and Serum Levels of Insulin and Leptin : a Population- Based Study. 2001;93(10):783–9.

42. Giles GG, Severi G, English DR, McCredie MRE, MacInnis R, Boyle P, et al. Early growth, adult body size and prostate cancer risk. Int J Cancer. 2003;103(2):241–5.

43. Jian L, Shen ZJ, Lee AH, Binns CW. Moderate physical activity and prostate cancer risk: A case-control study in china. Eur J Epidemiol. 2005;20(2):155–60.

44. Albanes D, Weinstein SJ, Wright ME, Männistö S, Limburg PJ, Snyder K, et al. Serum insulin, glucose, indices of insulin resistance, and risk of prostate cancer. J Natl Cancer Inst. 2009;101(18):1272–9.

45. Farhat GN, Taioli E, Cauley JA, Zmuda JM, Orwoll E, Bauer DC, et al. The association of bone mineral density with prostate cancer risk in the osteoporotic fractures in men (MrOS) study. Cancer Epidemiol Biomarkers Prev. 2009;18(1):148–54.

46. Fowke JH, Motley SS, Concepcion RS, Penson DF, Barocas DA. Obesity, body composition, and prostate cancer. BMC Cancer [Internet]. 2012;12(1):23. Available from: http://bmccancer.biomedcentral.com/articles/10.1186/1471-2407-12-23

47. Yaturu S, Zdunek S, Youngberg B. Vitamin d levels in subjects with prostate cancer compared to age-matched controls. Prostate Cancer [Internet]. 2012;2012:524206. Available from: http://www.pubmedcentral.nih.gov/articlerender.fcgi?artid=3530178&tool=pmcentrez&rendertype=abstract

48. Geybels MS, Verhage BAJ, Arts ICW, Van Schooten FJ, Alexandra Goldbohm R, Van Den Brandt PA. Dietary flavonoid intake, black tea consumption, and risk of overall and advanced stage prostate cancer. Am J Epidemiol. 2013;177(12):1388–98.

49. Bhavsar NA, Bream JH, Meeker AK, Drake CG, Peskoe SB, Dabitao D, et al. A peripheral circulating TH1 cytokine profile is inversely associated with prostate cancer risk in CLUE II. Cancer Epidemiol Biomarkers Prev. 2014;23(11):2561–7.

50. Kunutsor SK, Laukkanen JA. Gamma-glutamyltransferase and risk of prostate cancer: Findings from the KIHD prospective cohort study. Int J Cancer. 2017;140(4):818–24.

51. Borenstein M, Hedges L V., Higgins JPT, Rothstein HR. Introduction to Meta-Analysis [Internet]. Vol. 19, Psychotherapy research journal of the Society for Psychotherapy Research. 2009. 421 p. Available from: http://doi.wiley.com/10.1002/9780470743386

52. Robinson WR, Stevens J, Gammon MD, John EM. Obesity before age 30 years and risk of advanced prostate cancer. Am J Epidemiol [Internet]. 2005;161(12):1107–14. Available from: http://aje.oupjournals.org/cgi/doi/10.1093/aje/kwi150

53. Stark JR, Li H, Kraft P, Kurth T, Giovannucci EL, Stampfer MJ, et al. Circulating prediagnostic interleukin-6 and C-reactive protein and prostate cancer incidence and mortality. Int J Cancer [Internet]. 2009;124(11):2683–9. Available from: http://doi.wiley.com/10.1002/ijc.24241

54. Chia SE, Wong KY, Cheng C, Lau W, Tan PH. Sun exposure and the risk of prostate cancer in the Singapore Prostate Cancer Study: a case-control study. Asian PacJ Cancer Prev [Internet]. 2012;13(7):3179–85. Available from: http://www.ncbi.nlm.nih.gov/pubmed/22994730

55. Hsieh CC, Thanos A, Mitropoulos D, Deliveliotis C, Mantzoros CS, Trichopoulos D. Risk factors for prostate cancer: a case-control study in Greece. Int J Cancer [Internet]. 1999;80(5):699–703. Available from: http://ovidsp.ovid.com/ovidweb.cgi?T=JS&CSC=Y&NEWS=N&PAGE=fulltext&D=med4&AN=10048970

56. Stefani E De, Boffetta PL, Ronco A, Deneo-Pellegrini H. Meat Consumption, Related Nutrients, Obesity and Risk of Prostate Cancer: a Case-Control Study in Uruguay. Asian Pac J Cancer Prev. 2016;17(4):1937–45.

57. Brändstedt J, Almquist M, Manjer J, Malm J. Vitamin D, PTH, and calcium and the risk of prostate cancer: a prospective nested case-control study. Cancer Causes Control [Internet]. 2012;23(8):1377–85. Available from: http://www.ncbi.nlm.nih.gov/pubmed/22706676

58. Andersson SO, Baron J, Bergström R, Lindgren C, Wolk a, Adami HO. Lifestyle factors and prostate cancer risk: a case-control study in Sweden. Cancer Epidemiol Biomarkers Prev. 1996;5(7):509–13.

59. Schuurman AG, Goldbohm RA, Dorant E, van den Brandt PA. Anthropometry in relation to prostate cancer risk in the Netherlands Cohort Study. Am J Epidemiol [Internet]. 2000;151(6):541–9. Available from: http://www.ncbi.nlm.nih.gov/entrez/query.fcgi?cmd=Retrieve&db=PubMed&dopt=Citation&list_uids=10733035

60. Cui Y, Winton MI, Zhang ZF, Rainey C, Marshall J, De Kernion JB, et al. Dietary boron intake and prostate cancer risk. Oncol Rep. 2004;11(4):887–92.

61. Håheim LL, Wisløff TF, Holme I, Nafstad P. Metabolic syndrome predicts prostate cancer in a cohort of middle-aged Norwegian men followed for 27 years. Am J Epidemiol. 2006;164(8):769–74.

62. Lundqvist E, Kaprio J, Verkasalo PK, Pukkala E, Koskenvuo M, Söderberg KC, et al. Co-twin control and cohort analyses of body mass index and height in relation to breast, prostate, ovarian, corpus uteri, colon and rectal cancer among Swedish and Finnish twins. Int J Cancer [Internet]. 2007;121(4):810–8. Available from: http://www.ncbi.nlm.nih.gov/pubmed/17455257

63. Chamie K, DeVere White RW, Lee D, Ok J-H, Ellison LM. Agent Orange exposure, Vietnam War veterans, and the risk of prostate cancer. Cancer [Internet]. 2008;113(9):2464–70. Available from: http://www.ncbi.nlm.nih.gov/pubmed/18666213

64. Burton A, Martin R, Galobardes B, Davey Smith G, Jeffreys M. Young adulthood body mass index and risk of cancer in later adulthood: historical cohort study. Cancer Causes Control. 2010;21(12):2069–77.

65. Discacciati A, Orsini N, Andersson S-O, Andrén O, Johansson J-E, Wolk A. Body mass index in early and middle-late adulthood and risk of localised, advanced and fatal prostate cancer: a population-based prospective study. Br J Cancer [Internet]. 2011;105(7):1061–8. Available from: http://dx.doi.org/10.1038/bjc.2011.319

66. Bassett JK, Severi G, Baglietto L, MacInnis RJ, Hoang HN, Hopper JL, et al. Weight change and prostate cancer incidence and mortality. Int J Cancer. 2012;131(7):1711–9.

67. pinnacle, Nemesure B, Wu S-Y-., Hennis A, Leske MC. Central Adiposity and Prostate Cancer in a Black Population. Cancer Epidemiol Biomarkers Prev [Internet]. 2012;21(5):851–8. Available from: http://www.ncbi.nlm.nih.gov/pubmed/22402288%5Cnhttp://cebp.aacrjournals.org/cgi/doi/10.1158/1055-9965.EPI-12-0071

68. Möller E, Adami H-O, Mucci L a, Lundholm C, Bellocco R, Johansson J-E, et al. Lifetime body size and prostate cancer risk in a population-based case-control study in Sweden. Cancer Causes Control [Internet]. 2013;24(12):2143–55. Available from: http://www.ncbi.nlm.nih.gov/pubmed/24048969

69. Rao GA, Mann JR, Bottai M, Uemura H, Burch JB, Bennett CL, et al. Angiotensin receptor blockers and risk of prostate cancer among united states veterans. J Clin Pharmacol. 2013;53(7):773–8.

70. Salem S, Hosseini M, Allameh F, Babakoohi S, Mehrsai A, Pourmand G. Serum calcium concentration and prostate cancer risk: a multicenter study. Nutr Cancer [Internet]. 2013;65(7):961–8. Available from: http://www.tandfonline.com/doi/abs/10.1080/01635581.2013.806936#.VuRf8fkrLIU

71. Bhaskaran K, Douglas I, Forbes H, dos-Santos-Silva I, Leon DA, Smeeth L. Body-mass index and risk of 22 specific cancers: a population-based cohort study of 5·24 million UK adults. Lancet [Internet]. 2014;384(9945):755–65. Available from: http://dx.doi.org/10.1016/S0140-6736(14)60892-8

72. Perez-Cornago A, Appleby PN, Pischon T, Tsilidis KK, Tjønneland A, Olsen A, et al. Tall height and obesity are associated with an increased risk of aggressive prostate cancer: Results from the EPIC cohort study. BMC Med. 2017;15(1).

73. Le Marchand L, Kolonel LN, Wilkens LR, Myers BC, Hirohata T. Animal fat consumption and prostate cancer: a prospective study in Hawaii. Epidemiology [Internet]. 1994;5(3):276–82. Available from: http://www.ncbi.nlm.nih.gov/pubmed/8038241

74. Veierød MB, Laake P, Thelle DS. Dietary fat intake and risk of prostate cancer: a prospective study of 25,708 Norwegian men. Int J Cancer [Internet]. 1997;73(5):634–8. Available from: http://www.ncbi.nlm.nih.gov/pubmed/9398038

75. Habel LA, Van Den Eeden SK, Friedman GD. Body size, age at shaving initiation, and prostate cancer in a large, multiracial cohort. Prostate [Internet]. 2000;43(2):136–43. Available from: http://www.ncbi.nlm.nih.gov/pubmed/10754529

76. Hsing a W, Deng J, Sesterhenn I a, Mostofi FK, Stanczyk FZ, Benichou J, et al. Body size and prostate cancer: a population-based case-control study in China. Cancer Epidemiol Biomarkers Prev. 2000;9(12):1335–41.

77. Putnam SD, Cerhan JR, Parker AS, Bianchi GD, Wallace RB, Cantor KP, et al. Lifestyle and anthropometric risk factors for prostate cancer in a cohort of Iowa men. Ann Epidemiol. 2000;10(6):361–9.

78. Pan SY, Johnson KC, Ugnat AM, Wen SW, Mao Y. Association of Obesity and Cancer Risk in Canada. Am J Epidemiol. 2004;159(3):259–68.

79. Cox B, Sneyd MJ, Paul C, Skegg DCG. Risk factors for prostate cancer: A national case-control study. Int J Cancer [Internet]. 2006;119(7):1690–4. Available from: http://doi.wiley.com/10.1002/ijc.22022

80. Littman AJ, White E, Kristal AR. Anthropometrics and prostate cancer risk. Am J Epidemiol [Internet]. 2007;165(11):1271–9. Available from: http://www.ncbi.nlm.nih.gov/pubmed/17395597

81. Attner B, Landin-Olsson M, Lithman T, Noreen D, Olsson H. Cancer among patients with diabetes, obesity and abnormal blood lipids: a population-based register study in Sweden. Cancer Causes Control [Internet]. 2012;23(5):769–77. Available from: http://link.springer.com/article/10.1007%2Fs10552-012-9946-5%5Cnhttp://download.springer.com/static/pdf/443/art%253A10.1007%252Fs10552-012-9946-5.pdf?originUrl=http%3A%2F%2Flink.springer.com%2Farticle%2F10.1007%2Fs10552-012-9946-5&token2=exp=1455626637~acl=

82. Harding JL, Shaw JE, Anstey KJ, Adams R, Balkau B, Brennan-Olsen SL, et al. Comparison of anthropometric measures as predictors of cancer incidence: A pooled collaborative analysis of 11 Australian cohorts. Int J Cancer. 2015;137(7):1699–708.

83. Heir T, Falk RS, Robsahm TE, Sandvik L, Erikssen J, Tretli S. Cholesterol and prostate cancer risk: A long-term prospective cohort study. BMC Cancer. 2016;16(1).

84. Baillargeon J, Pollock BH, Kristal AR, Bradshaw P, Hernandez J, Basler J, et al. The association of body mass index and prostate-specific antigen in a population-based study. Cancer. 2005;103(5):1092–5.

85. Freedland SJ, Platz EA, Presti JC, Aronson WJ, Amling CL, Kane CJ, et al. Obesity, serum prostate specific antigen and prostate size: Implications for prostate cancer detection. J Urol. 2006;175(2):500–4.

86. J.C. S, M.S. L, H.S. C, C.H. P. The association of body mass index and prostate-specific antigen [Internet]. Vol. 48, Korean Journal of Urology. 2007. p. 1121–4. Available from: http://ovidsp.ovid.com/ovidweb.cgi?T=JS&PAGE=reference&D=emed11&NEWS=N&AN=350200835

87. Bañez LL, Hamilton RJ, Partin AW, Vollmer RT, Sun L, Rodriguez C, et al. Obesity-related plasma hemodilution and PSA concentration among men with prostate cancer. Jama [Internet]. 2007;298(19):2275–80. Available from: http://jama.jamanetwork.com/article.aspx?articleid=209508%5Cnhttp://jama.jamanetwork.com/article.aspx?doi=10.1001/jama.298.19.2275%5Cnhttp://www.ncbi.nlm.nih.gov/pubmed/18029831

88. Price MM, Hamilton RJ, Robertson CN, Butts MC, Freedland SJ. Body Mass Index, Prostate-Specific Antigen, and Digital Rectal Examination Findings Among Participants in a Prostate Cancer Screening Clinic. Urology [Internet]. 2008;71(5):787–91. Available from: http://www.ncbi.nlm.nih.gov/pubmed/18267334

89. Ando R, Nagaya T, Hashimoto Y, Suzuki S, Itoh Y, Umemoto Y, et al. Inverse relationship between obesity and serum prostate-specific antigen level in healthy Japanese men: a hospital-based cross-sectional survey, 2004-2006. Urology. 2008;72(3):561–5.

90. Park J-H, Cho B-L, Kwon H-T, Lee C-M, Han H-J. Effect of body mass index and waist circumference on prostate specific antigen and prostate volume in a generally healthy Korean population. J Urol [Internet]. 2009;182(1):106-10-1. Available from: http://www.ncbi.nlm.nih.gov/pubmed/19450837

91. Muller H, Raum E, Rothenbacher D, Stegmaier C, Brenner H, Mu H. Association of Diabetes and Body Mass Index with Levels of Prostate-Specific Antigen: Implications for Correction of Prostate-Specific Antigen Cutoff Values? Cancer Epidemiol Biomarkers Prev [Internet]. 2009;18(5):1350–6. Available from: http://cebp.aacrjournals.org/cgi/content/abstract/18/5/1350

92. Wright JL, Lin DW, Stanford JL. The effect of demographic and clinical factors on the relationship between BMI and PSA levels. Prostate. 2011;71(15):1631–7.

93. Kim JM, Song PH, Kim HT, Moon KH. Effect of obesity on prostate-specific antigen, prostate volume, and international prostate symptom score in patients with benign prostatic hyperplasia. Korean J Urol. 2011;52(6):401–5.

94. Park S-G, Choi H-C, Cho B, Kwon Y-M, Kwon H-T, Park J-H. Effect of central obesity on prostate specific antigen measured by computerized tomography: related markers and prostate volume. J Urol [Internet]. 2012;187(5):1589–93. Available from: http://www.ncbi.nlm.nih.gov/pubmed/22425083

95. Bhindi B, Margel D, Trottier G, Hamilton RJ, Kulkarni GS, Hersey KM, et al. Obesity is associated with larger prostate volume but not with worse urinary symptoms: Analysis of a large multiethnic cohort. Urology [Internet]. 2014;83(1):81–7. Available from: http://dx.doi.org/10.1016/j.urology.2013.07.039

96. Waters K, Henderson B, Stram D, Wan P, Kolonel L, Haiman C. Association of Diabetes With Prostate Cancer Risk in the Multiethnic Cohort. Am J Epidemiol [Internet]. 2009; Available from: http://aje.oxfordjournals.org/cgi/content/full/kwp003v1%5Cnpapers2://publication/doi/10.1093/aje/kwp003

97. Higgins JPT, White IR, Anzures-Cabrera J. Meta-analysis of skewed data: Combining results reported on log-transformed or raw scales. Stat Med. 2008;27(29):6072–92.

98. Higgins JPT, Green S. Cochrane Handbook for Systematic Reviews of Interventions Version 5.1.0 [updated March 2011]. In: The Cochrane Collaboration [Internet]. 2011. Available from: www.handbook.cochrane.org

99. Harrison S. Examining the role of mediation in the associations of individual characteristics with prostate specific antigen (PSA) and prostate cancer risk. University of Bristol; 2018.

100. Li J, Thompson T, Joseph DA, Master VA. Association Between Smoking Status, and Free, Total and Percent Free Prostate Specific Antigen. J Urol [Internet]. 2012;187(4):1228–33. Available from: http://linkinghub.elsevier.com/retrieve/pii/S0022534711057648

101. Chamie K, Oberfoell S, Kwan L, Labo J, Wei JT, Litwin MS. Body mass index and prostate cancer severity: Do obese men harbor more aggressive disease on prostate biopsy? Urology [Internet]. 2013;81(5):949–55. Available from: http://dx.doi.org/10.1016/j.urology.2013.01.021

102. Bonn SE, Sjölander A, Tillander A, Wiklund F, Grönberg H, Bälter K. Body mass index in relation to serum prostate-specific antigen levels and prostate cancer risk. Int J Cancer. 2016;139(1):50–7.

103. Gray MA, Delahunt B, Fowles JR, Weinstein P, Cooke RR, Nacey JN. Demographic and clinical factors as determinants of serum levels of prostate specific antigen and its derivatives. Anticancer Res. 2004;24(3 B):2069–72.

104. Chang IH, Han JH, Ahn SH. Association of obesity with prostate specific antigen and prostate specific antigen velocity in healthy young men. J Urol [Internet]. 2008;179(3):881–6. Available from: http://www.ncbi.nlm.nih.gov/entrez/query.fcgi?cmd=Retrieve&db=PubMed&dopt=Citation&list_uids=18207169

105. Chia S-E, Lau WKO, Chin CM, Tan J, Ho SH, Lee J, et al. Effect of ageing and body mass index on prostate-specific antigen levels among Chinese men in Singapore from a community-based study. BJU Int [Internet]. 2009;103(11):1487–91. Available from: http://www.ncbi.nlm.nih.gov/pubmed/19076145

106. Chiu PK-F, Wong AY-F, Hou S-M, Yip SK-H, Ng C-F. Effect of body mass index on serum prostate-specific antigen levels among patients presenting with lower urinary tract symptoms. Asian Pac J Cancer Prev. 2011;12(8):1937–40.

107. Liu M, Wang J-Y, Zhu L, Wan G. Body mass index and serum lipid profile influence serum prostate-specific antigen in Chinese men younger than 50 years of age. Asian J Androl [Internet]. 2011;13(4):640–3. Available from: http://www.asiaandro.com/Abstract.asp?doi=10.1038/aja.2010.104

108. Wallner LP, Morgenstern H, McGree ME, Jacobson DJ, St. Sauver JL, Jacobsen SJ, et al. The Effects of Body Mass Index on Changes in Prostate-Specific Antigen Levels and Prostate Volume Over 15 Years of Follow-up: Implications for Prostate Cancer Detection. Cancer Epidemiol Biomarkers Prev. 2011;20(3):501–8.

109. Pater LE, Hart KW, Blonigen BJ, Lindsell CJ, Barrett WL. Relationship Between Prostate-specific Antigen, Age, and Body Mass Index in a Prostate Cancer Screening Population. Am J Clin Oncol [Internet]. 2012;35(5):490–2. Available from: http://content.wkhealth.com/linkback/openurl?sid=WKPTLP:landingpage&an=00000421-201210000-00015

110. Gómez-Guerra LS, Hernández-Torres AU, Blanco-Guzmán A, Solís-Rodríguez DE, Ortiz-Lara GE, Cortés-González JR. Effect of body mass index on PSA in northeast Mexican patients. Actas Urológicas Españolas (English Ed [Internet]. 2012;36(5):302–5. Available from: http://www.sciencedirect.com/science/article/pii/S2173578612001126

111. Ikuerowo SO, Omisanjo OA, Bioku MJ, Ajala MO, Esho JO. Effect of obesity on serum prostate-specific antigen in nigerian men. Urol Int. 2012;89(1):52–6.

112. Yang WJ. The likelihood of having a serum PSA level of ≥2.5 or ≥4.0 ng ml(-1) according to obesity in a screened Korean population. Asian J Androl [Internet]. 2013;15(6):770–2. Available from: http://www.scopus.com/inward/record.url?eid=2-s2.0-84887242982&partnerID=tZOtx3y1

113. Taghavi R, Aameli M, Jahed-Ataeian S, Hasanzade J. Relationship between body mass index and prostate specific antigen in patient with lower urinary tract symptoms. Urology [Internet]. 2014;84(4):S318–9. Available from: http://www.embase.com/search/results?subaction=viewrecord&from=export&id=L71654647

114. Adegun PT, Adebayo PB, Atiba SA. The likelihood of having serum level of PSA of ≥4.0 ng/mL and ≥10.0 ng/mL in non-obese and obese Nigerian men with LUTS. Asian J Urol. 2015;2(3):158–62.

115. Zhang J, Sheng B, Ma M, Nan X. An inverse association of obesity and prostate-specific antigen in elderly males. Int J Clin Exp Med. 2016;9(9):18746–53.

116. Yun J, Lee H, Yang W. Association between systemic inflammation and serum prostate-specific antigen in a healthy Korean population. Turkish J Urol. 2017;43(3):284–8.

117. Blanker MH, Groeneveld FPMJ, Prins A, Bernsen RMD, Bohnen AM, Bosch JLHR. Strong effects of definition and nonresponse bias on prevalence rates of clinical benign prostatic hyperplasia: The Krimpen study of male urogenital tract problems and general health status. BJU Int. 2000;85(6):665–71.

118. Thompson IM, Goodman PJ, Tangen CM, Parnes HL, Minasian LM, Godley PA, et al. Long-term survival of participants in the prostate cancer prevention trial. N Engl J Med [Internet]. 2013;369(7):603–10. Available from: http://www.nejm.org/doi/full/10.1056/NEJMoa1215932#t=article

119. Hughes RA, Heron J, Sterne JAC, Tilling K. Accounting for missing data in statistical analyses: multiple imputation is not always the answer. Int J Epidemiol. 2019;

120. Andriole GL, Crawford ED, Grubb 3rd RL, Buys SS, Chia D, Church TR, et al. Prostate cancer screening in the randomized Prostate, Lung, Colorectal, and Ovarian Cancer Screening Trial: mortality results after 13 years of follow-up. J Natl Cancer Inst [Internet]. 2012;104(2):125–32. Available from: http://www.ncbi.nlm.nih.gov/pubmed/22228146%5Cnhttp://jnci.oxfordjournals.org/content/104/2/125.full.pdf

121. Lane JA, Donovan JL, Davis M, Walsh E, Dedman D, Down L, et al. Active monitoring, radical prostatectomy, or radiotherapy for localised prostate cancer: Study design and diagnostic and baseline results of the ProtecT randomised phase 3 trial. Lancet Oncol. 2014;15(10):1109–18.

122. Eisenberg ML, Davies BJ, Cooperberg MR, Cowan JE, Carroll PR. Prognostic Implications of an Undetectable Ultrasensitive Prostate-Specific Antigen Level after Radical Prostatectomy. Eur Urol. 2010;57(4):622–30.

123. Moul JW. The evolving definition of advanced prostate cancer. Rev Urol [Internet]. 2004;6 Suppl 8(Suppl 8):S10-7. Available from: http://www.ncbi.nlm.nih.gov/pubmed/16985915%5Cnhttp://www.pubmedcentral.nih.gov/articlerender.fcgi?artid=PMC1472896

124. StataCorp. Stata multiple-imputation reference manual. College Station, TX: StataCorp LP; 2013.

125. Bell KJL, Del Mar C, Wright G, Dickinson J, Glasziou P. Prevalence of incidental prostate cancer: A systematic review of autopsy studies. Int J Cancer. 2015;137(7):1749–57.
