# Supplementary material for: Systematic review and meta-analysis of the associations between body mass index, prostate cancer, advanced prostate cancer, and prostate-specific antigen
Source: Cancer Causes Control. 2020 Mar 11;31(5):431–49. doi: 10.1007/s10552-020-01291-3 (PMC7105428; doi:10.1007/s10552-020-01291-3)
Supplement: Supplementary file 2 — Supplementary file2 (DOCX 1402 kb) [file 10552_2020_1291_MOESM2_ESM.docx]

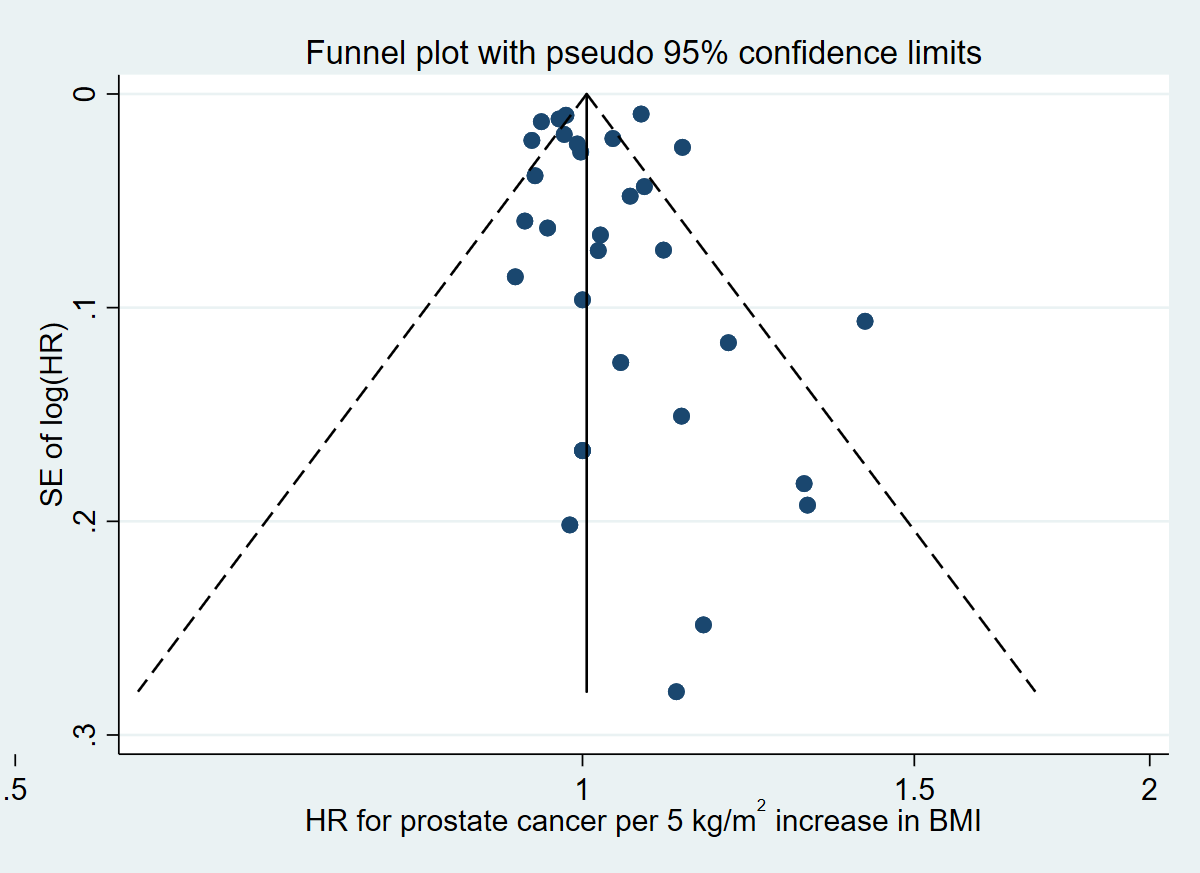


Figure S1 Funnel plot for the association between BMI and prostate cancer (hazard ratios)


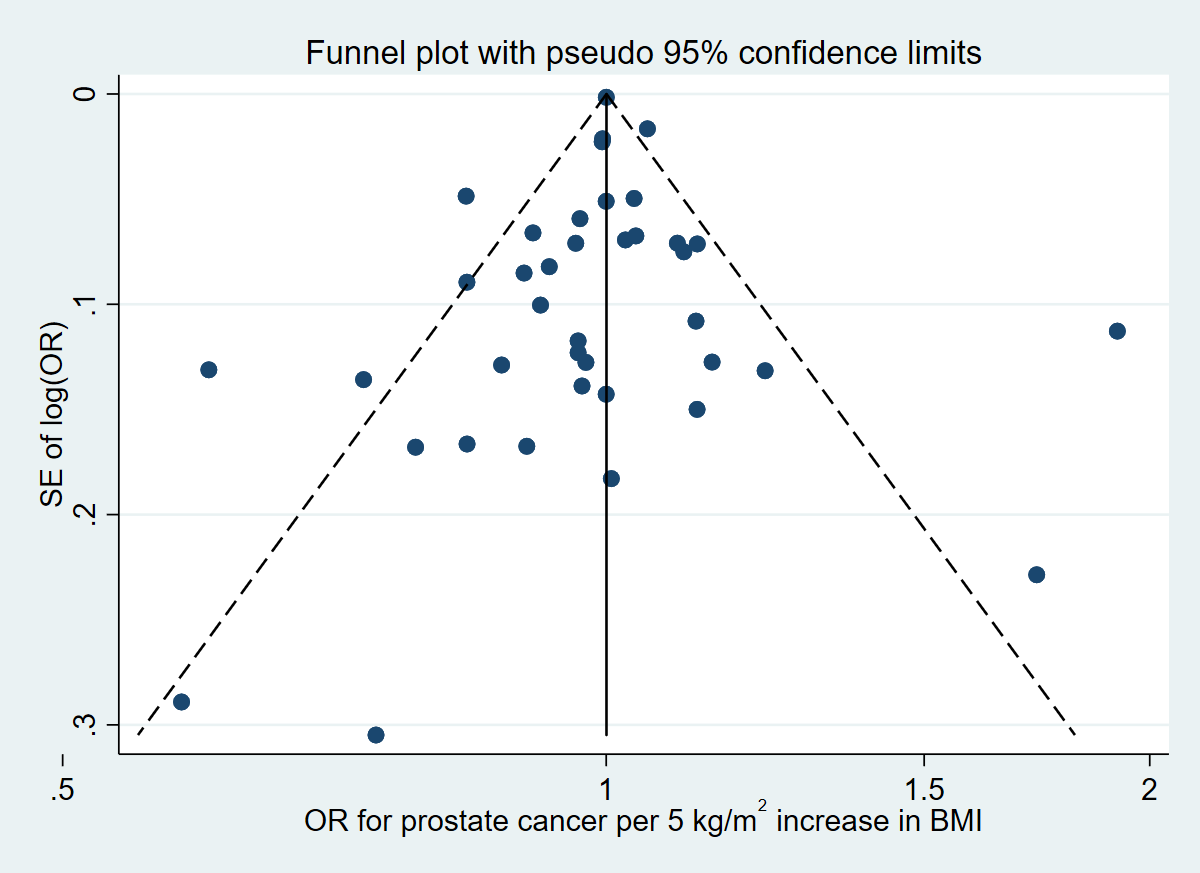


Figure S2 Funnel plot for the association between BMI and prostate cancer (odds ratios)


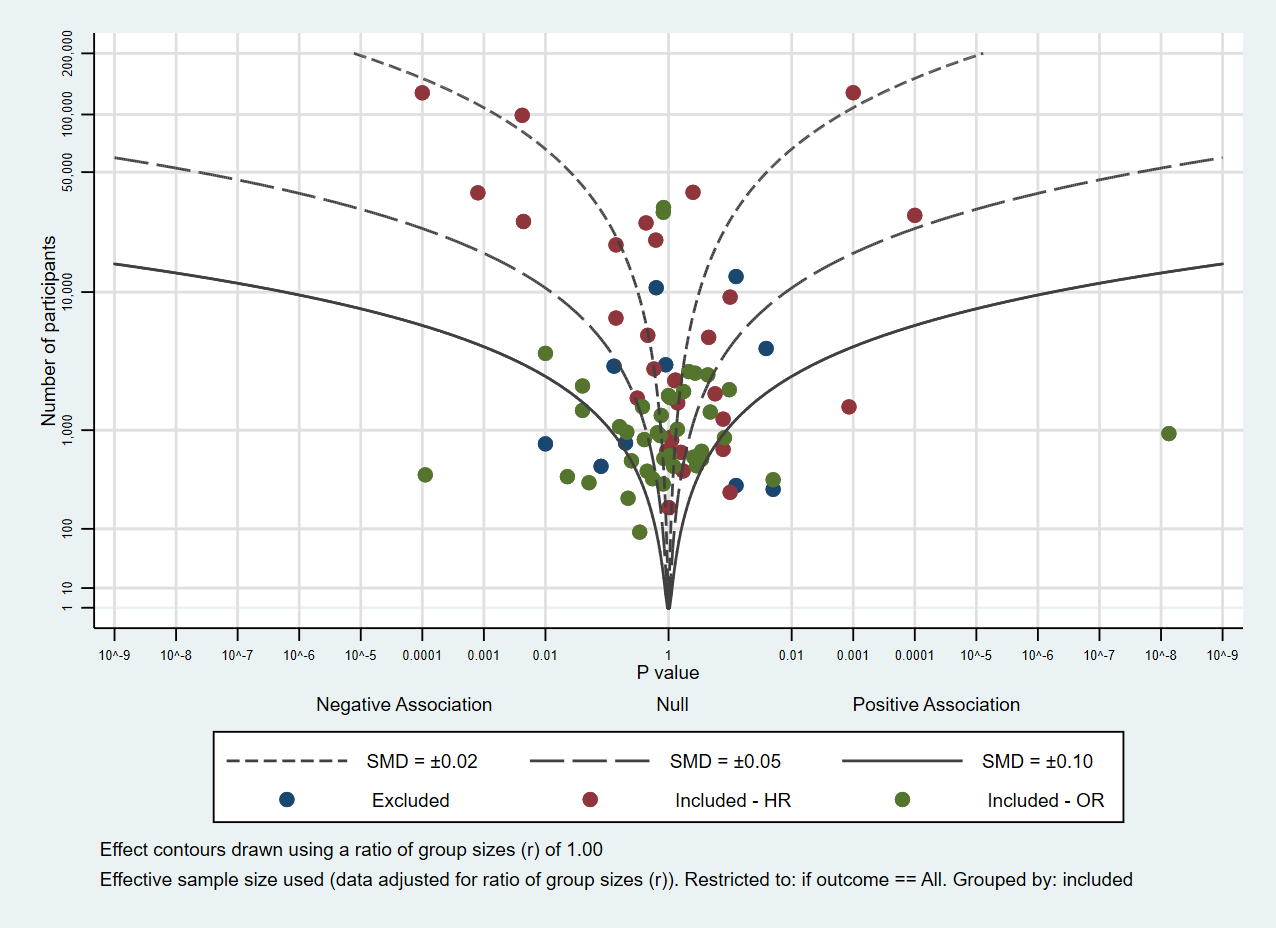


Figure S3 Albatross plot for the association between BMI and prostate cancer


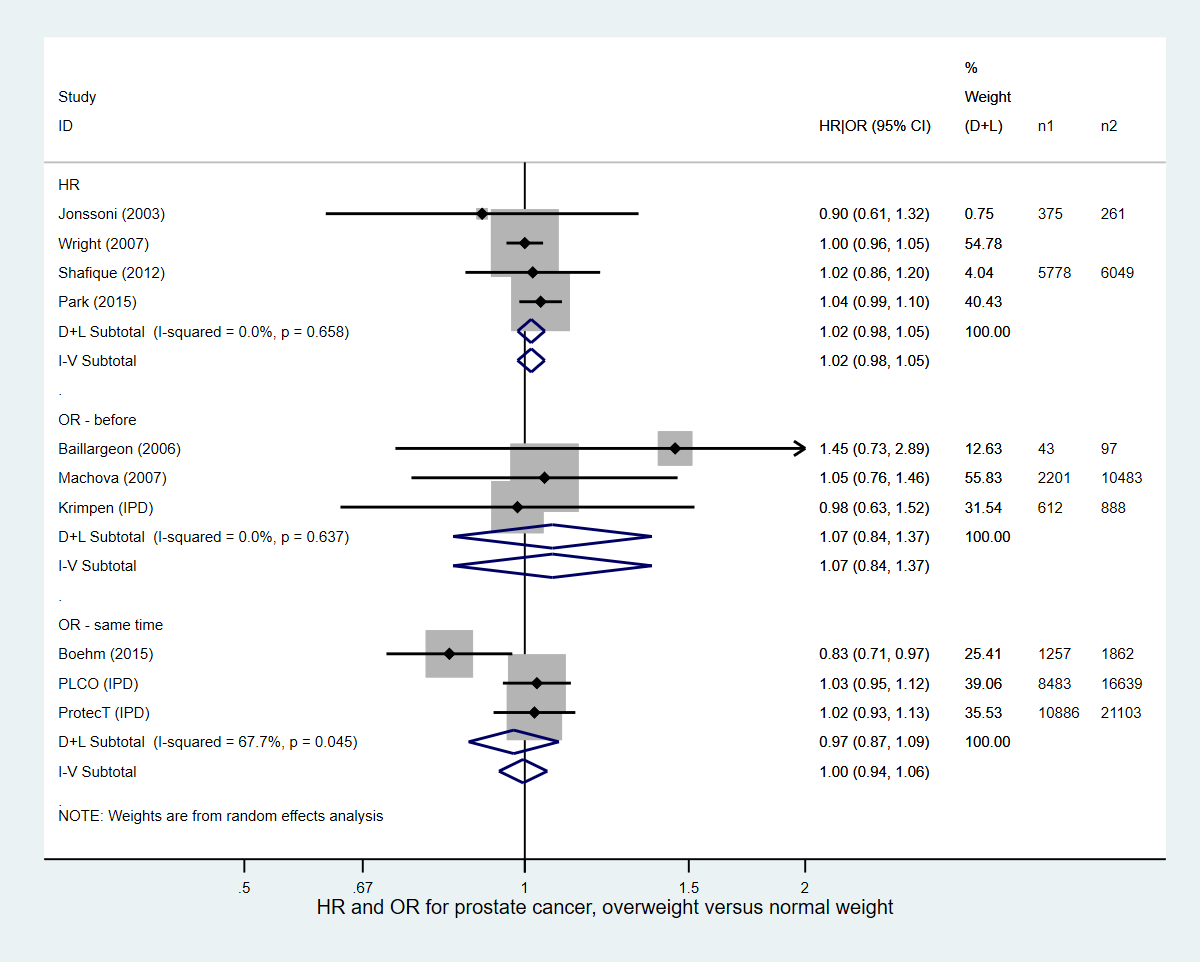


Figure S4 Forest plot of the HRs and ORs for prostate cancer for overweight versus normal weight BMI categories, n1 = number of normal weight participants, n2 = number of overweight participants, blanks indicate missing data


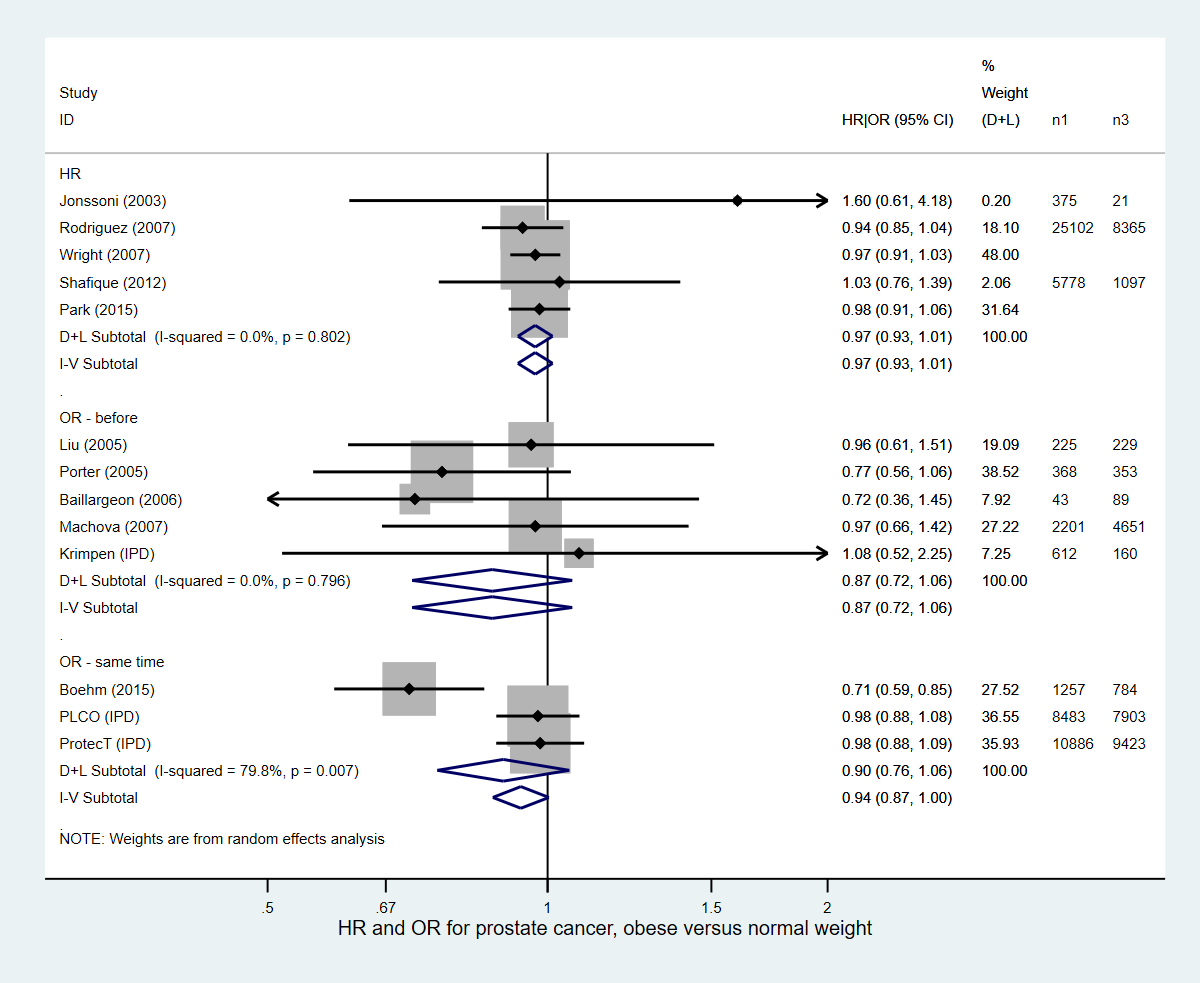


Figure S5 Forest plot of the HRs and ORs for prostate cancer for obese versus normal BMI categories, n1 = number of normal weight participants, n3 = number of obese participants, blanks indicate missing data


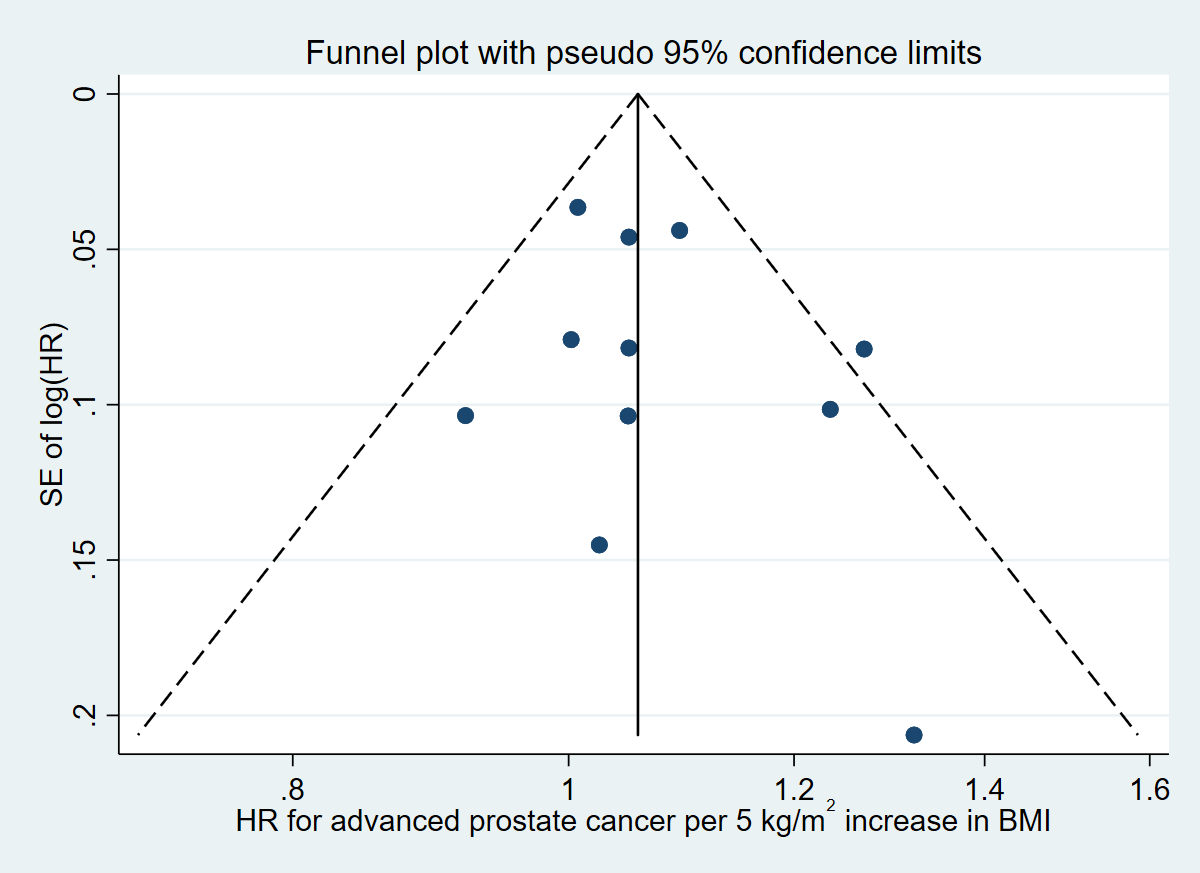


Figure S6 Funnel plot for the association between BMI and advanced prostate cancer (hazard ratios)


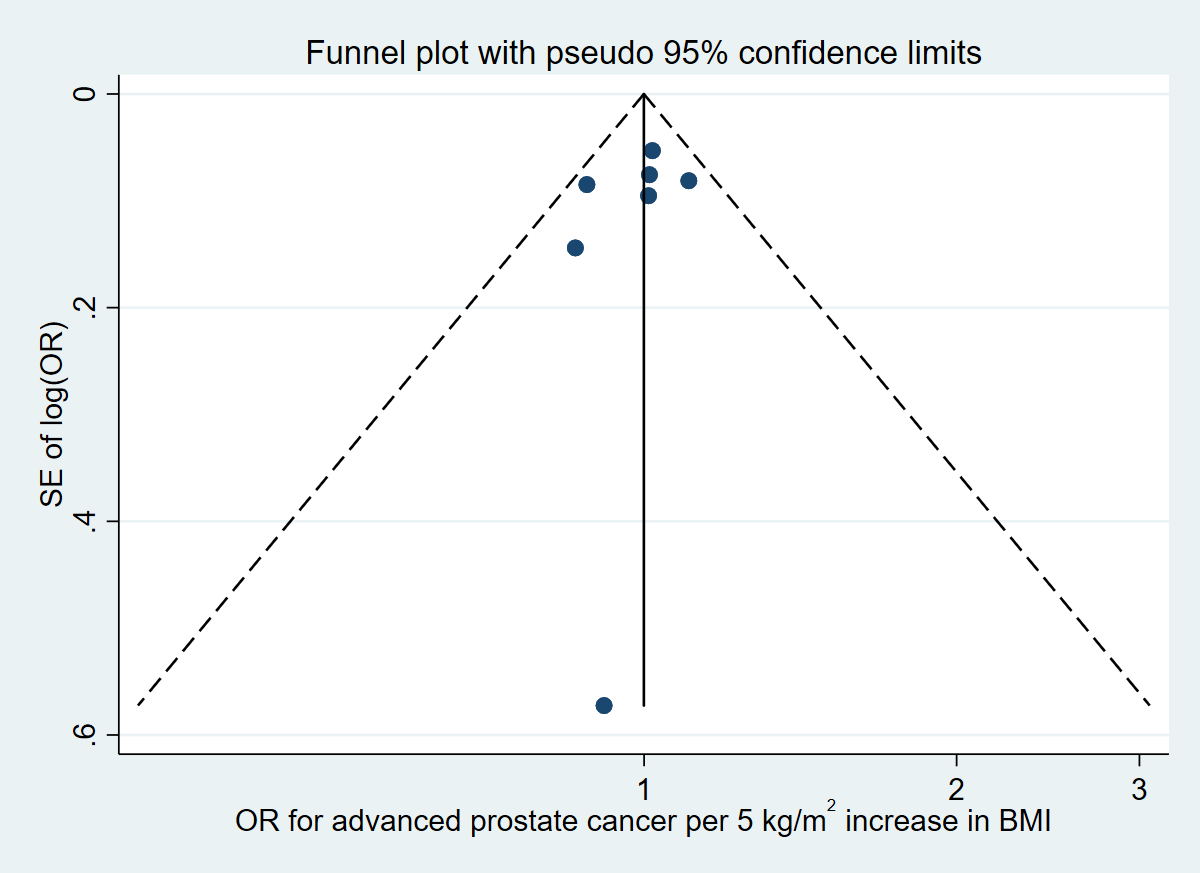


Figure S7 Funnel plot for the association between BMI and advanced prostate cancer (odds ratios)


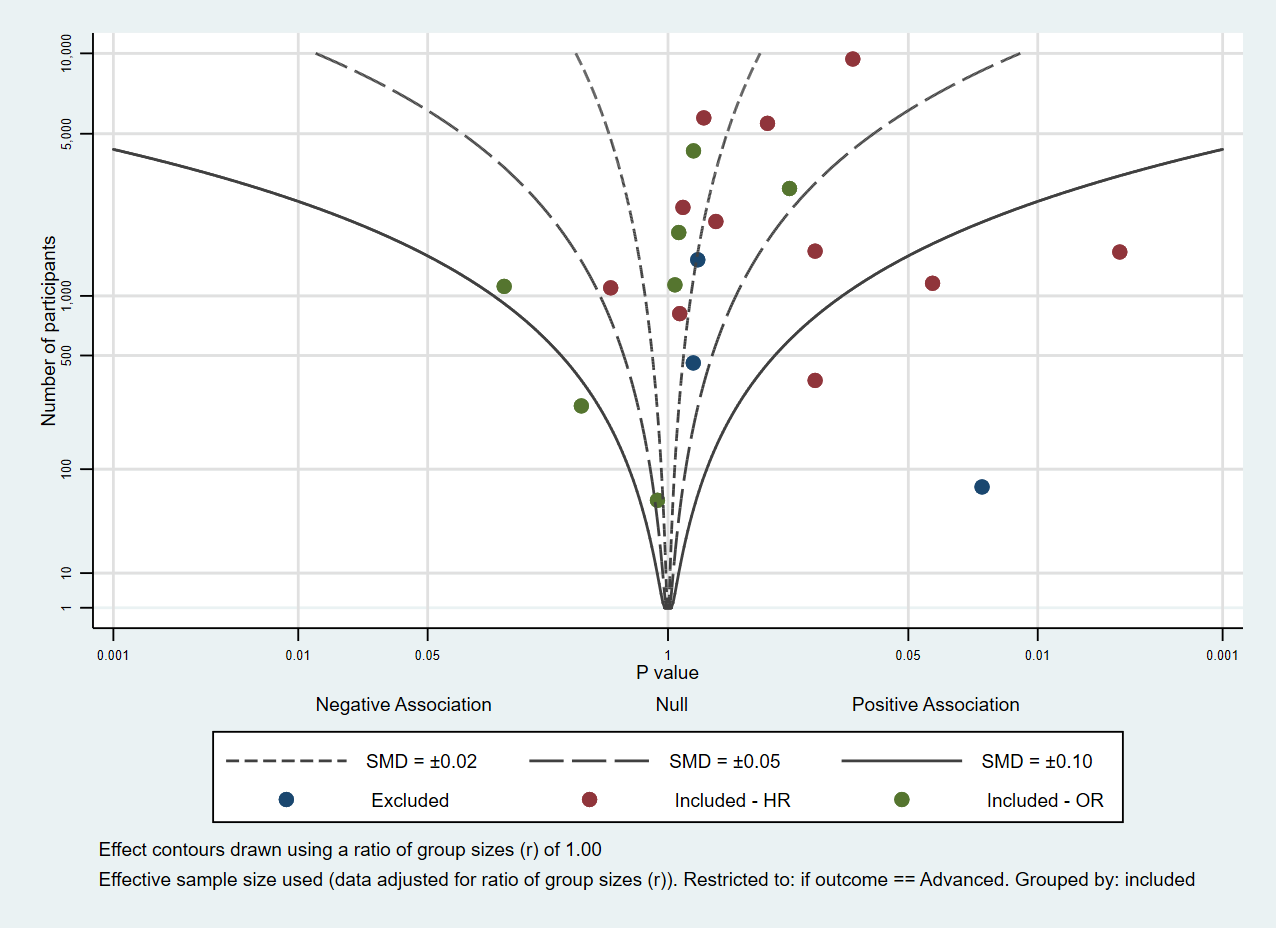


Figure S8 Albatross plot for the association between BMI and advanced prostate cancer


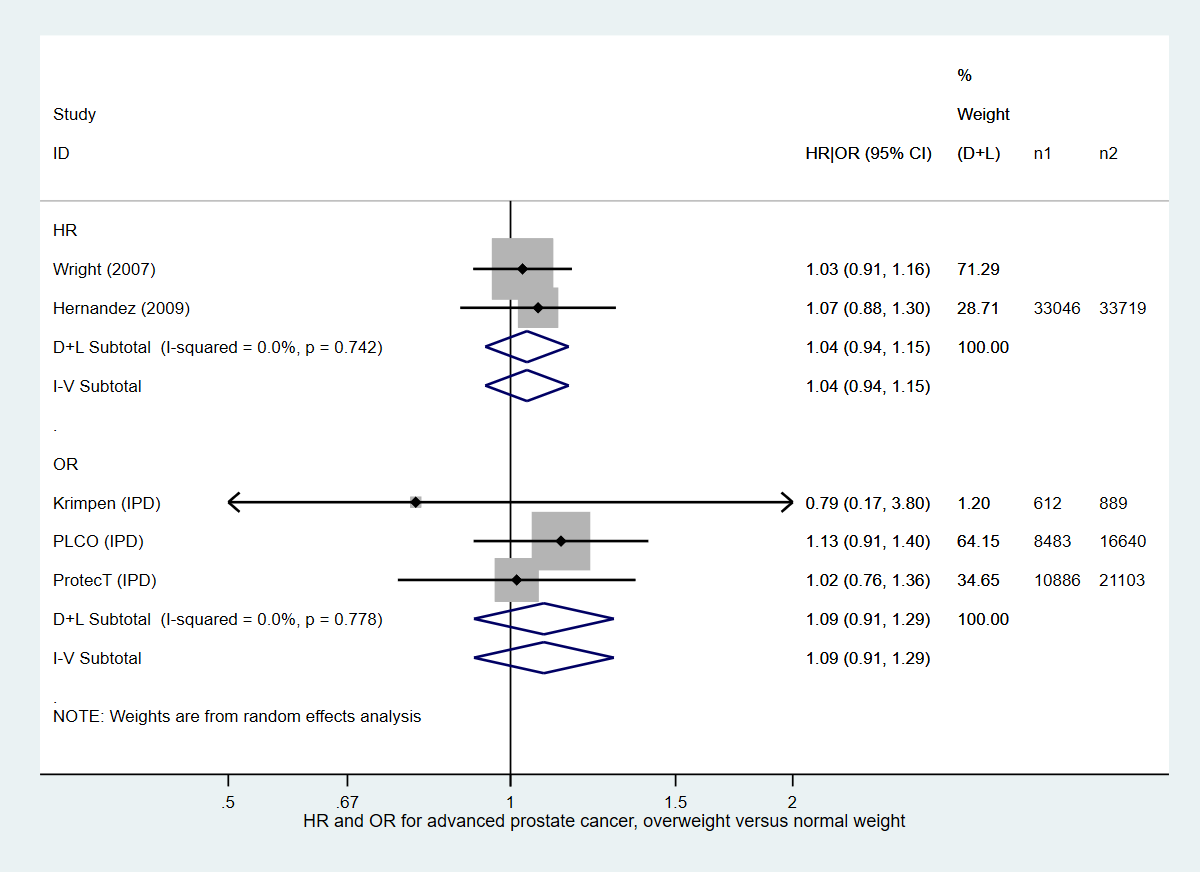


Figure S9 Forest plot of the HRs and ORs for advanced prostate cancer for overweight versus normal weight BMI categories, n1 = number of normal weight participants, n2 = number of overweight participants, blanks indicate missing data. ORs (before and same time) were combined as the Krimpen study was the only study measuring BMI before the outcome and presenting an OR


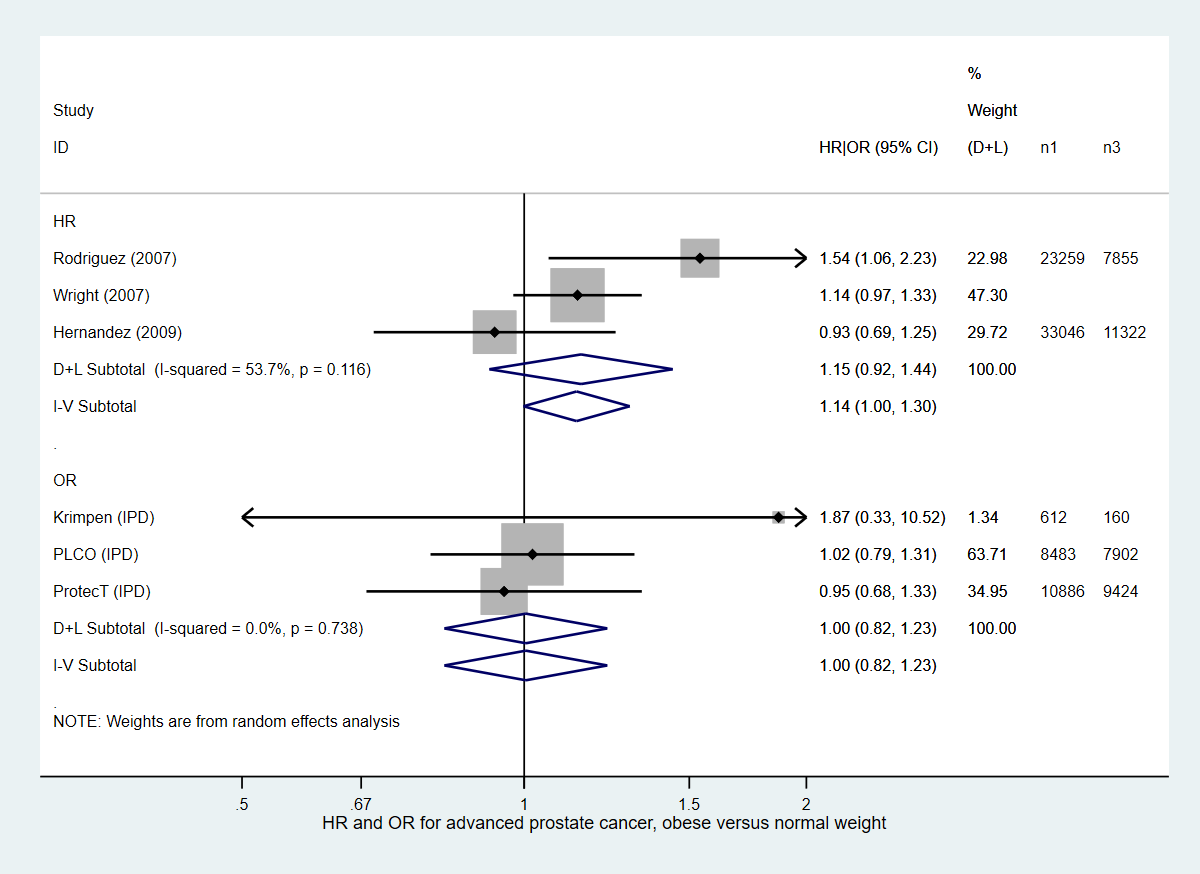


Figure S10 Forest plot of the HRs and ORs for advanced prostate cancer for obese versus normal weight BMI categories, n1 = number of normal weight participants, n3 = number of obese participants, blanks indicate missing data. ORs (before and same time) were combined as the Krimpen study was the only study measuring BMI before the outcome and presenting an OR


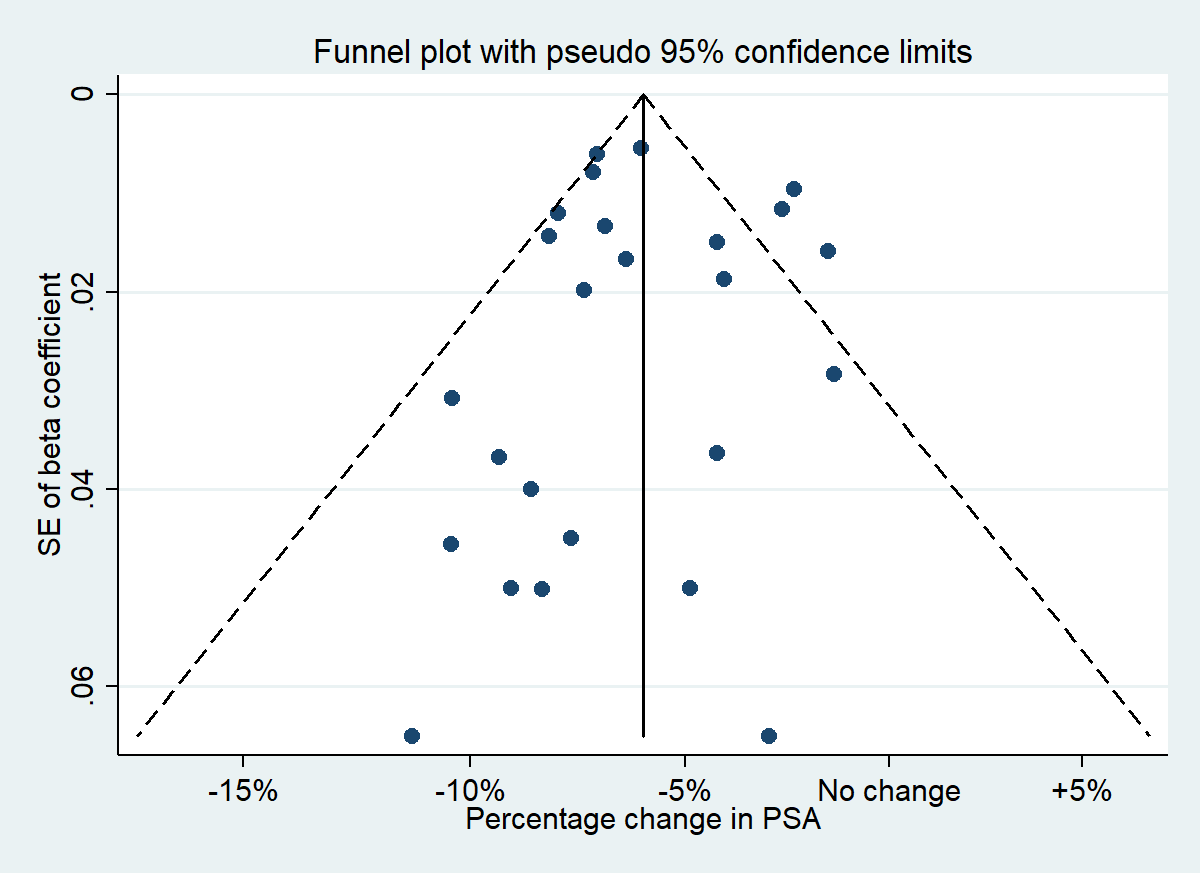


Figure S11 Funnel plot for the association between BMI and PSA


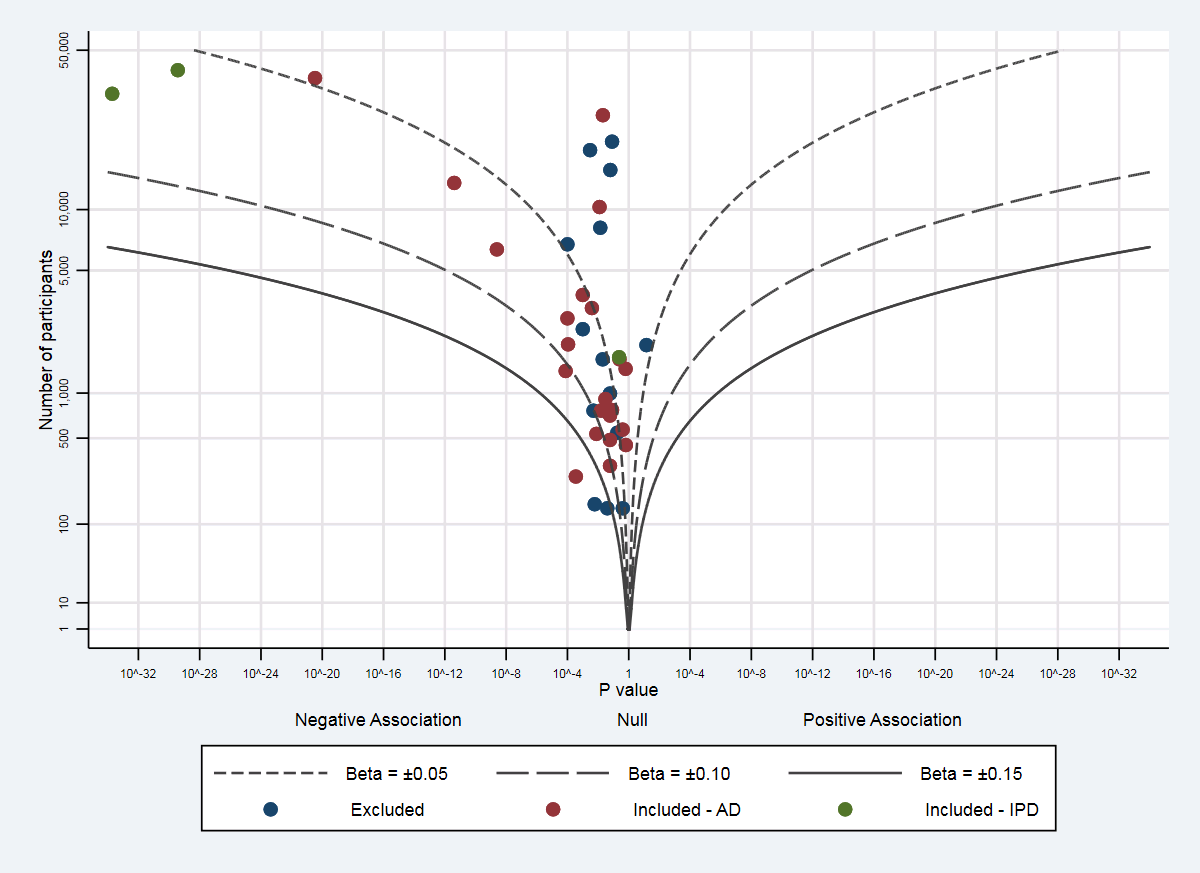


Figure S12 Albatross plot for the association between BMI and log-PSA. AD = aggregate data, IPD = individual participant data


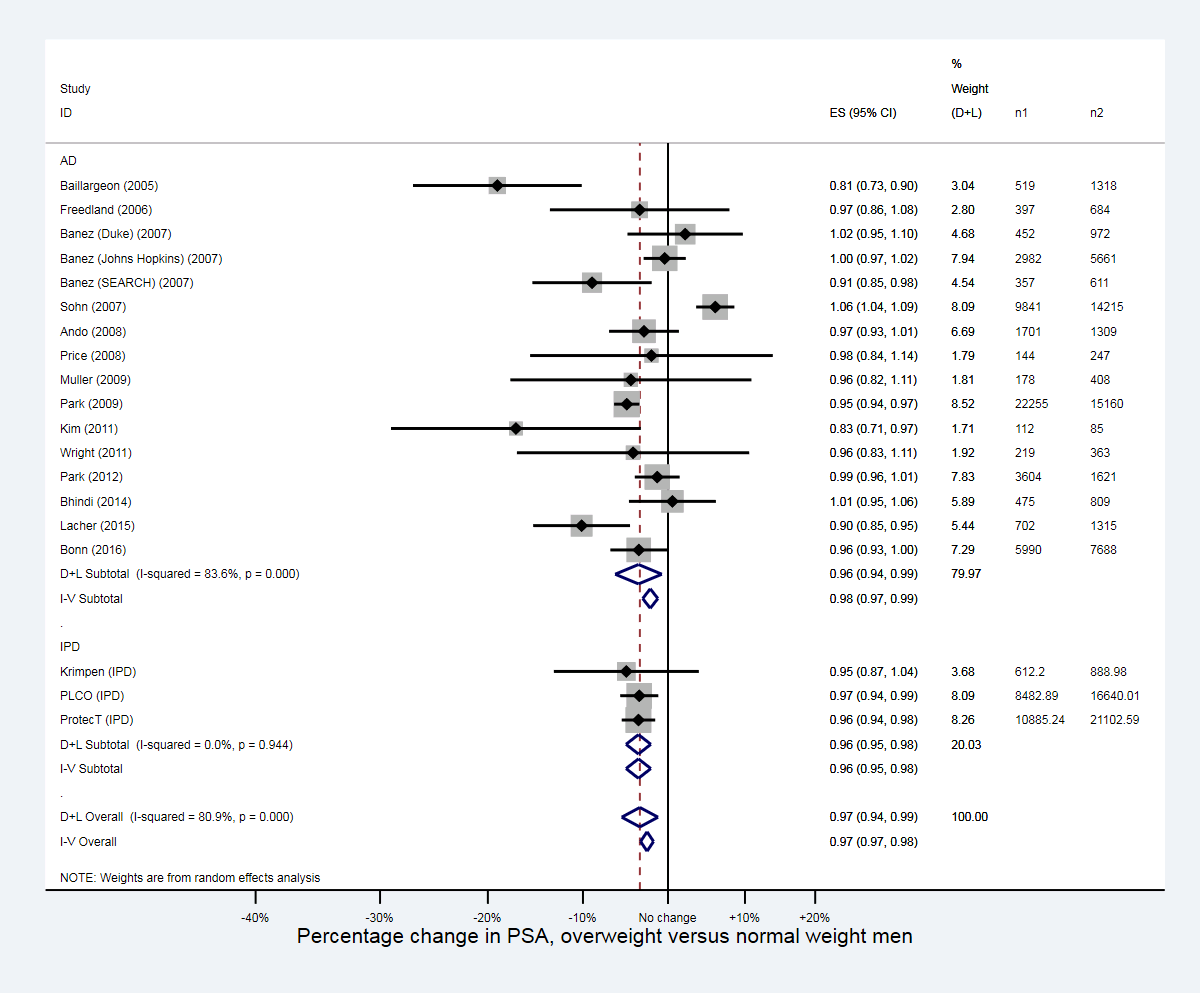


Figure S13 Forest plot of the percentage change in PSA between overweight and normal BMI categories, n1 = number of normal weight participants, n2 = number of overweight participants, AD = aggregate data, IPD = individual participant data


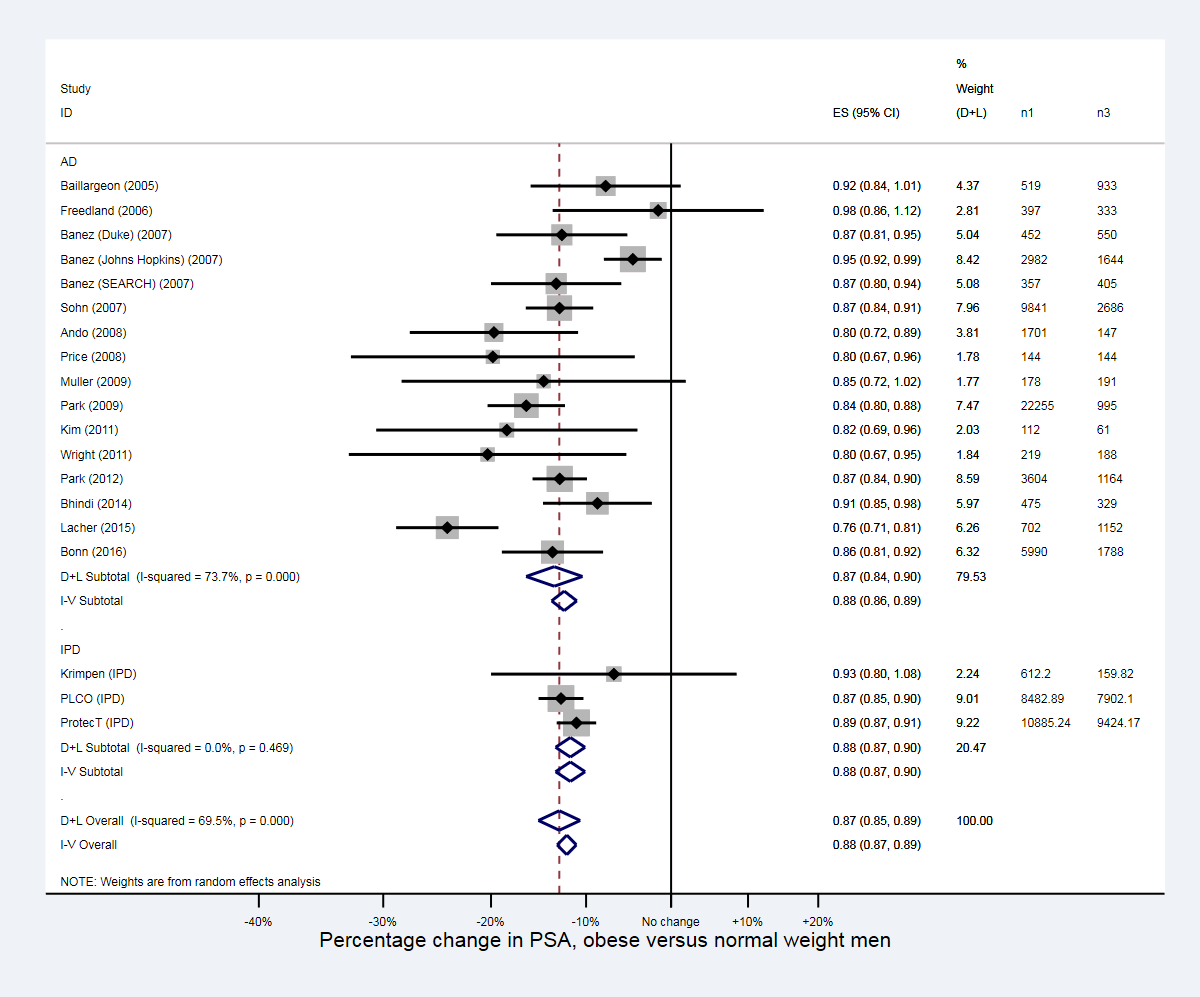


Figure S14 Forest plot of the percentage change in PSA between obese and normal BMI categories, n1 = number of normal weight participants, n3 = number of obese participants, AD = aggregate data, IPD = individual participant data
