# Supplementary material for: Systematic review and meta-analysis of the associations between body mass index, prostate cancer, advanced prostate cancer, and prostate-specific antigen
Source: Cancer Causes Control. 2020 Mar 11;31(5):431–49. doi: 10.1007/s10552-020-01291-3 (PMC7105428; doi:10.1007/s10552-020-01291-3)
Supplement: Supplementary file 3 — Supplementary file3 (DOCX 490 kb) [file 10552_2020_1291_MOESM3_ESM.docx]

Table S1 Data extracted from studies examining the association between BMI and prostate cancer

| Author | Year* | Study Name | Study Location | Ethnicity* | Mid-year* | Variables Adjusted^+^ | Effect estimate* | N total  (cases) | P value* | Effect type* | Years f/u* |
| --- | --- | --- | --- | --- | --- | --- | --- | --- | --- | --- | --- |
| Meta-analysis: HR | | | | | | | | | | | |
| Severson (1) | 1988 |  | Hawaii | Japanese (100%) | 1966 | 1 | 1.31 (0.92 to 1.87) | 7994 (174) | 0.13 | Categorical | 10 |
| Mills (2) | 1989 | 7th Day Adventists | USA | White (100%) | 1974 | 1 | 1.13 (0.84 to 1.52) | 35161 (161) | 0.63 | Categorical | 4 |
| Andersson (3) | 1997 | CIO | Sweden |  | 1972 | 1 | 1.08 (0.99 to 1.17) | 137179 (2364) | 0.10 | Categorical | 15 |
| Cerhan (4) | 1997 | Iowa65+ | USA |  | 1981 | 1 | 1.32 (0.90 to 1.92) | 1049 (70) | 0.10 | Categorical | 6 |
| Giovannucci (5) | 1997 | HPFS | USA |  | 1986 | 1,14,15 | 0.93 (0.83 to 1.05) | 47750 (1338) | 0.46 | Categorical | 6 |
| Nilsen (6) | 1999 | NHSSN | Norway |  | 1983 | 1 | 1.02 (0.90 to 1.16) | 22248 (642) | 0.77 | Categorical | 6 |
| Schuurman (7) | 2000 | NLCS | Netherlands |  | 1986 | 1,4,6 | 1.00 (0.83 to 1.21) | 2240 (675) | 1 | Continuous | 4 |
| Lee (8) | 2001 | HAHS | USA |  | 1988 | 1,4,9,10 | 1.05 (0.82 to 1.34) | 8930 (439) | 0.71 | Categorical | 3 |
| Engeland (9) | 2003 |  | Norway |  | 1966 | 1,16 | 1.07 (1.05 to 1.09) | 951459 (33314) | 0.001 | Categorical | 15 |
| Jonssoni (10) | 2003 | Swedish Twin Study | Sweden |  | 1961 | 1,2,4,17 | 0.98 (0.66 to 1.46) | 662 (331) | 0.94 | Categorical | 20 |
| Kuriyama (11) | 2005 |  | Japan |  | 1984 | 1 | 1.12 (0.65 to 1.94) | 12263 (45) | 0.99 | Categorical | 5 |
| Oh (12) | 2005 | KNHIC | Korea |  | 1992 | 1,4,5,9,10,11 | 1.41 (1.15 to 1.74) | 781283 (387) | 0.001 | Categorical | 5 |
| Haheim (13) | 2006 | OS | Norway |  | 1972 | 1 | 1.10 (0.96 to 1.27) | 15933 (507) | 0.18 | Continuous | 20 |
| Kurahashi (14) | 2006 | JPHC | Japan |  | 1990 | 1,4,5,9,19 | 1.20 (0.95 to 1.50) | 49850 (311) | 0.13 | Categorical | 10 |
| Lukanova (15) | 2006 | NSHDC | Sweden |  | 1985 | 1,9,20 | 0.92 (0.78 to 1.09) | 33424 (461) | 0.31 | Categorical | 5 |
| Lundqvist (16) | 2007 | Finnish/Swedish Twin Cohorts | Scandanavia |  | 1975 | 1,2,4,7,8,9,11 | 1.00 (0.72 to 1.39) | 874 (437) | 0.91 | Continuous | 15 |
|  |  |  |  |  |  |  | 1.16 (0.71 to 1.89) | 430 (215) | 0.58 |  | 20 |
| Rodriguez (17) | 2007 | CPS-II | USA | White (98%) | 1992 | 1,2,4,7,8,9,11,12,13 | 0.98 (0.94 to 1.01) | 69991 (5252) | 0.14 | Categorical | 8 |
| Wright (18) | 2007 | NIH-AARP | USA |  | 1995 | 1,2,4,7,8,9 | 0.97 (0.95 to 0.99) | 287760 (9986) | 0.0008 | Categorical | 3 |
| Hernandez (19) | 2009 | MEC | USA | Multiethnic | 1994 | 1,2,4,8,9,19,24 | 1.00 (0.95 to 1.05) | 83879 (5554) | 0.62 | Categorical | 10 |
| Wallström (20) | 2009 | MDCS | Sweden |  | 1994 | 1,6,7,9,10,11,12,15,25,26 | 0.96 (0.85 to 1.08) | 10548 (817) | 0.58 | Categorical | 10 |
| Burton (21) | 2010 | GAC | Scotland | Caucasian | 1950 | 6,9,15 | 1.00 (0.72 to 1.39) | 9549 (211) | 0.89 | Continuous | 47 |
| Stocks (22) | 2010 | SCWC | Sweden |  | 1981 | 1,9,27 | 1.04 (1.00 to 1.08) | 336159 (10002) | 0.40 | Categorical | 30 |
| Bassett (23) | 2012 | MCCS | Australia |  | 1992 | 2,8 | 1.06 (0.97 to 1.16) | 16514 (1374) | 0.22 | Continuous | 15 |
| Häggström (24) | 2012 | Me-Can | Norway, Sweden, Austria |  |  | 1,9 | 0.99 (0.95 to 1.04) | 296539 (6673) | 0.43 | Categorical | 10 |
| Shafique (25) | 2012 | Midspan Study | Scotland | Caucasian | 1973 | 1,6,9,28 | 1.02 (0.88 to 1.18) | 12924 (650) | 0.79 | Categorical | 24 |
| Rao (26) | 2013 |  | USA | Caucasian (82%) | 2003 | 1,2,7,9,29 | 0.95 (0.93 to 0.98) | 544197 (34275) | 0.0001 | Continuous | 5 |
| Bhaskaran (27) | 2014 | CPRD | UK |  | 1986 | 1,6,7,9,10,20 | 0.98 (0.96 to 1.00) | 5264901 (24901) | 0.004 | Continuous | 5 |
| Møller (28) | 2014 | DCH | Denmark |  | 1995 | 1 | 0.94 (0.88 to 1.02) | 28690 (1813) | 0.14 | Categorical | 10 |
| Choi (29) | 2016 |  | South Korea |  | 2002 | 1,9,10,11 | 1.13 (1.08 to 1.19) | 139519 (7620) | 0.0001 | Categorical | 5 |
| Perez-Cornago (30) | 2017 | EPIC | Europe |  | 1995 | 1,7,8,9,11,19 | 0.94 (0.90 to 0.98) | 141896 (6991) | 0.004 | Continuous | 13 |
| Meta-analysis: OR – BMI measured at least two years before diagnosis (before) | | | | | | | | | | |  |
| Heikkila (31) | 1999 |  | Finland |  | 1969 | 1,5 | 0.84 (0.60 to 1.16) | 466 (166) | 0.45 | MD | 15 |
| Giles (32) | 2003 |  | Australia |  | 1995 | 1 | 1.04 (0.94 to 1.14) | 2885 (1409) | 0.47 | MD | 40 |
| Jian (33) | 2005 |  | China |  | 2001 | 1 | 1.73 (1.11 to 2.71) | 404 (130) | 0.02 | MD | 5 |
| Liu (34) | 2005 |  | USA | White (91%) | 2000 | 1,2,8,12 | 0.96 (0.76 to 1.23) | 902 (434) | 0.73 | Categorical | 39 |
| Porter (35) | 2005 | SEER | USA | Caucasian (95%) | 1994 | 1,2,4,8,9,12,13 | 0.84 (0.70 to 1.00) | 1456 (753) | 0.04 | Categorical | 2 |
| Baillargeon (36) | 2006 | SABOR | USA | Caucasian (86%) | 2001 | 1,2,18 | 0.78 (0.56 to 1.09) | 229 (104) | 0.22 | Categorical | 2 |
| Machova (37) | 2007 |  | Czechia |  | 1995 | 1,9,15,21 | 0.96 (0.77 to 1.21) | 17335 (338) | 0.76 | Categorical | 15 |
| Albanes (38) | 2009 | ATBC | USA |  | 1986 |  | 0.97 (0.74 to 1.27) | 500 (100) | 0.82 | MD | 9 |
| Farhat (39) | 2009 | MrOS | USA |  | 2001 |  | 0.90 (0.76 to 1.06) | 4597 (255) | 0.21 | MD | 3 |
| Stark (40) | 2009 | PHS | USA | Caucasian (100%) | 1982 | 1,2 | 1.12 (0.98 to 1.29) | 2124 (982) | 0.10 | MD | 10 |
| Mori (41) | 2011 |  | Japan |  | 2007 | 1,5 | 0.58 (0.33 to 1.03) | 394 (117) | 0.05 | Categorical | 50 |
| Bhavsar (42) | 2014 | Clue II | USA | White (98%) | 1989 | 1,2 | 0.88 (0.68 to 1.13) | 536 (268) | 0.25 | MD | 6 |
| Stefani (43) | 2016 |  | Uruguay |  | 2002 | 1 | 1.92 (1.54 to 2.39) | 936 (464) | <0.0001 | MD | 5 |
| Kunutsor (44) | 2017 | KIHD | Finland |  | 1986 |  | 0.92 (0.76 to 1.12) | 2390 (230) | 0.40 | MD | 20 |
| Krimpen (45) |  | Krimpen | Netherlands | White |  | 1,2,4 | 1.01 (0.70 to 1.44) | 1661 (165) | 0.97 | IPD | 5 |
| Meta-analysis: OR - BMI measured less than two years before diagnosis of prostate cancer (same time) | | | | | | | | | | | |
| Whittemore (46) | 1995 |  | Hawaii, LA, SF, Vancouver | Blacks | 1990 | 1,2 | 0.91 (0.80 to 1.04) | 1071 (531) | 0.16 | MD | - |
|  |  |  |  | Chinese-American |  |  | 1.14 (0.89 to 1.47) | 555 (283) | 0.29 |  |  |
|  |  |  |  | Japanese-American |  |  | 1.12 (0.91 to 1.39) | 655 (326) | 0.29 |  |  |
|  |  |  |  | Whites |  |  | 1.02 (0.89 to 1.17) | 1019 (515) | 0.72 |  |  |
| Andersson (47) | 1996 | Amoris | Sweden |  | 1990 | 1 | 1.00 (0.76 to 1.32) | 477 (249) | 0.83 | Continuous | - |
| Lagiou (48) | 1998 |  | Greece |  | 1993 | 1 | 0.75 (0.41 to 1.36) | 91 (43) | 0.34 | MD | - |
| Hsieh (49) | 1999 |  | Athens, Greece |  | 1995 | 1 | 0.97 (0.76 to 1.25) | 572 (320) | 0.84 | MD | - |
| Villeneuve (50) | 1999 | NECSS | Canada |  | 1995 | 1,2,4,5,6,9,10,12 | 1.05 (1.02 to 1.09) | 2810 (1413) | 0.37 | Categorical | - |
| Hsing (51) | 2001 |  | China |  | 1994 | 1 | 0.90 (0.65 to 1.25) | 434 (128) | 0.55 | MD | - |
| Sharpe (52) | 2001 |  | Canada |  | 1982 | 1,2,4,10 | 1.22 (0.95 to 1.59) | 869 (396) | 0.12 | Categorical | - |
| Cui (53) | 2004 | NHANES III | USA | White (38%); Black (28%); Mexican-American (30%) | 1997 | 1,2,8,9,10,12 | 0.73 (0.56 to 0.96) | 8815 (95) | 0.02 | Continuous | - |
| Dal Maso (54) | 2004 |  | Italy |  | 1996 | 1,4,5,8,11 | 1.10 (0.95 to 1.26) | 2737 (1290) | 0.23 | Categorical | - |
| Friedenreich (55) | 2004 |  | Canada |  | 1999 | 1,3,4,5,8,10,11,13 | 1.04 (0.91 to 1.19) | 2049 (987) | 0.57 | Categorical | - |
| Bradbury (56) | 2005 |  | UK |  | 1996 | 1,5,7,9 | 0.96 (0.84 to 1.11) | 3200 (730) | 0.04 | Categorical | - |
| Gallus (57) | 2007 |  | Italy |  | 1996 | 1,4,8,5,11 | 1.12 (0.84 to 1.51) | 648 (219) | 0.39 | Categorical | - |
| Chamie (58) | 2008 | Agent Orange | USA |  | 2002 | 1,2,9,18,22,23 | 1.10 (0.95 to 1.28) | 13144 (363) | 0.21 | Continuous | - |
| Chia (59) | 2012 |  | Singapore | Asian |  | 1,2 | 0.60 (0.47 to 0.78) | 396 (212) | 0.0001 | MD | - |
| Fowke (60) | 2012 | NMHS | USA | White (89%) | 2010 | 1,2,4,7,18,32,33,34,35 | 1.00 (1.00 to 1.00) | 1866 (809) | 0.93 | MD | - |
| Nemesure (61) | 2012 | PCBP | Barbados | Black (100%) | 2007 | 1 | 1.00 (0.90 to 1.11) | 1904 (963) | 1 | Continuous | - |
| Yaturu (62) | 2012 |  | USA |  | 2007 | 1 | 0.97 (0.86 to 1.09) | 952 (479) | 0.66 | MD | - |
| Möller (63) | 2013 | CAPS | Sweden | Caucasian | 2001 | 1,5,7,8,11 | 0.93 (0.79 to 1.09) | 1601 (935) | 0.38 | Continuous | - |
| Salem (64) | 2013 |  | Iran |  | 2010 | 1,30,31 | 1.22 (.02 to 94.37) | 511 (194) | 0.35 | Continuous | - |
| Boehm (65) | 2015 | PROtEuS | Canada | European (86%) | 2008 | 1,4 | 0.84 (0.76 to 0.92) | 3903 (1921) | 0.01 | Categorical | - |
| PLCO (66) |  | PLCO | USA | White |  | 1,2,4 | 1.00 (0.95 to 1.04) | 33025 (13461) | 0.83 | IPD | - |
| ProtecT (67) |  | ProtecT | UK | White |  | 1,2,4 | 0.99 (0.95 to 1.04) | 41412 (9841) | 0.82 | IPD | - |
| Albatross only | | | | | | | | | | |  |
| Le Marchand (68) | 1994 |  | Hawaii |  | 1977 | 1,2,6 | Negative | 8881 (198) | 0.20 | RR Trend | 8 |
| Veierød (69) | 1997 |  | Norway |  | 1980 | 1 | Positive | 25778 (70) | 0.02 | IRR Trend | 9 |
| Habel (70) | 2000 | KMCP | California, USA | White (79%), black (12%) | 1968 | 1,2 | Negative | 29117 (28285) | 0.90 | RR BMI>27.9 vs BMI<22.7 | 10 |
| Hsing (71) | 2000 |  | China |  | 1994 | 1,8,12,19 | Positive | 709 (238) | 1 | OR trend | - |
| Putnam (72) | 2000 |  | Iowa, USA | USA | 1987 | 1,4,12 | Positive | 1555 (81) | 0.08 | RR trend | 4 |
| Pan (73) | 2004 | NECSS | Canada |  | 1995 | 1,5,8,9,11,12 | Positive | 4348 (1801) | 0.03 | RR trend |  |
| Cox (74) | 2006 |  | New Zealand | European descent | 1997 | 1 | Negative | 495 (199) | 0.08 | RR BMI q5 vs BMI q1 | 4 |
| Littman (75) | 2007 | VITAL | USA | White (93%) | 2001 | 1,2,4,13 | Negative | 34868 (808) | 0.13 | HR trend | 2 |
| Attner (76) | 2012 | Skaine Study | Sweden |  | 2006 | 1 | Positive | 30199 (3545) | 0.08 | RR obesity | 2 |
| Harding (77) | 2015 | ANZDCC | Australia/NZ |  | 1993 | 1 | Negative | 40734 (2866) | 0.63 | HR trend | 8 |
| Heir (78) | 2016 | Oslo Ischaemia Study | Norway |  | 1973 | 1,9,11,27 | Negative | 1997 (213) | 0.01 | RR BMI>25 vs BMI<25 | 20 |
| **Year = publication year, Ethnicity = Ethnicity (%) if specified, Mid-year = the mid-year of recruitment for each study (publication data minus 1 year if not reported) Effect estimate = HR or OR for prostate cancer for a 5 kg/m^2^ increase in BMI (meta-analysis) or effect direction (albatross), P value = P value for effect estimate (taken from P for trend for categorical effects, if available), Effect type = original presentation of data (meta-analysis) or to which effect estimate the P value refers (albatross), Years f/u = average years between BMI measurement or estimate and prostate cancer diagnosis (studies with 2 or more years between estimates only), estimated if not stated in paper* | | | | | | | | | | | |
| *^+^Variables adjusted for: 1 = Age, 2 = Ethnicity, 3 = DRE, 4 = Family History, 5 = Area, 6 = Income, 7 = Diabetes, 8 = Education, 9 = Smoking, 10 = Alcohol, 11 = Physical Activity, 12 = Diet, 13 = PSA test, 14 = BMI at younger age, 15 = Height, 16 = Cohort, 17 = Twins, 18 = PSA, 19 = Marital Status, 20 = Year, 21 = Hypertension, 22 = Finasteride, 23 = Agent Orange Exposure, 24 = Birthplace, 25 = Co-habitation Status, 26 = Birth Country, 27 = Blood Pressure, 28 = Cholesterol, 29 = Angiotensin receptor blocker, 30 = Calcium, 31 = Sex hormones, 32 = BPH, 33 = prostate volume, 34 = Cardiovascular disease, 35 = Hyperlipidaemia treatments* | | | | | | | | | | | |
| *Study Acronyms: AMORIS = The Swedish Apolipoprotein MOrtality RISk study, ANZDCC = Australia and New Zealand Diabetes and Cancer Collaboration, ATBC = Alpha-Tocopherol, Beta-Carotene cancer prevention study, CAPS = Cancer of the Prostate in Sweden, CIO = The Construction Industry’s Organization for working environment, safety and health, CIOWE = The Construction Industry’s Organization for Working Environment (Safety and Health), CLUE II = Campaign Against Cancer and Heart Disease, CPRD = Clinical Practice Research Datalink, CPS-II = Cancer Prevention Study II, DCH = Danish Diet, Cancer and Health, EPIC = European Prospective Investigation into Cancer and Nutrition, GAC = Glasgow Alumni Cohort, HAHS = Harvard Alumni Health Study, HPFS = Health Professionals Follow-Up Study, Iowa 65+ = Iowa 65+ rural health study, JPHC = Japan Public Health Center-based Prospective Study, KIHD = Kuopio Ischemic Heart Disease risk factor study, KMCP = Kaiser Permanente Medical Care Program, KNHIC = Korea National Health Insurance Company, KPMCP = Kaiser Permanente medical care program, MCCS = Melbourne Collaborative Cohort Study, MDCS = Malmo Diet and Cancer, Me-Can = Metabolic syndrome and Cancer, MEC = MultiEthnic Cohort, MrOS = Osteopathic Fracture in Men study, NECSS = Canadian National Enhanced Cancer Surveillance System, NHANES III = Third National Health and Nutrition Examination Survey, NHSSN = National Health Screening Service in Norway, NIH-AARP = National Institute of Health American Association of Retired Persons diet and health study, NLCS = The Netherlands cohort study, NMHS = Nashville Men's Health Study, NSHDC = North Sweden Health and Disease Cohort, OS = Oslo Study, PCBP = Prostate Cancer in a Black Population, PHS = Physicians’ Health Study, PLCO = Prostate, Lung, Colorectal, and Ovarian Cancer Screening Trial, ProtecT = Prostate Testing for Cancer and Treatment, PRTOtEuS = Prospective, randomized, multicentre, open label, phase II / III study to assess efficacy and safety of ranibizumab 0.5 mg intravitreal injections plus panretinal photocoagulation (PRP) versus PRP in monotherapy in the treatment of subjects with high risk proliferative diabetic retinopathy, SABOR = San Antonio Center for Biomarkers of Risk of prostate carcinoma, SCWC = Swedish Construction Workers Cohort, SEER = Surveillance, Epidemiology and End Results Program, VITAL = Vitamins and lifestyle cohort* | | | | | | | | | | | |

Table S2 Risk of bias of studies examining the association between BMI and prostate cancer

| Author | Overall RoB | Confounding | Participants | Missing Data | Measurement of Outcome | Measurement of Exposure | Selective Reporting |
| --- | --- | --- | --- | --- | --- | --- | --- |
| Meta-analysis: HR | | | | | | | |
| Severson (1) | High | Medium | Low | Medium | Medium | Medium | Low |
| Mills (2) | High | Medium | Medium | Low | Medium | Medium | Low |
| Andersson (3) | Medium | Medium | Low | Medium | Medium | Low | Low |
| Cerhan (4) | High | Medium | Low | Medium | Medium | Medium | Low |
| Giovannucci (5) | High | Medium | Low | Medium | Medium | Medium | Low |
| Nilsen (6) | Medium | Medium | Low | Low | Medium | Low | Low |
| Schuurman (7) | High | Medium | Low | Medium | Medium | Medium | Low |
| Lee (8) | High | Medium | Low | Medium | Medium | Medium | Low |
| Engeland (9) | Medium | Medium | Low | Medium | Medium | Low | Low |
| Jonssoni (10) | Medium | Medium | Low | Low | Medium | Medium | Low |
| Kuriyama (11) | Medium | Medium | Low | Medium | Medium | Medium | Low |
| Oh (12) | Medium | Medium | Low | Medium | Low | Medium | Low |
| Haheim (13) | Medium | Medium | Low | Low | Low | Low | Low |
| Kurahashi (14) | Medium | Medium | Low | Medium | Medium | Medium | Low |
| Lukanova (15) | High | High | Low | Medium | Medium | Medium | Low |
| Lundqvist (16) | Medium | Low | Low | Medium | Low | Medium | Low |
| Rodriguez (17) | High | Medium | Low | Medium | Medium | Medium | Low |
| Wright (18) | Medium | Medium | Low | Medium | Medium | Medium | Low |
| Hernandez (19) | Medium | Medium | Low | Medium | Medium | Medium | Low |
| Wallström (20) | High | High | Low | Medium | Medium | Low | Low |
| Burton (21) | High | High | Low | Low | Low | Low | Low |
| Stocks (22) | Medium | Medium | Low | Low | Low | Low | Low |
| Bassett (23) | High | High | Low | Medium | Medium | Low | Low |
| Häggström (24) | Medium | Medium | Low | Medium | Medium | Medium | Low |
| Shafique (25) | Medium | Medium | Low | Low | Low | Low | Low |
| Rao (26) | High | Medium | High | Low | Unclear | Unclear | Low |
| Bhaskaran (27) | Medium | Medium | Low | Medium | Medium | Medium | Low |
| Møller (28) | Medium | Medium | Low | Low | Low | Low | Low |
| Choi (29) | Medium | Medium | Low | Low | Low | Low | Low |
| Perez-Cornago (30) | Medium | Medium | Low | Low | Low | Low | Low |
| Meta-analysis: OR – BMI measured at least two years before diagnosis (before) | | | | | | | |
| Heikkila (31) | Medium | Medium | Low | Low | Medium | Low | Low |
| Giles (32) | Medium | Medium | Low | Low | Medium | Medium | Low |
| Jian (33) | High | High | Medium | Low | Low | High | Low |
| Liu (34) | Medium | Medium | Medium | Low | Medium | Medium | Low |
| Porter (35) | Medium | Medium | Medium | Medium | Medium | Medium | Low |
| Baillargeon (36) | Medium | Medium | Low | Low | Medium | Low | Low |
| Machova (37) | Medium | Medium | Low | Low | Medium | Low | Low |
| Albanes (38) | High | High | Medium | Low | Low | Low | Low |
| Farhat (39) | High | High | Low | Medium | Medium | Medium | Low |
| Stark (40) | High | Medium | Medium | Medium | Medium | Medium | Low |
| Mori (41) | High | Medium | Medium | Low | Medium | Medium | Low |
| Bhavsar (42) | Medium | Medium | Low | Low | Medium | Low | Low |
| Boehm (65) | Medium | Medium | Low | Low | Low | Low | Low |
| Stefani (43) | High | High | Medium | Low | Low | Low | Low |
| Kunutsor (44) | High | High | Low | Low | Low | Low | Low |
| Krimpen (45) | Medium | Medium | Low | Low | Low | Low | Low |
| Meta-analysis: OR - BMI measured less than two years before diagnosis of prostate cancer (same time) | | | | | | | |
| Whittemore (46) | High | High | Medium | Medium | Medium | Medium | Low |
| Andersson (47) | Medium | Medium | Medium | Medium | Medium | Low | Low |
| Lagiou (48) | High | High | Low | Low | Medium | Low | Low |
| Hsieh (49) | High | High | Medium | Medium | Medium | Medium | Low |
| Villeneuve (50) | Medium | Medium | Low | Medium | Medium | Medium | Low |
| Hsing (51) | High | High | High | High | Medium | Low | Low |
| Sharpe (52) | Medium | Medium | Low | Medium | Medium | Medium | Low |
| Cui (53) | Medium | Medium | Low | Low | Medium | Medium | Low |
| Dal Maso (54) | High | Medium | Medium | Low | Medium | Medium | Low |
| Friedenreich (55) | Medium | Medium | Low | Low | Medium | Low | Low |
| Bradbury (56) | Medium | Medium | Low | Low | Medium | Medium | Low |
| Gallus (57) | High | Medium | Medium | Low | Medium | Medium | Low |
| Chamie (58) | High | Medium | Medium | Medium | Medium | Medium | Low |
| Chia (59) | High | High | Medium | Medium | Low | Medium | Low |
| Fowke (60) | High | Medium | Unclear | Low | Low | Low | Low |
| Nemesure (61) | High | High | Medium | Low | Low | Low | Low |
| Yaturu (62) | High | High | High | Low | Medium | Low | Low |
| Möller (63) | High | Medium | Low | Medium | Medium | Medium | Low |
| Salem (64) | High | High | High | Low | Low | Unclear | Low |
| ProtecT (67) | Medium | Medium | Low | Low | Low | Low | Low |
| PLCO (66) | Medium | Medium | Low | Low | Low | Low | Low |
| Albatross only |  |  |  |  |  |  |  |
| Le Marchand (68) | High | Medium | Low | Medium | Medium | Medium | Low |
| Veierød (69) | High | Medium | Low | Medium | Medium | Medium | Low |
| Habel (70) | Medium | Medium | Low | Medium | Medium | Low | Low |
| Hsing (71) | High | Medium | Low | Medium | Medium | Medium | Low |
| Putnam (72) | High | Medium | Low | Medium | Medium | Medium | Low |
| Pan (73) | Medium | Medium | Low | Medium | Medium | Medium | Low |
| Cox (74) | High | Medium | Medium | Medium | Medium | Medium | Low |
| Littman (75) | Medium | Medium | Low | Low | Medium | Medium | Low |
| Attner (76) | Medium | Medium | Low | Medium | Medium | Medium | Low |
| Harding (77) | Medium | Medium | Low | Medium | Medium | Low | Low |
| Heir (78) | Medium | Medium | Low | Low | Low | Low | Low |

Table S3 Meta-regression results for the linear association between BMI and prostate cancer

| Variable | HR | | | OR | | |
| --- | --- | --- | --- | --- | --- | --- |
|  | **Ratio of HRs** | **95% CI** | **P value** | **Ratio of ORs** | **95% CI** | **P value** |
| Ethnicity (white versus non-white) | 1.04 | 0.87 – 1.24 | 0.57 | 0.84 | 0.66 – 1.06 | 0.14 |
| Risk of bias (high versus medium risk) | 1.01 | 0.88 – 1.16 | 0.81 | 1.13 | 0.97 – 1.33 | 0.12 |
| Mid-year of study recruitment | 1.00 | 0.99 – 1.00 | 0.11 | 1.00 | 0.99 – 1.01 | 0.57 |
| Mean BMI in study | 0.99 | 0.88 – 1.10 | 0.76 | 0.96 | 0.91 – 1.01 | 0.10 |
| Years between BMI measurement and diagnosis | 1.00 | 1.00 – 1.01 | 0.87 | 1.00 | 0.99 – 1.00 | 0.38 |
| Age at diagnosis | 1.00 | 0.98 – 1.01 | 0.71 | 1.00 | 0.98 – 1.02 | 0.83 |

Table S4 Summary BMI and number of men with and without prostate cancer for normal, overweight and obese BMI categories.

| Author/Study | Normal weight (<25 kg/m^2^) | | | Overweight  (25-29.9 kg/m^2^) | | | Obese (≥30 kg/m^2^) | | |
| --- | --- | --- | --- | --- | --- | --- | --- | --- | --- |
|  | BMI  (kg/m^2^) | Cases | Controls | BMI  (kg/m^2^) | Cases | Controls | BMI  (kg/m^2^) | Cases | Controls |
| Meta-analysis: HR | | | | | | | | | |
| Jonssoni (10) | 23.1 | 190 | 185 | 27.1 | 125 | 136 | 30.8 | 13 | 8 |
| Rodriguez (17) | 21.6 | 1935 | 23167 | No data | No data | No data | 32.1 | 556 | 7809 |
| Wright (18) | 21.2 | 3076 | NK | 27.5 | 5054 | NK | 32.8 | 1532 | NK |
| Shafique (25) | 22.7 | 279 | 5499 | 27.1 | 320 | 5729 | 31.4 | 51 | 1046 |
| Park (79) | NK | NK | NK | NK | NK | NK | NK | NK | NK |
| Meta-analysis: OR – BMI measured at least two years before diagnosis (before) | | | | | | | | | |
| Liu (34) | 22.6 | 106 | 119 | No data | No data | No data | 31.7 | 106 | 123 |
| Porter (35) | 22.2 | 195 | 173 | No data | No data | No data | 31.0 | 178 | 175 |
| Baillargeon (36) | 22.7 | 21 | 22 | 27.6 | 50 | 47 | 33.0 | 33 | 56 |
| Machova (37) | 23.6 | 42 | 2159 | 27.6 | 210 | 10273 | 31.6 | 86 | 4565 |
| Boehm (65) | 22.5 | 648 | 609 | 27.4 | 922 | 940 | 32.1 | 351 | 433 |
| Krimpen (45) | 23.2 | 63 | 549 | 27.0 | 89 | 799 | 31.9 | 17 | 143 |
| Meta-analysis: OR - BMI measured less than two years before diagnosis of prostate cancer (same time) | | | | | | | | | |
| PLCO (66) | 23.1 | 3,439 | 5,044 | 27.2 | 6,860 | 9,779 | 33.3 | 3,158 | 4,745 |
| ProtecT (67) | 23.1 | 2,574 | 8,312 | 27.3 | 5,072 | 16,030 | 33.1 | 2,192 | 7,231 |
| *PLCO = Prostate, Lung, Colorectal and Ovarian Cancer Screening Trial, ProtecT = Prostate Testing for Cancer and Treatment study*  *NK = not known*  *Note: BMI values for the IPD are taken from the Continuous imputation of BMI, whereas the number of cases and controls are taken from the Categorical imputation of BMI* | | | | | | | | | |

Table S5 Results for the Categorical analysis of the association between BMI and prostate cancer

| Author/Study | OR for prostate cancer | |
| --- | --- | --- |
|  | Overweight vs normal (95% CI) | Obese vs normal (95% CI) |
| Meta-analysis: HR | | |
| Jonssoni (10) | 0.90 (0.61 to 1.32) | 1.60 (0.61 to 4.18) |
| Rodriguez (17) | No data | 0.94 (0.85 to 1.04) |
| Wright (18) | 1.00 (0.96 to 1.05) | 0.97 (0.91 to 1.03) |
| Shafique (25) | 1.02 (0.86 to 1.20) | 1.03 (0.76 to 1.39) |
| Park (79) | 1.04 (0.99 to 1.10) | 0.98 (0.91 to 1.06) |
| Meta-analysis: OR – BMI measured at least two years before diagnosis (before) | | |
| Liu (34) | No data | 0.96 (0.61 to 1.51) |
| Porter (35) | No data | 0.77 (0.56 to 1.06) |
| Baillargeon (36) | 1.45 (0.73 to 2.89) | 0.72 (0.36 to 1.45) |
| Machova (37) | 1.05 (0.76 to 1.46) | 0.97 (0.66 to 1.42) |
| Boehm (65) | 0.83 (0.71 to 0.97) | 0.71 (0.59 to 0.85) |
| Krimpen (45) | 0.98 (0.63 to 1.52) | 1.08 (0.52 to 2.25) |
| Meta-analysis: OR - BMI measured less than two years before diagnosis of prostate cancer (same time) | | |
| PLCO (66) | 1.03 (0.95 to 1.12) | 0.98 (0.88 to 1.08) |
| ProtecT (67) | 1.02 (0.93 to 1.13) | 0.98 (0.88 to 1.09) |
| *PLCO = Prostate, Lung, Colorectal and Ovarian Cancer Screening Trial, ProtecT = Prostate Testing for Cancer and Treatment study* | | |

Table S6 Data extracted from studies examining the association between BMI and advanced prostate cancer

| Author | Year* | Study Name | Study Location | Ethnicity* | Mid-year* | Variables Adjusted^+^ | Effect estimate* | N total  (cases) | P value* | Effect type* | Years f/u* |
| --- | --- | --- | --- | --- | --- | --- | --- | --- | --- | --- | --- |
| Meta-analysis: HR | | | | | | | | | | | |
| Giovannucci (5) | 1997 | HPFS | USA |  | 1986 | 1,14,15 | 1.05 (0.86 to 1.29) | 46819 (407) | 0.16 | Categorical | 6 |
| Schuurman (7) | 2000 | NLCS | Netherlands |  | 1986 | 1,4,6 | 1.03 (0.77 to 1.36) | 2246 (228) | 0.87 | Continuous | 4 |
| Kurahashi (14) | 2006 | JPHC | Japan |  | 1990 | 1,4,5,9,19 | 1.32 (0.88 to 1.98) | 49630 (91) | 0.16 | Categorical | 10 |
| Rodriguez (17) | 2007 | CPS-II | USA | White (98%) | 1992 | 1,2,4,7,8,9,11,12,13 | 1.24 (1.01 to 1.51) | 63472 (288) | 0.04 | Categorical | 8 |
| Wright (18) | 2007 | NIH-AARP | USA |  | 1995 | 1,2,4,7,8,9 | 1.01 (0.94 to 1.08) | 279220 (1446) | 0.64 | Categorical | 3 |
| Hernandez (19) | 2009 | MEC | USA | Multiethnic | 1994 | 1,2,4,8,9,19,24 | 1.00 (0.86 to 1.17) | 78957 (632) | 0.83 | Categorical | 10 |
| Wallström (20) | 2009 | MDCS | Sweden |  | 1994 | 1,6,7,9,10,11,12,15,25,26 | 0.92 (0.75 to 1.13) | 10012 (281) | 0.49 | Categorical | 10 |
| Stocks (22) | 2010 | SCWC | Sweden |  | 1981 | 1,9,27 | 1.09 (1.00 to 1.19) | 328559 (2402) | 0.1 | Categorical | 30 |
| Discacciati (80) | 2011 |  | Sweden |  | 1997 | 1,4,7,8,9,11,12,14 | 1.05 (0.89 to 1.23) | 35983 (554) | 0.55 | Continuous | 5 |
| Bassett (23) | 2012 | MCCS | Australia |  | 1992 | 2,8 | 1.27 (1.08 to 1.49) | 15550 (410) | 0.004 | Continuous | 15 |
| Perez-Cornago (30) | 2017 | EPIC | Europe |  | 1995 | 1,7,8,9,11,19 | 1.05 (0.96 to 1.15) | 141896 (1384) | 0.29 | Continuous | 13 |
| Meta-analysis: OR - BMI measured at least two years before diagnosis of prostate cancer (before) | | | | | | | | | | | |
| Robinson (81) | 2005 | SEER | USA | White (81%) | 1999 | 1 | 0.88 (0.75 to 1.04) | 1111 (568) | 0.13 | MD | 40 |
| Geybels (82) | 2013 | NLCS | Netherlands |  | 1986 |  | 1.10 (0.94 to 1.29) | 3292 (1164) | 0.22 | MD | 10 |
| Krimpen (45) |  | Krimpen | Netherlands | White |  | 1,2,4 | 0.91 (0.30 to 2.81) | 1661 (15) | 0.88 | IPD | 5 |
| Meta-analysis: OR - BMI measured less than two years before diagnosis of prostate cancer (same time) | | | | | | | | | | | |
| Chamie (58) | 2008 | Agent Orange | USA |  | 2002 | 1,2,9,18,22,23 | 0.86 (0.65 to 1.14) | 12846 (65) | 0.34 | Continuous | - |
| Möller (63) | 2013 | CAPS | Sweden | Caucasian | 2001 | 1,5,7,8,11 | 1.01 (0.84 to 1.22) | 1156 (490) | 0.92 | Continuous | - |
| PLCO (66) |  | PLCO | USA | White |  | 1,2,4 | 1.02 (0.87 to 1.13) | 33025 (1106) | 0.73 | IPD | - |
| ProtecT (67) |  | ProtecT | UK | White |  | 1,2,4 | 1.01 (0.87 to 1.17) | 41412 (494) | 0.87 | IPD | - |
| Albatross only | | | | | | | | | | | |
| Hsing (71) | 2000 |  | China |  | 1994 | 1,8,12,19 | Positive | 621 (150) | 0.73 | OR trend | - |
| Putnam (72) | 2000 |  | Iowa, USA |  | 1987 | 1,4,12 | Positive | 1,492 (18) | 0.02 | RR trend | 4 |
| Littman (75) | 2007 | VITAL | USA | White (93%) | 2001 | 1,2,4,13 | Positive | 33,998 (373) | 0.69 | HR trend | 2 |
| **Year = publication year, Ethnicity = Ethnicity (%) if specified, Mid-year = the mid-year of recruitment for each study (publication data minus 1 year if not reported) Effect estimate = HR or OR for advanced prostate cancer for a 5 kg/m^2^ increase in BMI (meta-analysis) or effect direction (albatross), P value = P value for effect estimate (taken from P for trend for categorical effects, if available), Effect type = original presentation of data (meta-analysis) or to which effect estimate the P value refers (albatross), Years f/u = average years between BMI measurement or estimate and prostate cancer diagnosis (studies with 2 or more years between estimates only), estimated if not stated in paper* | | | | | | | | | | | |
| *^+^Variables adjusted: 1 = Age, 2 = Ethnicity, 3 = DRE, 4 = Family History, 5 = Area, 6 = Income, 7 = Diabetes, 8 = Education, 9 = Smoking, 10 = Alcohol, 11 = Physical Activity, 12 = Diet, 13 = PSA test, 14 = BMI at younger age, 15 = Height, 16 = Cohort, 17 = Twins, 18 = PSA, 19 = Marital Status, 20 = Year, 21 = Hypertension, 22 = Finasteride, 23 = Agent Orange Exposure, 24 = Birthplace, 25 = Co-habitation Status, 26 = Birth Country, 27 = Blood Pressure, 28 = Cholesterol, 29 = Angiotensin receptor blocker, 30 = Calcium, 31 = Sex hormones, 32 = BPH, 33 = prostate volume, 34 = Cardiovascular disease, 35 = Hyperlipidaemia treatments* | | | | | | | | | | | |
| *Study acronyms: CAPS = Cancer of the Prostate in Sweden, CPS-II = Cancer Prevention Study II, EPIC = European Prospective Investigation into Cancer and Nutrition, HPFS = Health Professionals Follow-Up Study, JPHC = Japan Public Health Center-based Prospective Study, MCCS = Melbourne Collaborative Cohort Study, MDCS = Malmo Diet and Cancer Study, MEC = MultiEthnic Cohort, NIH-AARP = National Institute of Health American Association of Retired Persons diet and health study, NLCS = The Netherlands cohort study, PLCO = Prostate, Lung, Colorectal, and Ovarian Cancer Screening Trial, ProtecT = Prostate Testing for Cancer and Treatment, SCWC = Swedish Construction Workers Cohort, SEER = Surveillance, Epidemiology and End Results Program, VITAL = Vitamins and lifestyle cohort* | | | | | | | | | | | |

Table S7 Risk of bias of studies examining the association between BMI and advanced prostate cancer

| Author | Overall RoB | Confounding | Participants | Missing Data | Measurement of Outcome | Measurement of Exposure | Selective Reporting |
| --- | --- | --- | --- | --- | --- | --- | --- |
| Meta-analysis: HR | | | | | | | |
| Giovannucci (5) | High | Medium | Low | Medium | Medium | Medium | Low |
| Schuurman (7) | High | Medium | Low | Medium | Medium | Medium | Low |
| Kurahashi (14) | Medium | Medium | Low | Medium | Medium | Medium | Low |
| Rodriguez (17) | High | Medium | Low | Medium | Medium | Medium | Low |
| Wright (18) | Medium | Medium | Low | Medium | Medium | Medium | Low |
| Hernandez (19) | Medium | Medium | Low | Medium | Medium | Medium | Low |
| Wallström (20) | High | High | Low | Medium | Medium | Low | Low |
| Stocks (22) | Medium | Medium | Low | Low | Low | Low | Low |
| Discacciati (80) | Medium | Medium | Low | Medium | Medium | Medium | Low |
| Bassett (23) | High | High | Low | Medium | Medium | Low | Low |
| Perez-Cornago (30) | Medium | Medium | Low | Low | Low | Low | Low |
| Meta-analysis: OR - BMI measured at least two years before diagnosis of prostate cancer (before) | | | | | | | |
| Robinson (81) | Medium | Medium | Low | Medium | Low | Medium | Low |
| Geybels (82) | High | High | Low | Low | Medium | Low | Low |
| Krimpen (45) | Medium | Medium | Low | Low | Low | Low | Low |
| Meta-analysis: OR - BMI measured less than two years before diagnosis of prostate cancer (same time) | | | | | | | |
| Chamie (58) | High | Medium | Medium | Medium | Medium | Medium | Low |
| Möller (63) | High | Medium | Low | Medium | Medium | Medium | Low |
| PLCO (66) | Medium | Medium | Low | Low | Low | Low | Low |
| ProtecT (67) | Medium | Medium | Low | Low | Low | Low | Low |
| Albatross only | | | | | | | |
| Hsing (71) | High | Medium | Low | Medium | Medium | Medium | Low |
| Putnam (72) | High | Medium | Low | Medium | Medium | Medium | Low |
| Littman (75) | Medium | Medium | Low | Low | Medium | Medium | Low |

Table S8 Meta-regression results for the linear association between BMI and advanced prostate cancer

| Variable | HR | | | OR | | |
| --- | --- | --- | --- | --- | --- | --- |
|  | **Ratio of HRs** | **95% CI** | **P value** | **Ratio of ORs** | **95% CI** | **P value** |
| Ethnicity (white versus non-white) | 0.96 | 0.55 – 1.70 | 0.80 | NA | NA | NA |
| Risk of bias (high versus medium risk) | 1.18 | 0.84 – 1.68 | 0.17 | 0.98 | 0.67 – 1.42 | 0.81 |
| Mid-year of study recruitment | 1.01 | 0.97 – 1.05 | 0.52 | 0.99 | 0.93 – 1.06 | 0.72 |
| Mean BMI in study* | 0.92 | 0.49 – 1.71 | 0.62 | 0.99 | 0.60 – 1.66 | 0.91 |
| Years between BMI measurement and diagnosis | 1.01 | 0.98 – 1.04 | 0.42 | 1.00 | 0.98 – 1.01 | 0.39 |
| Age at diagnosis | 1.00 | 0.95 – 1.05 | 0.99 | 1.00 | 0.92 – 1.10 | 0.85 |
| *Mean BMI in study was missing in several studies, as was age at diagnosis. Due to the low number of studies, two meta-regressions were conducted each for studies presenting HRs and ORs, one leaving out mean BMI for all other ratio of HRs/ORs, and one leaving out either age at diagnosis (HR) or risk of bias (OR) for the ratio of HRs/ORs for mean BMI in study. In no meta-regressions was any P value below 0.15. | | | | | | |

Table S9 Summary BMI and number of men with and without advanced prostate cancer for normal, overweight and obese BMI categories

| Author/Study | Normal weight (<25 kg/m^2^) | | | Overweight (25-29.9 kg/m^2^) | | | Obese (≥30 kg/m^2^) | | |
| --- | --- | --- | --- | --- | --- | --- | --- | --- | --- |
|  | BMI  (kg/m^2^) | Cases | Controls | BMI  (kg/m^2^) | Cases | Controls | BMI  (kg/m^2^) | Cases | Controls |
| Meta-analysis: HR | | | | | | | | | |
| Rodriguez (17) | 22.7 | 92 | 23,167 | No data | No data | No data | 31.5 | 46 | 7,809 |
| Wright (18) | 22.3 | 424 | NK | 27.5 | 726 | NK | 32.0 | 256 | NK |
| Hernandez (19) | 23.0 | 267 | 32,779 | 27.2 | 281 | 33,438 | 31.3 | 77 | 11,245 |
| Meta-analysis: OR | | | | | | | | | |
| Krimpen (45) | 23.2 | 6 | 607 | 27.0 | 7 | 8,81 | 31.9 | 3 | 158 |
| PLCO (66) | 23.1 | 268 | 8,215 | 27.2 | 590 | 16,049 | 33.3 | 254 | 7,649 |
| ProtecT (67) | 23.1 | 131 | 10,876 | 27.3 | 257 | 20,845 | 33.1 | 107 | 9,316 |
| *PLCO = Prostate, Lung, Colorectal and Ovarian Cancer Screening Trial, ProtecT = Prostate Testing for Cancer and Treatment study*  *NK = not known*  *Note: BMI values for the IPD are taken from the Continuous imputation of BMI, whereas the number of cases and controls are taken from the Categorical imputation of BMI* | | | | | | | | | |

Table S10 Results for the Categorical analysis of the association between BMI and advanced prostate cancer

| Author/Study | OR for advanced prostate cancer | |
| --- | --- | --- |
|  | Overweight vs normal (95% CI) | Obese vs normal (95% CI) |
| Meta-analysis: HR | | |
| Rodriguez (17) | No data | 1.54 (1.06 to 2.23) |
| Wright (18) | 1.03 (0.91 to 1.16) | 1.14 (0.97 to 1.33) |
| Hernandez (19) | 1.07 (0.88 to 1.30) | 0.93 (0.69 to 1.25) |
| Meta-analysis: OR | | |
| Krimpen (45) | 0.79 (0.17 to 3.80) | 1.87 (0.33 to 10.52) |
| PLCO (66) | 1.13 (0.91 to 1.40) | 1.02 (0.79 to 1.31) |
| ProtecT (67) | 1.02 (0.76 to 1.36) | 0.95 (0.68 to 1.33) |
| *PLCO = Prostate, Lung, Colorectal and Ovarian Cancer Screening Trial, ProtecT = Prostate Testing for Cancer and Treatment study* | | |

Table S11 Data extracted from studies examining the association between BMI and PSA

| Author | Year | Study Name | Study Location | Ethnicity | Mid-year | Variables adjusted | Effect estimate | Participants | P value | Effect type |
| --- | --- | --- | --- | --- | --- | --- | --- | --- | --- | --- |
| Meta-analysis | | | | | | | | | | |
| Baillargeon (83) | 2005 | SABOR | USA | White (86.6%) | 2002 | 1,2 | -1.51% (-4.52% to 1.60%) | 2770 | 0.34 | Categories |
| Freedland (84) | 2006 |  | USA | White (61%); Black (29%) | 1996 | 1,2,5 | -1.36% (-6.70% to 4.27%) | 1414 | 0.63 | Categories |
| Bañez (85) | 2007 | Duke | USA | White (84%), Black (15%) | 1995 | 1,2,5 | -7.39% (-10.93% to -3.72%) | 1974 | 0.0001 | Categories |
|  |  | Johns Hopkins |  | White (91%), Black (6%) |  |  | -2.35% (-4.16% to -0.51%) | 10287 | 0.01 |  |
|  |  | SEARCH |  | White (52%), Black (41%) |  |  | -6.40% (-9.42% to -3.28%) | 1373 | <0.0001 |  |
| Sohn (86) | 2007 |  | Korea |  | 2001 | 1 | -2.64% (-4.82% to -0.41%) | 26742 | 0.02 | Categories |
| Ando (87) | 2008 |  | Japan | Asian | 2005 | 1 | -4.22% (-6.99% to -1.37%) | 3157 | 0.004 | Categories |
| Price (88) | 2008 |  | USA | White (37%), Black (59%) | 2006 | 1,2,3,4,8,9 | -9.34% (-15.65% to -2.55%) | 535 | 0.008 | Categories |
| Muller (89) | 2009 | ESTHER | Germany |  | 2001 | 1 | -8.37% (-16.94% to 1.09%) | 777 | 0.08 | Categories |
| Park (90) | 2009 |  | Korea |  | 2006 | 1 | -7.17% (-8.59% to -5.72%) | 38410 | <0.0001 | Categories |
| Waters (91) | 2009 | MEC | USA | African American | 1994 | 1,7 | -8.61% (-15.50% to -1.15%) | 916 | 0.03 | Continuous |
|  |  |  |  | European American |  |  | -2.96% (-14.56% to 10.23%) | 446 | 0.65 |  |
|  |  |  |  | Japanese American |  |  | -11.31% (-21.92% to 0.74%) | 485 | 0.06 |  |
|  |  |  |  | Latino |  |  | -7.69% (-15.48% to 0.82%) | 714 | 0.06 |  |
|  |  |  |  | Native Hawaiian |  |  | -9.06% (-17.55% to 0.30%) | 313 | 0.06 |  |
| Kim (92) | 2011 |  | Korea |  | 2008 | 1 | -10.40% (-15.64% to -4.84%) | 258 | 0.0004 | Categories |
| Wright (93) | 2011 |  | USA |  | 2003 | 1,6,7 | -10.44% (-18.09% to -2.07%) | 770 | 0.02 | Categories |
| Li (94) | 2012 | NHANES | USA |  | 2003 | 1,2,6,11,13,14,15 | -6.90% (-9.30% to -4.44%) | 3713 | 0.001 | Continuous |
| Park (95) | 2012 |  | Korean |  | 2006 | 1 | -8.19% (-10.74% to -5.58%) | 6389 | <0.0001 | Categories |
| Chamie (96) | 2013 |  | USA | White (55%), Hispanic (25.8%) | 2006 | 1,2,5 | -4.88% (-13.76% to 4.92%) | 573 | 0.4 | Continuous |
| Bhindi (97) | 2014 | BioBank | Canada |  | 2012 |  | -4.07% (-7.52% to -0.49%) | 1613 | 0.25 | Categories |
| Bonn (98) | 2016 | STHLM-2 | Sweden |  | 2011 | 1,4,8,10,11,12 | -7.98% (-10.12% to -5.79%) | 13341 | <0.0001 | Continuous |
| Krimpen (45) |  | Krimpen | Netherlands | White |  | 1,2 | -4.23% (-10.82% to 2.86%) | 1661 | 0.24 | IPD |
| PLCO (66) |  | PLCO | USA | White |  | 1,2 | -7.08% (-8.17% to -5.98%) | 33025 | <0.0001 | IPD |
| ProtecT (67) |  | ProtecT | UK | White |  | 1,2 | -6.06% (-7.06% to -5.04%) | 41412 | <0.0001 | IPD |
| Albatross only | | | | | | | | | | |
| Gray (99) | 2004 |  | New Zealand | 44% European | 2001 | 1,2 | Negative | 769 | 0.005 | PSA MD BMI>34 vs BMI<34 |
| Chang (100) | 2008 |  | Korea |  | 2003 | 1 | Negative | 8176 | 0.01 | P for trend |
| Chia (101) | 2009 |  | Singapore | Asian | 2005 |  | Negative | 2410 | 0.001 | P for trend |
| Loeb (102) | 2009 | BLSA | USA | Caucasian | 1958 | 1 | Negative | 994 | 0.06 | PSA regression |
| Chiu (103) | 2011 |  | China |  | 2007 | 1 | Negative | 1612 | 0.02 | PSA regression |
| Liu (104) | 2011 |  | China |  | 2007 | 1 | Negative | 6774 | 0.0001 | PSA regression |
| Wallner (105) | 2011 | OCS | USA |  | 1990 | 1 | Negative | 545 | 0.18 | PSA intercept |
| Gomez-Guerra (106) | 2012 |  | Mexico |  |  | 1 | Negative | 152 | 0.006 | PSA regression |
| Ikuerowo (107) | 2012 |  | Nigeria | Nigerian | 2012 | 1,3 | Positive | 1954 | 0.07 | Obese ≥ 4.0 ng/ml |
| Pater (108) | 2012 |  | USA |  | 2006 | 1,2 | Negative | 767 | 0.03 | PSA regression |
| Yang (109) | 2013 |  | Korea |  | 2010 | 1 | Negative | 20509 | 0.08 | Obese ≥ 4.0 ng/ml |
| Taghavi (110) | 2014 |  | Iran |  |  |  | Positive | 140 | 0.04 | PSA MD BMI>27 vs BMI<27 |
| Adegun (111) | 2015 | ESUTHS | Nigeria | Nigerian | 2014 | 1 | Negative | 140 | 0.41 | Obese ≥ 4.0 ng/ml |
| Zhang (112) | 2016 |  | China |  | 2011 | 1 | Negative | 15296 | 0.06 | PSA regression |
| Yun (113) | 2017 |  | Korea |  | 2010 | 1,17,18 | Negative | 18800 | 0.003 | PSA regression |
| *Year = publication year, Ethnicity = Ethnicity (%) if specified, Mid-year = the mid-year of recruitment for each study (publication data minus 1 year if not reported), Effect estimate = Percentage change in PSA for a 5 kg/m^2^ increase in BMI (meta-analysis) or effect direction (albatross), P value = P value for effect estimate (taken from P for trend for categorical effects, if available), Effect type = original presentation of data (meta-analysis) or to which effect estimate the P value refers (albatross)* | | | | | | | | | | |
| *Variables adjusted: 1 = Age, 2 = Ethnicity, 3 = DRE, 4 = Family History of prostate cancer, 5 = Prostate Cancer Characteristics, 6 = Statins & aspirin, 7 = Diabetes, 8 = Education, 9 = Vasectomy, 10 = Exercise, 11 = Smoking, 12 = Stress, 13 = BPH, 14 = NSAID, 15 = Thiazide, 16 = C-reactive protein, 17 = Neutrophil count, 18 = Neutrophil-lymphocyte ratio, 19 = Fasting blood sugar* | | | | | | | | | | |
| *Study acronyms: BLSA = Baltimore Longitudinal Study of Aging, ESTHER = Epidemiological study on chances of prevention, early detection, ESUTHS = Ekiti State University Teaching Hospital Study, MEC = MultiEthnic Cohort, NHANES = National Health and Nutrition Examination Survey, and treatment optimization of chronic diseases in the elderly, OCS = Olmsted County Study, PLCO = Prostate, Lung, Colorectal, and Ovarian Cancer Screening Trial, ProtecT = Prostate Testing for Cancer and Treatment, SABOR = San Antonio Center for Biomarkers of Risk of prostate carcinoma, SEARCH = Shared equal access regional cancer hospital, STHLM-2 = Stockholm-2* | | | | | | | | | | |

Table S12 Risk of bias of studies examining the association between BMI and PSA

| Author | Overall RoB | Confounding | Selection of Participants | Missing Data | Measurement of Outcome | Measurement of Exposure | Selective Reporting |
| --- | --- | --- | --- | --- | --- | --- | --- |
| Meta-analysis | | | | | | | |
| Baillargeon (83) | Medium | Medium | Low | Low | Medium | Low | Low |
| Freedland (84) | High | Medium | High | Low | Low | Medium | Low |
| Bañez (85) | High | Medium | High | Low | Low | Medium | Low |
| Sohn (86) | High | Medium | High | Low | Low | Medium | Low |
| Ando (87) | High | Medium | High | Low | Low | Medium | Low |
| Price (88) | Medium | Medium | Low | Low | Low | Low | Low |
| Muller (89) | Medium | Medium | Low | Low | Low | Low | Low |
| Park (90) | Medium | Medium | Low | Low | Low | Unclear | Low |
| Waters (91) | High | High | Low | Low | Low | Medium | Low |
| Kim (92) | High | High | Medium | Low | Low | Low | Low |
| Wright (93) | Medium | Medium | Medium | Low | Low | Low | Low |
| Li (94) | Medium | Medium | Medium | Low | Low | Low | Low |
| Park (95) | Medium | Medium | Medium | Low | Low | Low | Low |
| Chamie (96) | Medium | Medium | Medium | Low | Low | Low | Low |
| Bhindi (97) | Medium | Medium | Medium | Low | Low | Low | Low |
| Bonn (98) | Medium | Medium | Low | Low | Low | Low | Low |
| Krimpen (45) | High | Medium | Medium | Medium | Low | Medium | Low |
| PLCO (66) | Medium | Medium | Low | Low | Low | Low | Low |
| ProtecT (67) | High | High | Low | Low | Low | Low | Low |
| Albatross only |  |  |  |  |  |  |  |
| Gray (99) | Medium | Medium | Low | Low | Low | Low | Low |
| Chang (100) | Medium | Medium | Low | Low | Low | Low | Low |
| Chia (101) | High | High | Low | Low | Low | Low | Low |
| Loeb (102) | Medium | Medium | Low | Low | Low | Low | Low |
| Chiu (103) | High | High | Low | Low | Low | Low | Low |
| Liu (104) | Medium | Medium | Low | Low | Low | Low | Low |
| Wallner (105) | Medium | Medium | Low | Low | Low | Low | Low |
| Gomez-Guerra (106) | High | High | Medium | Low | Low | Low | Low |
| Ikuerowo (107) | High | High | Low | Low | Low | Low | Low |
| Pater (108) | Medium | Medium | Low | Low | Low | Low | Low |
| Yang (109) | Medium | Medium | Low | Low | Low | Low | Low |
| Taghavi (110) | High | Unclear | Unclear | Unclear | Unclear | Unclear | Unclear |
| Adegun (111) | Medium | Medium | Medium | Low | Low | Low | Low |
| Zhang (112) | High | Medium | Low | High | Low | Low | Low |
| Yun (113) | Medium | Medium | Low | Low | Low | Low | Low |

Table S13 Meta-regression results for the linear association between BMI and PSA

| Variable | Difference in betas | 95% CI | P value |
| --- | --- | --- | --- |
| Ethnicity (white versus non-white) | -0.014 | -0.044 to 0.016 | 0.33 |
| Risk of bias (high versus medium risk) | -0.003 | -0.029 to 0.024 | 0.84 |
| BMI mean of study | 0.002 | -0.007 to 0.010 | 0.67 |
| Mid-year of study recruitment | -0.001 | -0.003 to 0.002 | 0.47 |

Table S14 Summary BMI and log-PSA values for normal, overweight and obese BMI categories

| Author/Study | Normal weight (<25 kg/m^2^) | | | Overweight (25-29.9 kg/m^2^) | | | Obese (≥30 kg/m^2^) | | |
| --- | --- | --- | --- | --- | --- | --- | --- | --- | --- |
|  | Mean BMI  (kg/m^2^) | Mean Log-PSA (SD) | N | Mean BMI  (kg/m^2^) | Mean Log-PSA (SD) | N | Mean BMI  (kg/m^2^) | Mean Log-PSA (SD) | N |
| Baillargeon (83) | 21.2 | -0.41 (0.92) | 519 | 27.8 | -0.62 (1.07) | 1318 | 34.6 | -0.49 (0.84) | 933 |
| Freedland (84) | 21.8 | 1.57 (0.89) | 397 | 27.7 | 1.54 (0.90) | 684 | 33.5 | 1.56 (0.90) | 333 |
| Bañez (85) (Duke) | 22.6 | 1.68 (0.66) | 452 | 27.6 | 1.70 (0.63) | 972 | 32.7 | 1.54 (0.65) | 550 |
| Bañez (85) (Johns Hopkins) | 22.7 | 1.60 (0.59) | 2982 | 27.4 | 1.60 (0.59) | 5661 | 32 | 1.55 (0.60) | 1644 |
| Bañez (85) (SEARCH) | 21.8 | 1.90 (0.56) | 357 | 27.7 | 1.81 (0.57) | 611 | 33.6 | 1.76 (0.58) | 405 |
| Sohn (86) | 20.9 | -0.33 (0.98) | 9841 | 24.9 | -0.28 (0.88) | 14215 | 28.8 | -0.47 (0.99) | 2686 |
| Ando (87) | 22.2 | -0.18 (0.62) | 1701 | 27 | -0.21 (0.58) | 1309 | 30.8 | -0.40 (0.65) | 147 |
| Price (88) | 21.7 | -0.01 (0.75) | 144 | 27.7 | -0.03 (0.72) | 247 | 33.7 | -0.23 (0.78) | 144 |
| Muller (89) | 23 | -0.12 (0.82) | 178 | 27.5 | -0.16 (0.86) | 408 | 31.9 | -0.27 (0.90) | 191 |
| Park (90) | 22.7 | -0.14 (0.76) | 22255 | 26.9 | -0.19 (0.76) | 15160 | 31 | -0.31 (0.66) | 995 |
| Kim (92) | 19.1 | 1.03 (0.52) | 112 | 25.1 | 0.84 (0.58) | 85 | 30.6 | 0.82 (0.52) | 61 |
| Wright (93) | 22.6 | 0.17 (0.82) | 219 | 27.4 | 0.12 (0.88) | 363 | 32.2 | -0.06 (0.95) | 188 |
| Park (95) | 22.9 | -0.20 (0.49) | 3604 | 25.9 | -0.21 (0.44) | 1621 | 28.2 | -0.34 (0.57) | 1164 |
| Bhindi (97) | 22.7 | 1.70 (0.46) | 475 | 27.4 | 1.71 (0.48) | 809 | 31.9 | 1.61 (0.51) | 329 |
| Lacher (114) | 22.4 | -0.02 (0.65) | 702 | 27.6 | -0.13 (0.66) | 1315 | 33.1 | -0.30 (0.69) | 1152 |
| Bonn (98) | 21.8 | NK | 5990 | 27.5 | NK | 7688 | 32.2 | NK | 1788 |
| Krimpen (45) | 23.2 | 0.32 (0.88) | 612 | 27.0 | 0.25 (0.90) | 889 | 31.9 | 0.19 (0.92) | 160 |
| PLCO (66) | 23.1 | 0.23 (0.88) | 8483 | 27.2 | 0.18 (0.86) | 16640 | 33.3 | 0.05 (0.90) | 7902 |
| ProtecT (67) | 23.1 | 0.15 (0.89) | 10885 | 27.3 | 0.12 (0.91) | 21103 | 33.1 | 0.02 (0.92) | 9424 |
| *PLCO = Prostate, Lung, Colorectal and Ovarian Cancer Screening Trial, ProtecT = Prostate Testing for Cancer and Treatment study, NK = Not Known*  *Note: BMI values for the IPD are taken from the Continuous imputation of BMI, whereas the number of cases and controls are taken from the Categorical imputation of BMI* | | | | | | | | | |

Table S15 Results for the Categorical analysis of the association between BMI and PSA

| Author/Study | Percentage PSA Mean Difference | |
| --- | --- | --- |
|  | Overweight vs normal (95% CI) | Obese vs normal (95% CI) |
| Baillargeon (83) | -19.05% (-27.09% to -10.13%) | -7.79% (-15.98% to 1.20%) |
| Freedland (84) | -3.45% (-13.61% to 7.91%) | -1.59% (-13.66% to 12.16%) |
| Bañez (85) (Duke) | 2.15% (-4.90% to 9.72%) | -12.66% (-19.48% to -5.27%) |
| Bañez (85) (Johns Hopkins) | -0.41% (-2.98% to 2.23%) | -4.63% (-7.99% to -1.15%) |
| Bañez (85) (SEARCH) | -8.98% (-15.47% to -2.00%) | -13.28% (-20.00% to -5.98%) |
| Sohn (86) | 6.03% (3.54% to 8.57%) | -12.92% (-16.48% to -9.20%) |
| Ando (87) | -2.93% (-7.04% to 1.36%) | -19.71% (-27.66% to -10.88%) |
| Price (88) | -2.03% (-15.70% to 13.86%) | -19.82% (-32.75% to -4.40%) |
| Muller (89) | -4.49% (-17.74% to 10.89%) | -14.61% (-28.39% to 1.83%) |
| Park (90) | -4.98% (-6.46% to -3.48%) | -16.43% (-20.34% to -12.32%) |
| Kim (92) | -17.17% (-29.05% to -3.31%) | -18.42% (-30.61% to -4.08%) |
| Wright (93) | -4.24% (-17.07% to 10.58%) | -20.34% (-32.92% to -5.39%) |
| Park (95) | -1.33% (-4.03% to 1.45%) | -12.89% (-15.78% to -9.90%) |
| Bhindi (97) | 0.55% (-4.73% to 6.11%) | -8.73% (-14.68% to -2.36%) |
| Lacher (114) | -10.15% (-15.38% to -4.59%) | -24.22% (-28.88% to -19.26%) |
| Bonn (98) | -3.55% (-6.89% to -.09%) | -13.67% (-18.92% to -8.08%) |
| Krimpen (45) | -5.03% (-13.19% to 3.89%) | -6.85% (-20.00% to 8.46%) |
| PLCO (66) | -3.50% (-5.75% to -1.19%) | -12.75% (-15.15% to -10.29%) |
| ProtecT (67) | -3.59% (-5.58% to -1.57%) | -11.07% (-13.22% to -8.86%) |
| *PLCO = Prostate, Lung, Colorectal and Ovarian Cancer Screening Trial, ProtecT = Prostate Testing for Cancer and Treatment study* | | |

### References

1. Severson RK, Grove JS, Nomura a M, Stemmermann GN. Body mass and prostatic cancer: a prospective study. BMJ. 1988;297(6650):713–5.

2. Mills PK, Beeson WL, Phillips RL, Fraser GE. Cohort study of diet, lifestyle, and prostate cancer in adventist men. Cancer [Internet]. 1989;64(3):598–604. Available from: http://onlinelibrary.wiley.com/doi/10.1002/1097-0142(19890801)64:3%3C598::AID-CNCR2820640306%3E3.0.CO;2-6/abstract%5Cnhttp://onlinelibrary.wiley.com/doi/10.1002/1097-0142(19890801)64:3%3C598::AID-CNCR2820640306%3E3.0.CO;2-6/abstract%5Cnhttp://onlinelibrary.wiley.co

3. Andersson S-O, Wolk A, Bergstrom R, Adami H-O, Engholm G, Englund A, et al. Body Size and Prostate Cancer: A 20-Year Follow-up Study Among 135006 Swedish Construction Workers. JNCI J Natl Cancer Inst [Internet]. 1997;89(5):385–9. Available from: http://jnci.oxfordjournals.org/cgi/doi/10.1093/jnci/89.5.385

4. Cerhan JR, Torner JC, Lynch CF, Rubenstein LM, Lemke JH, Cohen MB, et al. Association of smoking, body mass, and physical activity with risk of prostate cancer in the Iowa 65+ Rural Health Study (United States). Cancer Causes Control [Internet]. 1997;8(2):229–38. Available from: http://www.ncbi.nlm.nih.gov/pubmed/9134247

5. Giovannucci E, Rimm EB, Stampfer MJ, Colditz GA, Willett WC. Height, body weight, and risk of prostate cancer. Cancer Epidemiol Biomarkers Prev [Internet]. 1997;6(8):557–63. Available from: http://www.ncbi.nlm.nih.gov/pubmed/9264267%5Cnhttp://cebp.aacrjournals.org/content/6/8/557.full.pdf

6. Lund Nilsen TI, Vatten LJ. Anthropometry and prostate cancer risk: A prospective study of 22,248 Norwegian men. Cancer Causes Control [Internet]. 1999;10(4):269–75. Available from: http://www.ncbi.nlm.nih.gov/entrez/query.fcgi?cmd=Retrieve&db=PubMed&dopt=Citation&list_uids=10482485

7. Schuurman AG, Goldbohm RA, Dorant E, van den Brandt PA. Anthropometry in relation to prostate cancer risk in the Netherlands Cohort Study. Am J Epidemiol [Internet]. 2000;151(6):541–9. Available from: http://www.ncbi.nlm.nih.gov/entrez/query.fcgi?cmd=Retrieve&db=PubMed&dopt=Citation&list_uids=10733035

8. Lee IM, Sesso HD, Paffenbarger RS. A prospective cohort study of physical activity and body size in relation to prostate cancer risk (United States). Cancer Causes Control [Internet]. 2001;12(2):187–93. Available from: http://dx.doi.org/10.1023/A:1008952528771

9. Engeland A, Tretli S, Bjørge T. Height, body mass index, and prostate cancer: a follow-up of 950000 Norwegian men. Br J Cancer [Internet]. 2003;89(7):1237–42. Available from: http://www.nature.com/doifinder/10.1038/sj.bjc.6601801

10. Jonssoni F, Wolk A, Pedersen NL, Lichtenstein P, Terry P, Ahlbom A, et al. Obesity and hormone-dependent tumors: Cohort and co-twin control studies based on the Swedish Twin Registry. Int J Cancer [Internet]. 2003;106(4):594–9. Available from: http://www.ncbi.nlm.nih.gov/pubmed/12845658

11. Kuriyama S, Tsubono Y, Hozawa A, Shimazu T, Suzuki Y, Koizumi Y, et al. Obesity and risk of cancer in Japan. Int J Cancer. 2005;113(1):148–57.

12. Oh SW, Yoon YS, Shin SA. Effects of excess weight on cancer incidences depending on cancer sites and histologic findings among men: Korea National Health Insurance Corporation study. J Clin Oncol [Internet]. 2005;23(21):4742–54. Available from: http://www.jco.org/cgi/doi/10.1200/JCO.2005.11.726

13. Håheim LL, Wisløff TF, Holme I, Nafstad P. Metabolic syndrome predicts prostate cancer in a cohort of middle-aged Norwegian men followed for 27 years. Am J Epidemiol. 2006;164(8):769–74.

14. Kurahashi N, Iwasaki M, Sasazuki S, Otani T, Inoue M, Tsugane S. Association of body mass index and height with risk of prostate cancer among middle-aged Japanese men. Br J Cancer [Internet]. 2006;94(5):740–2. Available from: http://www.pubmedcentral.nih.gov/articlerender.fcgi?artid=2361195&tool=pmcentrez&rendertype=abstract

15. Lukanova A, Björ O, Kaaks R, Lenner P, Lindahl B, Hallmans G, et al. Body mass index and cancer: Results from the Northern Sweden Health and Disease Cohort. Int J Cancer [Internet]. 2006;118(2):458–66. Available from: http://www.ncbi.nlm.nih.gov/pubmed/16049963

16. Lundqvist E, Kaprio J, Verkasalo PK, Pukkala E, Koskenvuo M, Söderberg KC, et al. Co-twin control and cohort analyses of body mass index and height in relation to breast, prostate, ovarian, corpus uteri, colon and rectal cancer among Swedish and Finnish twins. Int J Cancer [Internet]. 2007;121(4):810–8. Available from: http://www.ncbi.nlm.nih.gov/pubmed/17455257

17. Rodriguez C, Freedland SJ, Deka A, Jacobs EJ, McCullough ML, Patel A V, et al. Body mass index, weight change, and risk of prostate cancer in the Cancer Prevention Study II Nutrition Cohort. Cancer Epidemiol Biomarkers Prev. 2007;16(1):63–9.

18. Wright ME, Chang S-C, Schatzkin A, Albanes D, Kipnis V, Mouw T, et al. Prospective study of adiposity and weight change in relation to prostate cancer incidence and mortality. Cancer [Internet]. 2007;109(4):675–84. Available from: http://www.ncbi.nlm.nih.gov/pubmed/17211863

19. Hernandez BY, Park S-Y, Wilkens LR, Henderson BE, Kolonel LN. Relationship of body mass, height, and weight gain to prostate cancer risk in the multiethnic cohort. Cancer Epidemiol Biomarkers Prev [Internet]. 2009;18(9):2413–21. Available from: http://www.pubmedcentral.nih.gov/articlerender.fcgi?artid=2742565&tool=pmcentrez&rendertype=abstract

20. Wallström P, Bjartell a, Gullberg B, Olsson H, Wirfält E. A prospective Swedish study on body size, body composition, diabetes, and prostate cancer risk. Br J Cancer [Internet]. 2009;100(11):1799–805. Available from: http://www.pubmedcentral.nih.gov/articlerender.fcgi?artid=2695694&tool=pmcentrez&rendertype=abstract

21. Burton A, Martin R, Galobardes B, Davey Smith G, Jeffreys M. Young adulthood body mass index and risk of cancer in later adulthood: historical cohort study. Cancer Causes Control. 2010;21(12):2069–77.

22. Stocks T, Hergens M-P, Englund A, Ye W, Stattin P. Blood pressure, body size and prostate cancer risk in the Swedish Construction Workers cohort. Int J Cancer [Internet]. 2010;127(7):1660–8. Available from: http://www.ncbi.nlm.nih.gov/pubmed/20087861

23. Bassett JK, Severi G, Baglietto L, MacInnis RJ, Hoang HN, Hopper JL, et al. Weight change and prostate cancer incidence and mortality. Int J Cancer. 2012;131(7):1711–9.

24. Häggström C, Stocks T, Ulmert D, Bjørge T, Ulmer H, Hallmans G, et al. Prospective study on metabolic factors and risk of prostate cancer. Cancer. 2012;118(24):6199–206.

25. Shafique K, McLoone P, Qureshi K, Leung H, Hart C, Morrison DS. Cholesterol and the risk of grade-specific prostate cancer incidence: evidence from two large prospective cohort studies with up to 37 years&apos; follow up. BMC Cancer [Internet]. 2012;12:25. Available from: http://eutils.ncbi.nlm.nih.gov/entrez/eutils/elink.fcgi?dbfrom=pubmed&amp;id=22260413&amp;retmode=ref&amp;cmd=prlinks

26. Rao GA, Mann JR, Bottai M, Uemura H, Burch JB, Bennett CL, et al. Angiotensin receptor blockers and risk of prostate cancer among united states veterans. J Clin Pharmacol. 2013;53(7):773–8.

27. Bhaskaran K, Douglas I, Forbes H, dos-Santos-Silva I, Leon DA, Smeeth L. Body-mass index and risk of 22 specific cancers: a population-based cohort study of 5·24 million UK adults. Lancet [Internet]. 2014;384(9945):755–65. Available from: http://dx.doi.org/10.1016/S0140-6736(14)60892-8

28. Møller H, Roswall N, Van Hemelrijck M, Larsen SB, Cuzick J, Holmberg L, et al. Prostate cancer incidence, clinical stage and survival in relation to obesity: A prospective cohort study in Denmark. Int J Cancer [Internet]. 2014;1947:1–8. Available from: http://www.ncbi.nlm.nih.gov/pubmed/25264293

29. Choi JB, Moon HW, Park YH, Bae WJ, Cho HJ, Hong SH, et al. The impact of diabetes on the risk of prostate cancer development according to body mass index: A 10-year nationwide cohort study. J Cancer. 2016;7(14):2061–6.

30. Perez-Cornago A, Appleby PN, Pischon T, Tsilidis KK, Tjønneland A, Olsen A, et al. Tall height and obesity are associated with an increased risk of aggressive prostate cancer: Results from the EPIC cohort study. BMC Med. 2017;15(1).

31. Heikkila R, Aho K, Heliovaara M, Hakama M, Marniemi J, Reunanen A, et al. Serum testosterone and sex hormone-binding globulin concentrations and the risk of prostate carcinoma: a longitudinal study. Cancer [Internet]. 1999;86(2):312–5. Available from: http://www.ncbi.nlm.nih.gov/entrez/query.fcgi?cmd=Retrieve&db=PubMed&dopt=Citation&list_uids=10421267

32. Giles GG, Severi G, English DR, McCredie MRE, MacInnis R, Boyle P, et al. Early growth, adult body size and prostate cancer risk. Int J Cancer. 2003;103(2):241–5.

33. Jian L, Shen ZJ, Lee AH, Binns CW. Moderate physical activity and prostate cancer risk: A case-control study in china. Eur J Epidemiol. 2005;20(2):155–60.

34. LIU X, RYBICKI BA, CASEY G, WITTE JS. Relationship Between Body Size and Prostate Cancer in a Sibling Based Case-Control Study. J Urol [Internet]. 2005;174(6):2169–73. Available from: http://linkinghub.elsevier.com/retrieve/pii/S0022534701689390

35. Porter MP, Stanford JL. Obesity and the risk of prostate cancer. Prostate. 2005;62(April 2004):316–21.

36. Baillargeon J, Platz EA, Rose DP, Pollock BH, Ankerst DP, Haffner S, et al. Obesity, adipokines, and prostate cancer in a prospective population-based study. Cancer Epidemiol Biomarkers Prev. 2006;15(7):1331–5.

37. Máchová L, Čížek L, Horáková D, Koutná J, Lorenc J, Janoutová G, et al. Association between obesity and cancer incidence in the population of the District Sumperk, Czech Republic. Onkologie. 2007;30(11):538–42.

38. Albanes D, Weinstein SJ, Wright ME, Männistö S, Limburg PJ, Snyder K, et al. Serum insulin, glucose, indices of insulin resistance, and risk of prostate cancer. J Natl Cancer Inst. 2009;101(18):1272–9.

39. Farhat GN, Taioli E, Cauley JA, Zmuda JM, Orwoll E, Bauer DC, et al. The association of bone mineral density with prostate cancer risk in the osteoporotic fractures in men (MrOS) study. Cancer Epidemiol Biomarkers Prev. 2009;18(1):148–54.

40. Stark JR, Li H, Kraft P, Kurth T, Giovannucci EL, Stampfer MJ, et al. Circulating prediagnostic interleukin-6 and C-reactive protein and prostate cancer incidence and mortality. Int J Cancer [Internet]. 2009;124(11):2683–9. Available from: http://doi.wiley.com/10.1002/ijc.24241

41. Mori M, Masumori N, Fukuta F, Nagata Y, Sonoda T, Miyanaga N, et al. Weight gain and family history of prostate or breast cancers as risk factors for prostate cancer: results of a case-control study in Japan. Asian Pac J Cancer Prev [Internet]. 2011;12(3):743–7. Available from: http://www.ncbi.nlm.nih.gov/pubmed/21627376

42. Bhavsar NA, Bream JH, Meeker AK, Drake CG, Peskoe SB, Dabitao D, et al. A peripheral circulating TH1 cytokine profile is inversely associated with prostate cancer risk in CLUE II. Cancer Epidemiol Biomarkers Prev. 2014;23(11):2561–7.

43. Stefani E De, Boffetta PL, Ronco A, Deneo-Pellegrini H. Meat Consumption, Related Nutrients, Obesity and Risk of Prostate Cancer: a Case-Control Study in Uruguay. Asian Pac J Cancer Prev. 2016;17(4):1937–45.

44. Kunutsor SK, Laukkanen JA. Gamma-glutamyltransferase and risk of prostate cancer: Findings from the KIHD prospective cohort study. Int J Cancer. 2017;140(4):818–24.

45. Blanker MH, Groeneveld FPMJ, Prins A, Bernsen RMD, Bohnen AM, Bosch JLHR. Strong effects of definition and nonresponse bias on prevalence rates of clinical benign prostatic hyperplasia: The Krimpen study of male urogenital tract problems and general health status. BJU Int. 2000;85(6):665–71.

46. Whittemore AS, Kolonel LN, Wu AH, John EM, Gallagher RP, Howe GR, et al. Prostate cancer in relation to diet, physical activity, and body size in blacks, whites, and Asians in the United States and Canada. J Natl Cancer Inst [Internet]. 1995;87(9):652–61. Available from: internal-pdf://72.80.119.144/Whittemore-1995-Prostate cancer in relation to.pdf%5Cnhttp://jnci.oxfordjournals.org/content/87/9/652.full.pdf

47. Andersson SO, Baron J, Bergström R, Lindgren C, Wolk a, Adami HO. Lifestyle factors and prostate cancer risk: a case-control study in Sweden. Cancer Epidemiol Biomarkers Prev. 1996;5(7):509–13.

48. Lagiou P, Signorello LB, Trichopoulos D, Tzonou a, Trichopoulou a, Mantzoros CS. Leptin in relation to prostate cancer and benign prostatic hyperplasia. Int J Cancer [Internet]. 1998;76(1):25–8. Available from: http://www.ncbi.nlm.nih.gov/pubmed/9533757

49. Hsieh CC, Thanos A, Mitropoulos D, Deliveliotis C, Mantzoros CS, Trichopoulos D. Risk factors for prostate cancer: a case-control study in Greece. Int J Cancer [Internet]. 1999;80(5):699–703. Available from: http://ovidsp.ovid.com/ovidweb.cgi?T=JS&CSC=Y&NEWS=N&PAGE=fulltext&D=med4&AN=10048970

50. Villeneuve PJ, Johnson KC, Kreiger N, Mao Y, Paulse B, Dewar R, et al. Risk factors for prostate cancer: Results from the Canadian National Enhanced Cancer Surveillance System. Cancer Causes Control. 1999;10(5):355–67.

51. Hsing AW, Chua S, Gao Y, Gentzschein E, Chang L, Deng J, et al. Prostate Cancer Risk and Serum Levels of Insulin and Leptin : a Population- Based Study. 2001;93(10):783–9.

52. Sharpe CR, Siemiatycki J. Joint effects of smoking and body mass index on prostate cancer risk. Epidemiology [Internet]. 2001;12(5):546–51. Available from: http://www.ncbi.nlm.nih.gov/pubmed/11505174

53. Cui Y, Winton MI, Zhang ZF, Rainey C, Marshall J, De Kernion JB, et al. Dietary boron intake and prostate cancer risk. Oncol Rep. 2004;11(4):887–92.

54. Dal Maso L, Zucchetto a, La Vecchia C, Montella M, Conti E, Canzonieri V, et al. Prostate cancer and body size at different ages: an Italian multicentre case-control study. Br J Cancer [Internet]. 2004;90(11):2176–80. Available from: http://www.pubmedcentral.nih.gov/articlerender.fcgi?artid=2409495&tool=pmcentrez&rendertype=abstract

55. Friedenreich CM, McGregor SE, Courneya KS, Angyalfi SJ, Elliott FG. Case-control study of anthropometric measures and prostate cancer risk. Int J Cancer [Internet]. 2004;110(2):278–83. Available from: http://www.ncbi.nlm.nih.gov/pubmed/15069694

56. Bradbury BD, Wilk JB, Kaye JA. Obesity and the risk of prostate cancer (United States). Cancer Causes Control [Internet]. 2005;16(6):637–41. Available from: http://www.embase.com/search/results?subaction=viewrecord&from=export&id=L41110953%5Cnhttp://dx.doi.org/10.1007/s10552-005-0383-6%5Cnhttp://sfx.library.uu.nl/sfx?sid=EMBASE&issn=09575243&id=doi:10.1007/s10552-005-0383-6&atitle=Obesity+and+the+risk+of+prostate

57. Gallus S, Foschi R, Talamini R, Altieri A, Negri E, Franceschi S, et al. Risk Factors for Prostate Cancer in Men Aged Less Than 60 Years: A Case-Control Study from Italy. Urology [Internet]. 2007;70(6):1121–6. Available from: http://www.ncbi.nlm.nih.gov/pubmed/18158031

58. Chamie K, DeVere White RW, Lee D, Ok J-H, Ellison LM. Agent Orange exposure, Vietnam War veterans, and the risk of prostate cancer. Cancer [Internet]. 2008;113(9):2464–70. Available from: http://www.ncbi.nlm.nih.gov/pubmed/18666213

59. Chia SE, Wong KY, Cheng C, Lau W, Tan PH. Sun exposure and the risk of prostate cancer in the Singapore Prostate Cancer Study: a case-control study. Asian PacJ Cancer Prev [Internet]. 2012;13(7):3179–85. Available from: http://www.ncbi.nlm.nih.gov/pubmed/22994730

60. Fowke JH, Motley SS, Concepcion RS, Penson DF, Barocas DA. Obesity, body composition, and prostate cancer. BMC Cancer [Internet]. 2012;12(1):23. Available from: http://bmccancer.biomedcentral.com/articles/10.1186/1471-2407-12-23

61. pinnacle, Nemesure B, Wu S-Y-., Hennis A, Leske MC. Central Adiposity and Prostate Cancer in a Black Population. Cancer Epidemiol Biomarkers Prev [Internet]. 2012;21(5):851–8. Available from: http://www.ncbi.nlm.nih.gov/pubmed/22402288%5Cnhttp://cebp.aacrjournals.org/cgi/doi/10.1158/1055-9965.EPI-12-0071

62. Yaturu S, Zdunek S, Youngberg B. Vitamin d levels in subjects with prostate cancer compared to age-matched controls. Prostate Cancer [Internet]. 2012;2012:524206. Available from: http://www.pubmedcentral.nih.gov/articlerender.fcgi?artid=3530178&tool=pmcentrez&rendertype=abstract

63. Möller E, Adami H-O, Mucci L a, Lundholm C, Bellocco R, Johansson J-E, et al. Lifetime body size and prostate cancer risk in a population-based case-control study in Sweden. Cancer Causes Control [Internet]. 2013;24(12):2143–55. Available from: http://www.ncbi.nlm.nih.gov/pubmed/24048969

64. Salem S, Hosseini M, Allameh F, Babakoohi S, Mehrsai A, Pourmand G. Serum calcium concentration and prostate cancer risk: a multicenter study. Nutr Cancer [Internet]. 2013;65(7):961–8. Available from: http://www.tandfonline.com/doi/abs/10.1080/01635581.2013.806936#.VuRf8fkrLIU

65. Boehm K, Sun M, Larcher A, Blanc-Lapierre A, Schiffmann J, Graefen M, et al. Waist circumference, waist-hip ratio, body mass index, and prostate cancer risk: Results from the North-American case-control study Prostate Cancer & Environment Study. Urol Oncol Semin Orig Investig [Internet]. 2015;33(11):494.e1-494.e7. Available from: http://www.embase.com/search/results?subaction=viewrecord&from=export&id=L605758449%5Cnhttp://dx.doi.org/10.1016/j.urolonc.2015.07.006%5Cnhttp://hz9pj6fe4t.search.serialssolutions.com.proxy.cc.uic.edu/?sid=EMBASE&sid=EMBASE&issn=18732496&id=doi:10.1016%2Fj.ur

66. Andriole GL, Crawford ED, Grubb 3rd RL, Buys SS, Chia D, Church TR, et al. Prostate cancer screening in the randomized Prostate, Lung, Colorectal, and Ovarian Cancer Screening Trial: mortality results after 13 years of follow-up. J Natl Cancer Inst [Internet]. 2012;104(2):125–32. Available from: http://www.ncbi.nlm.nih.gov/pubmed/22228146%5Cnhttp://jnci.oxfordjournals.org/content/104/2/125.full.pdf

67. Lane JA, Donovan JL, Davis M, Walsh E, Dedman D, Down L, et al. Active monitoring, radical prostatectomy, or radiotherapy for localised prostate cancer: Study design and diagnostic and baseline results of the ProtecT randomised phase 3 trial. Lancet Oncol. 2014;15(10):1109–18.

68. Le Marchand L, Kolonel LN, Wilkens LR, Myers BC, Hirohata T. Animal fat consumption and prostate cancer: a prospective study in Hawaii. Epidemiology [Internet]. 1994;5(3):276–82. Available from: http://www.ncbi.nlm.nih.gov/pubmed/8038241

69. Veierød MB, Laake P, Thelle DS. Dietary fat intake and risk of prostate cancer: a prospective study of 25,708 Norwegian men. Int J Cancer [Internet]. 1997;73(5):634–8. Available from: http://www.ncbi.nlm.nih.gov/pubmed/9398038

70. Habel LA, Van Den Eeden SK, Friedman GD. Body size, age at shaving initiation, and prostate cancer in a large, multiracial cohort. Prostate [Internet]. 2000;43(2):136–43. Available from: http://www.ncbi.nlm.nih.gov/pubmed/10754529

71. Hsing a W, Deng J, Sesterhenn I a, Mostofi FK, Stanczyk FZ, Benichou J, et al. Body size and prostate cancer: a population-based case-control study in China. Cancer Epidemiol Biomarkers Prev. 2000;9(12):1335–41.

72. Putnam SD, Cerhan JR, Parker AS, Bianchi GD, Wallace RB, Cantor KP, et al. Lifestyle and anthropometric risk factors for prostate cancer in a cohort of Iowa men. Ann Epidemiol. 2000;10(6):361–9.

73. Pan SY, Johnson KC, Ugnat AM, Wen SW, Mao Y. Association of Obesity and Cancer Risk in Canada. Am J Epidemiol. 2004;159(3):259–68.

74. Cox B, Sneyd MJ, Paul C, Skegg DCG. Risk factors for prostate cancer: A national case-control study. Int J Cancer [Internet]. 2006;119(7):1690–4. Available from: http://doi.wiley.com/10.1002/ijc.22022

75. Littman AJ, White E, Kristal AR. Anthropometrics and prostate cancer risk. Am J Epidemiol [Internet]. 2007;165(11):1271–9. Available from: http://www.ncbi.nlm.nih.gov/pubmed/17395597

76. Attner B, Landin-Olsson M, Lithman T, Noreen D, Olsson H. Cancer among patients with diabetes, obesity and abnormal blood lipids: a population-based register study in Sweden. Cancer Causes Control [Internet]. 2012;23(5):769–77. Available from: http://link.springer.com/article/10.1007%2Fs10552-012-9946-5%5Cnhttp://download.springer.com/static/pdf/443/art%253A10.1007%252Fs10552-012-9946-5.pdf?originUrl=http%3A%2F%2Flink.springer.com%2Farticle%2F10.1007%2Fs10552-012-9946-5&token2=exp=1455626637~acl=

77. Harding JL, Shaw JE, Anstey KJ, Adams R, Balkau B, Brennan-Olsen SL, et al. Comparison of anthropometric measures as predictors of cancer incidence: A pooled collaborative analysis of 11 Australian cohorts. Int J Cancer. 2015;137(7):1699–708.

78. Heir T, Falk RS, Robsahm TE, Sandvik L, Erikssen J, Tretli S. Cholesterol and prostate cancer risk: A long-term prospective cohort study. BMC Cancer. 2016;16(1).

79. Park S-Y, Haiman CA, Cheng I, Park SL, Wilkens LR, Kolonel LN, et al. Racial/ethnic differences in lifestyle-related factors and prostate cancer risk: the Multiethnic Cohort Study. Cancer Causes Control [Internet]. 2015;26(10):1507–15. Available from: http://www.ncbi.nlm.nih.gov/pubmed/26243447%5Cnhttp://www.pubmedcentral.nih.gov/articlerender.fcgi?artid=PMC4567936

80. Discacciati A, Orsini N, Andersson S-O, Andrén O, Johansson J-E, Wolk A. Body mass index in early and middle-late adulthood and risk of localised, advanced and fatal prostate cancer: a population-based prospective study. Br J Cancer [Internet]. 2011;105(7):1061–8. Available from: http://dx.doi.org/10.1038/bjc.2011.319

81. Robinson WR, Stevens J, Gammon MD, John EM. Obesity before age 30 years and risk of advanced prostate cancer. Am J Epidemiol [Internet]. 2005;161(12):1107–14. Available from: http://aje.oupjournals.org/cgi/doi/10.1093/aje/kwi150

82. Geybels MS, Verhage BAJ, Arts ICW, Van Schooten FJ, Alexandra Goldbohm R, Van Den Brandt PA. Dietary flavonoid intake, black tea consumption, and risk of overall and advanced stage prostate cancer. Am J Epidemiol. 2013;177(12):1388–98.

83. Baillargeon J, Pollock BH, Kristal AR, Bradshaw P, Hernandez J, Basler J, et al. The association of body mass index and prostate-specific antigen in a population-based study. Cancer. 2005;103(5):1092–5.

84. Freedland SJ, Platz EA, Presti JC, Aronson WJ, Amling CL, Kane CJ, et al. Obesity, serum prostate specific antigen and prostate size: Implications for prostate cancer detection. J Urol. 2006;175(2):500–4.

85. Bañez LL, Hamilton RJ, Partin AW, Vollmer RT, Sun L, Rodriguez C, et al. Obesity-related plasma hemodilution and PSA concentration among men with prostate cancer. Jama [Internet]. 2007;298(19):2275–80. Available from: http://jama.jamanetwork.com/article.aspx?articleid=209508%5Cnhttp://jama.jamanetwork.com/article.aspx?doi=10.1001/jama.298.19.2275%5Cnhttp://www.ncbi.nlm.nih.gov/pubmed/18029831

86. J.C. S, M.S. L, H.S. C, C.H. P. The association of body mass index and prostate-specific antigen [Internet]. Vol. 48, Korean Journal of Urology. 2007. p. 1121–4. Available from: http://ovidsp.ovid.com/ovidweb.cgi?T=JS&PAGE=reference&D=emed11&NEWS=N&AN=350200835

87. Ando R, Nagaya T, Hashimoto Y, Suzuki S, Itoh Y, Umemoto Y, et al. Inverse relationship between obesity and serum prostate-specific antigen level in healthy Japanese men: a hospital-based cross-sectional survey, 2004-2006. Urology. 2008;72(3):561–5.

88. Price MM, Hamilton RJ, Robertson CN, Butts MC, Freedland SJ. Body Mass Index, Prostate-Specific Antigen, and Digital Rectal Examination Findings Among Participants in a Prostate Cancer Screening Clinic. Urology [Internet]. 2008;71(5):787–91. Available from: http://www.ncbi.nlm.nih.gov/pubmed/18267334

89. Muller H, Raum E, Rothenbacher D, Stegmaier C, Brenner H, Mu H. Association of Diabetes and Body Mass Index with Levels of Prostate-Specific Antigen: Implications for Correction of Prostate-Specific Antigen Cutoff Values? Cancer Epidemiol Biomarkers Prev [Internet]. 2009;18(5):1350–6. Available from: http://cebp.aacrjournals.org/cgi/content/abstract/18/5/1350

90. Park J-H, Cho B-L, Kwon H-T, Lee C-M, Han H-J. Effect of body mass index and waist circumference on prostate specific antigen and prostate volume in a generally healthy Korean population. J Urol [Internet]. 2009;182(1):106-10-1. Available from: http://www.ncbi.nlm.nih.gov/pubmed/19450837

91. Waters K, Henderson B, Stram D, Wan P, Kolonel L, Haiman C. Association of Diabetes With Prostate Cancer Risk in the Multiethnic Cohort. Am J Epidemiol [Internet]. 2009; Available from: http://aje.oxfordjournals.org/cgi/content/full/kwp003v1%5Cnpapers2://publication/doi/10.1093/aje/kwp003

92. Kim JM, Song PH, Kim HT, Moon KH. Effect of obesity on prostate-specific antigen, prostate volume, and international prostate symptom score in patients with benign prostatic hyperplasia. Korean J Urol. 2011;52(6):401–5.

93. Wright JL, Lin DW, Stanford JL. The effect of demographic and clinical factors on the relationship between BMI and PSA levels. Prostate. 2011;71(15):1631–7.

94. Li J, Thompson T, Joseph DA, Master VA. Association Between Smoking Status, and Free, Total and Percent Free Prostate Specific Antigen. J Urol [Internet]. 2012;187(4):1228–33. Available from: http://linkinghub.elsevier.com/retrieve/pii/S0022534711057648

95. Park S-G, Choi H-C, Cho B, Kwon Y-M, Kwon H-T, Park J-H. Effect of central obesity on prostate specific antigen measured by computerized tomography: related markers and prostate volume. J Urol [Internet]. 2012;187(5):1589–93. Available from: http://www.ncbi.nlm.nih.gov/pubmed/22425083

96. Chamie K, Oberfoell S, Kwan L, Labo J, Wei JT, Litwin MS. Body mass index and prostate cancer severity: Do obese men harbor more aggressive disease on prostate biopsy? Urology [Internet]. 2013;81(5):949–55. Available from: http://dx.doi.org/10.1016/j.urology.2013.01.021

97. Bhindi B, Margel D, Trottier G, Hamilton RJ, Kulkarni GS, Hersey KM, et al. Obesity is associated with larger prostate volume but not with worse urinary symptoms: Analysis of a large multiethnic cohort. Urology [Internet]. 2014;83(1):81–7. Available from: http://dx.doi.org/10.1016/j.urology.2013.07.039

98. Bonn SE, Sjolander A, Tillander A, Wiklund F, Gronberg H, Balter K. Body mass index in relation to serum prostate-specific antigen levels and prostate cancer risk. Int J Cancer [Internet]. 2016; Available from: http://www.ncbi.nlm.nih.gov/pubmed/26914149

99. Gray MA, Delahunt B, Fowles JR, Weinstein P, Cooke RR, Nacey JN. Demographic and clinical factors as determinants of serum levels of prostate specific antigen and its derivatives. Anticancer Res. 2004;24(3 B):2069–72.

100. Chang IH, Han JH, Ahn SH. Association of obesity with prostate specific antigen and prostate specific antigen velocity in healthy young men. J Urol [Internet]. 2008;179(3):881–6. Available from: http://www.ncbi.nlm.nih.gov/entrez/query.fcgi?cmd=Retrieve&db=PubMed&dopt=Citation&list_uids=18207169

101. Chia S-E, Lau WKO, Chin CM, Tan J, Ho SH, Lee J, et al. Effect of ageing and body mass index on prostate-specific antigen levels among Chinese men in Singapore from a community-based study. BJU Int [Internet]. 2009;103(11):1487–91. Available from: http://www.ncbi.nlm.nih.gov/pubmed/19076145

102. Loeb S, Carter HB, Schaeffer EM, Ferrucci L, Kettermann A, Metter EJ. Should Prostate Specific Antigen be Adjusted for Body Mass Index? Data From the Baltimore Longitudinal Study of Aging. J Urol. 2009;182(6):2646–52.

103. Chiu PK-F, Wong AY-F, Hou S-M, Yip SK-H, Ng C-F. Effect of body mass index on serum prostate-specific antigen levels among patients presenting with lower urinary tract symptoms. Asian Pac J Cancer Prev. 2011;12(8):1937–40.

104. Liu M, Wang J-Y, Zhu L, Wan G. Body mass index and serum lipid profile influence serum prostate-specific antigen in Chinese men younger than 50 years of age. Asian J Androl [Internet]. 2011;13(4):640–3. Available from: http://www.asiaandro.com/Abstract.asp?doi=10.1038/aja.2010.104

105. Wallner LP, Morgenstern H, McGree ME, Jacobson DJ, St. Sauver JL, Jacobsen SJ, et al. The Effects of Body Mass Index on Changes in Prostate-Specific Antigen Levels and Prostate Volume Over 15 Years of Follow-up: Implications for Prostate Cancer Detection. Cancer Epidemiol Biomarkers Prev. 2011;20(3):501–8.

106. Gómez-Guerra LS, Hernández-Torres AU, Blanco-Guzmán A, Solís-Rodríguez DE, Ortiz-Lara GE, Cortés-González JR. Effect of body mass index on PSA in northeast Mexican patients. Actas Urológicas Españolas (English Ed [Internet]. 2012;36(5):302–5. Available from: http://www.sciencedirect.com/science/article/pii/S2173578612001126

107. Ikuerowo SO, Omisanjo OA, Bioku MJ, Ajala MO, Esho JO. Effect of obesity on serum prostate-specific antigen in nigerian men. Urol Int. 2012;89(1):52–6.

108. Pater LE, Hart KW, Blonigen BJ, Lindsell CJ, Barrett WL. Relationship Between Prostate-specific Antigen, Age, and Body Mass Index in a Prostate Cancer Screening Population. Am J Clin Oncol [Internet]. 2012;35(5):490–2. Available from: http://content.wkhealth.com/linkback/openurl?sid=WKPTLP:landingpage&an=00000421-201210000-00015

109. Yang WJ. The likelihood of having a serum PSA level of ≥2.5 or ≥4.0 ng ml(-1) according to obesity in a screened Korean population. Asian J Androl [Internet]. 2013;15(6):770–2. Available from: http://www.scopus.com/inward/record.url?eid=2-s2.0-84887242982&partnerID=tZOtx3y1

110. Taghavi R, Aameli M, Jahed-Ataeian S, Hasanzade J. Relationship between body mass index and prostate specific antigen in patient with lower urinary tract symptoms. Urology [Internet]. 2014;84(4):S318–9. Available from: http://www.embase.com/search/results?subaction=viewrecord&from=export&id=L71654647

111. Adegun PT, Adebayo PB, Atiba SA. The likelihood of having serum level of PSA of ≥4.0 ng/mL and ≥10.0 ng/mL in non-obese and obese Nigerian men with LUTS. Asian J Urol. 2015;2(3):158–62.

112. Zhang J, Sheng B, Ma M, Nan X. An inverse association of obesity and prostate-specific antigen in elderly males. Int J Clin Exp Med. 2016;9(9):18746–53.

113. Yun J, Lee H, Yang W. Association between systemic inflammation and serum prostate-specific antigen in a healthy Korean population. Turkish J Urol. 2017;43(3):284–8.

114. Lacher DA, Hughes JP. Total, free, and complexed prostate-specific antigen levels among US men, 2007-2010. Clin Chim Acta [Internet]. 2015;448:220–7. Available from: http://www.sciencedirect.com/science/article/pii/S0009898115002922
